# Interaction effects of physicochemical factors on the growth of *Burkholderia pseudomallei* in soil microcosms

**DOI:** 10.1371/journal.pntd.0014339

**Published:** 2026-05-18

**Authors:** Kanokporn Chaianunporn, Supunnipa Wang-Ngarm, Pisit Chareonsudjai, Sorujsiri Chareonsudjai, Thotsapol Chaianunporn

**Affiliations:** 1 Faculty of Medicine, Mahasarakham University, Maha Sarakham, Thailand; 2 Thailand Environment Institute (TEI), Nonthaburi, Thailand; 3 Department of Environmental Science, Faculty of Science, Khon Kaen University, Khon Kaen, Thailand; 4 Department of Microbiology, Faculty of Medicine, Khon Kaen University, Khon Kaen, Thailand; Mahidol Univ, Fac Trop Med, THAILAND

## Abstract

**Background:**

*Burkholderia pseudomallei* (*B. pseudomallei*), the causative agent of melioidosis, inhabits diverse ecological niches such as soil, surface water, and rhizospheres. Soil serves as its primary reservoir. Understanding the ecological niche of *B. pseudomallei* in soil is crucial for developing strategies to reduce bacterial presence. Therefore, this study investigated the interaction effects of soil parameters, namely pH, salinity, temperature, moisture content, carbon to nitrogen (C/N) ratio, and iron content, on *B. pseudomallei* proliferation through factorial soil microcosm experiments.

**Methodology/principal findings:**

The experiments comprised three sets: (1) pH and salinity, (2) C/N ratio and salinity, and (3) iron content and salinity under varying temperature and moisture levels. Polynomial regression models demonstrated that all tested factors significantly influenced *B. pseudomallei* abundance, exhibiting linear, quadratic, cubic, and multi-way interactions. Optimal growth conditions varied with environmental context: *B. pseudomallei* growth peaked around pH 5 in the absence of salinity, whereas increased temperature and moisture expanded salinity tolerance (up to 0.61% at 35 °C and 75% moisture). The effect of C/N ratio on *B. pseudomallei* growth was moisture-dependent. The growth rate increased as the optimal C/N ratio rose from approximately 14 to 18 under 25 and 75% moisture, respectively, while elevated salinity consistently suppressed *B. pseudomallei* growth. Iron demonstrated contrasting effects depending on moisture; *B. pseudomallei* growth declined with increasing iron content at low-to-moderate moisture (25 and 50%) but was enhanced at high moisture (75%), with optimal iron content reaching 268.18 mg/kg at 35 °C.

**Conclusions/significance:**

These findings highlight that *B. pseudomallei* proliferation is governed by multifactorial interactions among soil physicochemical parameters. Understanding these interactions may support the development of environmental management strategies to reduce *B. pseudomallei* persistence in soil.

## Author summary

*Burkholderia pseudomallei* is the causative agent of melioidosis, a serious infectious disease endemic to tropical and subtropical regions. It primarily inhabits soil, where physicochemical properties strongly influence its persistence. This study investigated the interaction effects of pH, carbon-to-nitrogen (C/N) ratio, iron content and salinity under varying temperature and moisture conditions on *B. pseudomallei* proliferation using factorial microcosm experiments. The experiments comprised three sets: (1) pH and salinity, (2) C/N ratio and salinity, and (3) iron content and salinity under varying temperature and moisture levels. Polynomial regression models revealed that all parameters significantly influenced *B. pseudomallei* abundance. Optimal growth occurred under slightly acidic, moderate C/N ratio, and low-salinity conditions, whereas elevated temperature and moisture enhanced bacterial tolerance to both salinity and iron. These findings demonstrate that multiple environmental factors jointly shape the ecological niche of *B. pseudomallei*, emphasizing the importance of multifactorial approaches in assessing environmental persistence and melioidosis risk.

## Introduction

Melioidosis, caused by the Gram-negative bacterium *Burkholderia pseudomallei*, is a serious infectious disease endemic to tropical and subtropical regions, especially Southeast Asia and Northern Australia [1,2]. Transmission occurs via ingestion or inhalation of bacteria, or from bacteria entering skin abrasions [3,4]. *B. pseudomallei* has been detected in diverse ecological niches, including soil, surface water and legume roots [2,5–9].

Soil is the primary reservoir of *B. pseudomallei*, and its physicochemical properties are critical for bacterial survival. In Thailand, the highest incidence of melioidosis occurs in the northeast of the country [10,11]. The majority of patients are agricultural workers, particularly rice farmers, who are frequently exposed to contaminated soil and water [12,13]. Environmental factors linked to *B. pseudomallei* include soil moisture, which shows a positive correlation with the occurrence of *B. pseudomallei* in soil [14,15]. Both positive and negative associations have been reported between the presence of *B. pseudomallei* and high levels of iron, soil acidity, and soil salinity [1,14,16–18]. Land use and organic matter content also play important roles, as they influence soil biodiversity and may shape the ecological niche of *B. pseudomallei* [8,19,20]. Overall, soil type, texture, depth, moisture, salinity, and other physicochemical factors may influence the survival and persistence of *B. pseudomallei* [7,17,20–22].

Understanding the ecological niche and survival mechanisms of *B. pseudomallei* in soil is crucial for developing strategies to reduce bacterial presence and consequently disease prevalence—an aspect of melioidosis prevention that is equally important as clinical management. Wang-Ngarm et al. [17] investigated the effects of soil physicochemical properties on *B. pseudomallei* survival and growth in soil microcosms using soil from a *B. pseudomallei* positive site in northeast Thailand. Soil pH, salinity (% NaCl), iron, and C/N ratio were tested separately for 7 days. They reported that at 37 °C and 9% soil moisture the optimal growth of *B. pseudomallei* was observed at a pH of 6, salinity of 0.25%, an iron content of 125–150 mg/kg, and a C/N ratio of 15–20. However, the soil environment and its physicochemical properties are highly complex, and there is currently limited information on how multiple physicochemical factors interact to influence *B. pseudomallei* growth and survival. Therefore, this study explores the combined effects of soil physicochemical factors on the survival and growth of *B. pseudomallei* in soil microcosms with soil from an endemic area of northeastern Thailand, with the goal of understanding the impact of these factors on the persistence of *B. pseudomallei.* As soils in northeastern Thailand are characterized by varying degrees of salinity and iron content [17], this study examined the combined effects of salinity with other key parameters, namely pH, C/N ratio, and iron content under different temperature and moisture conditions to simulate seasonal variations influencing bacterial growth and survival. The experimental results were used to create polynomial regression models that included multi-way interactions to predict *B. pseudomallei* abundance under different parameter combinations. These models may provide valuable insights for developing environmental control strategies aimed at reducing infection risk through the manipulation of soil physicochemical conditions.

## Methods

### Soil samples

Soil samples were collected at a depth of 30 cm from Ban Kai Na (ST-39), Nam Phong District, Khon Kaen Province, Thailand, as previously described by Wang-Ngarm et al. [17]. This site has been identified as *B. pseudomallei*-positive and is representative of agricultural soils in melioidosis-endemic regions of northeastern Thailand [16,17]. The sampling depth of 30 cm was selected based on previous studies showing that *B. pseudomallei* is frequently detected in subsurface soils, where moisture and physicochemical conditions are relatively stable and supportive of bacterial persistence [7,15–17]. The soil samples were air-dried for 7 days and sieved to < 2 mm. The sieved soil was then stored in sealed plastic bags at 4 °C until it was used. Physicochemical properties of the soil, including texture, pH, electrical conductivity (EC), moisture content (MC), water-holding capacity (WHC), soil organic matter (SOM), soil organic carbon (SOC), total Kjeldahl nitrogen (TKN), available phosphorus (P_avail_), and concentrations of iron were subsequently determined.

### *B. pseudomallei* isolation and preparation

The *B. pseudomallei* strain used in this study was an environmental isolate obtained from soil in Ban Kai Na (ST-39), Nam Phong District, Khon Kaen Province, Thailand, as previously described by Wang-Ngarm et al. [17]. Presumptive identification was based on colony morphology on modified Ashdown’s agar, showing characteristic wrinkled or smooth purple-pink colonies. Confirmation was performed using standard biochemical tests, including triple sugar iron (TSI) agar, Augmentin/Colistin susceptibility (resistant to Colistin but sensitive to Augmentin), negative L-arabinose assimilation, and positive latex agglutination [17,23]. The strain was cultured overnight in 5 mL of Luria Bertani (LB) broth at 37 °C, adjusted to an OD_550_ of 0.1, diluted 1:100 in fresh LB broth and incubated at 37 °C with shaking at 200 rpm until reaching an OD_550_ of 0.8 (approximately 10⁸ CFU/mL), as confirmed by plate counting on modified Ashdown’s agar prior to inoculation into soil microcosms [17].

### Soil microcosm

One hundred grams of soil were placed into 250 mL glass bottles and sterilized by autoclaving at 121 °C and 15 psi for 15 minutes. Sterility was confirmed by suspending soil particles in a solution containing 2.5% (wt/vol) polyethylene glycol (PEG) and 0.1% (wt/vol) sodium deoxycholate (DOC), followed by plating on modified Ashdown’s agar [17,24]. As previously reported by Wang-Ngarm et al. [17], pH, salinity, and C/N ratio were identified as limiting factors for the growth of *B. pseudomallei*, whereas iron (Fe) availability was associated with increased bacterial numbers. Thus, treatment parameters in this study—including pH, salinity, iron content, C/N ratio, moisture content, and temperature—were systematically varied across experimental batches. Subsequently, 1 mL of *B. pseudomallei* inoculum (10⁸ CFU/mL) was evenly distributed over the soil surface in each bottle [17]. Duplicate microcosms were prepared for each treatment in three independent experimental sets.

### Soil pH

*B. pseudomallei* is capable of growth within a pH range of 5–7, with slightly reduced growth observed at pH 4, whereas pH 8 is associated with a marked reduction in bacterial numbers [17]. Its persistence in slightly acidic soils may account for its frequent occurrence in Thai rice fields, where soil pH typically ranges from 4.4 to 7.7 [11]. Therefore, a pH range of 4–8 was selected for this study. The pH of the soil samples was adjusted to approximately 4, 5, 6, 7, or 8 using 6 M H₂SO₄ or 6 M NaOH prior to autoclaving. The samples were left overnight, and pH values were subsequently monitored daily with a pH meter (EcoScan pH5; EUTECH, Singapore) at 1:1 (soil wt:water vol) [17,25].

### Soil salinity (Salt)

According to the Department of Land Development of Thailand, approximately 65% of soils in northeastern Thailand are classified as non-saline (0–2 dS/m), 12% as slightly saline (2–4 dS/m), and about 5% have salinity levels exceeding 4 dS/m. Increasing soil salinity above 2 dS/m has been associated with a reduction in *B. pseudomallei* populations [17]; however, salt application may adversely affect crop growth due to osmotic stress and toxicity. For example, rice cultivated in moderately saline soils (> 2 dS/m) can experience about 20% yield loss [26]. Therefore, salinity levels ranging from 0.02 to 4.68 dS/m were selected in this study to reflect regional soil conditions while considering agricultural impacts. Concentrated sodium chloride (NaCl) solution (AR grade; BDH Prolabo, VWR International, Lutterworth, UK) was added to dry soil samples to achieve salinity levels of 0, 0.3, 0.6, 0.9, and 1.2%NaCl (corresponding to 0.02, 1.39, 2.37, 3.72 and 4.68 dS/m, respectively). Soil salinity was measured using an electrical conductivity meter (CH-8603; Mettler Toledo, Switzerland) at a 1:5 (soil wt:water vol) ratio [17,27].

### Iron (Fe) content

Several studies have reported that iron, particularly under conditions of high water availability, significantly enhances the growth of *B. pseudomallei* [17,28–31]. In addition, *B. pseudomallei* has been detected in iron-rich soils [17,32]. Therefore, in the present study, soil with varying iron level was examined in combination with different soil moisture and temperature conditions to assess the relationship between physicochemical soil properties and the persistence of *B. pseudomallei*. Ferrous sulfate (FeSO₄) (AR grade; Ajax Chemicals Pty Limited, Auburn, NSW, Australia) was added to dry soil samples to obtain final iron concentrations of 50, 100, 200, 300, 400, and 500 mg/kg. The iron content of the samples was determined using the acid digestion method followed by flame atomic absorption spectrophotometry (Perkin-Elmer Analyst 300) [17,33].

### C/N ratio

C/N ratio is a key determinant of bacterial growth, as carbon serves as the primary energy source, whereas nitrogen is essential for protein and nucleic acid synthesis. A previously study reported that a C/N ratio of 15–20, indicative of nutrient-rich conditions, enhanced the growth of *B. pseudomallei* in soil microcosms [17]. In contrast, other studies have suggested that *B. pseudomallei* in natural environments may preferentially persist in nutrient-depleted soils [20,34]. Therefore, a range of C/N ratios representing both nutrient-rich (low C/N) and nutrient-depleted (high C/N) conditions was selected in this study (the C/N ratio was varied from 10, 25, 40, 55, to 70). The C/N ratio of the soil microcosms was adjusted using urea (AR grade; BDH Prolabo, VWR International, Lutterworth, UK) as the nitrogen source to achieve target ratios of 10, 25, and 40, while sterilized dried cow manure was applied as a carbon source to obtain higher C/N ratios of 55 and 70. Soil organic carbon was determined by the Walkley and Black method [35], while total Kjeldahl nitrogen was measured using the Kjeldahl method [17].

### Moisture content (MC)

To evaluate microbial responses across a moisture gradient, soil moisture levels of 25, 50, and 75% water-holding capacity (WHC) were selected to represent dry, intermediate, and wet conditions, respectively. The moisture content of the soil microcosms was adjusted to the desired levels using deionized water, and the initial weight of each bottle was recorded as previously described by Chen et al. [36]. During incubation, the bottles were covered with cotton and aluminum foil to minimize moisture loss, and their weights were monitored every two days to assess any changes in soil moisture [17].

### Temperature (Temp)

Soil temperatures of 25, 30, and 35 °C were selected to represent typical tropical soil conditions and to capture the thermal response range of *B. pseudomallei*, from suboptimal to near-optimal growth. The soil microcosms were incubated at 25, 30, and 35 °C under varying environmental factors, including pH, salinity, iron content, C/N ratio, and moisture content, prior to bacterial enumeration [17].

### Enumeration of *B. pseudomallei*

*B. pseudomallei* populations in soil microcosms were quantified at multiple time points (days 0–7) to monitor population dynamics using the PEG-DOC method [24]. Briefly, 200 mL of the PEG-DOC solution containing 2.5% (wt/vol) polyethylene glycol 6000 (AR grade; Merck KGaA, Darmstadt, Germany) and 0.1% (wt/vol) sodium deoxycholate (AR grade; Sigma-Aldrich, MO, USA) were added to 100 g of soil and shaken at 200 rpm for 2 h. The soil suspension was allowed to settle for 5 min, after which the bacterial suspension was serially diluted in sterile 0.9% NaCl. Each dilution was plated in 10 replicates on modified Ashdown’s agar using the drop plate method [37,38]. Colony counts obtained on day 7, representing the outcome of bacterial adaptation and persistence under the tested conditions, were subsequently used for polynomial regression modelling to assess the interaction effects of physicochemical factors on *B. pseudomallei* growth.

All soil microcosm experiments were conducted under strictly controlled laboratory conditions to minimize exposure risk. Procedures were performed by trained personnel using appropriate PPE, including laboratory gowns, gloves, and face masks, with standard aseptic techniques to minimize aerosol generation and environmental contamination. Contaminated materials and waste were decontaminated by autoclaving or appropriate disinfectants prior to disposal, ensuring complete inactivation of the organism. All personnel also underwent serological testing for *B. pseudomallei*-specific antibodies as part of routine exposure monitoring [39].

### Physicochemical factors affecting *B. pseudomallei* growth

To investigate the interaction effects of physicochemical factors on *B. pseudomallei* growth, soil microcosm experiments were performed under varying physicochemical conditions. The factors examined included soil pH, salinity, iron content, C/N ratio, moisture content, and temperature. The experimental design followed a complete factorial arrangement, in which temperature (25, 30 and 35 °C), moisture content (25, 50, and 75%), and selected paired factors were systematically varied, while the remaining parameters were maintained at their natural values (Table 1).

**Table 1:** Summary of physicochemical properties of soil from Ban Kai Na (ST-39), Nam Phong District, Khon Kaen Province, Thailand. EC: electrical conductivity, MC: moisture content, WHC: water holding capacity, SOM: soil organic matter, SOC: soil organic carbon, TKN: total Kjeldahl nitrogen, C/N ratio: carbon to nitrogen ratio, P_avail_: available phosphorus.

Soil parameters	Properties
Soil texture	Sandy loam
pH	6.3
EC	0.3 dS/m
MC	8.26%
WHC	22.49%
SOM	0.57%
SOC	0.33%
TKN	64.84 mg/kg
C/N ratio	50.90
P_avail_	0.14 mg/kg
Iron content	48 mg/kg

All soil microcosm investigations were conducted in three independent experiments, with duplicate samples for each treatment. The investigations were divided into three pairs as follows:

1] Effects of pH and salinity - pH was varied from 4, 5, 6, 7, to 8 and salinity from 0, 0.3, 0.6, 0.9, to 1.2% while the C/N ratio (50.90) and iron content (48 mg/kg) were kept constant. The number of values were equal to 5 pH levels × 5 salinity levels × 3 temperatures × 3 moisture contents × 3 independent experiments = 675 values.
2] Effects of C/N ratio and salinity - C/N ratio was varied from 10, 25, 40, 55, to 70 and salinity from 0, 0.3, 0.6, 0.9, to 1.2% where pH (6.3) and iron content (48 mg/kg) were kept constant. The number of values were equal to 5 C/N ratios × 5 salinity levels × 3 temperatures × 3 moisture contents × 3 independent experiments = 675 values.
3] Effects of iron content and salinity - iron content was varied from 50, 100, 200, 300, 400, to 500 mg/Kg and salinity from 0, 0.3, 0.6, 0.9, to 1.2% where pH (6.3) and C/N ratio (50.90) were kept constant. The number of values were equal to 6 iron contents × 5 salinity levels × 3 temperatures × 3 moisture contents × 3 independent experiments = 810 values.

### Statistical analysis

To investigate the combined effects of the above four factors on the number of *B. pseudomallei* colonies on day 7 (*N*: CFU/mL), we fitted a polynomial regression model including linear, quadratic, and cubic terms as well as multi-way interactions among predictors. The number of *B. pseudomallei* colonies (*N*) was first transformed using a base-10 logarithm (*Y*) to stabilize variance and reduce skewness:


$$\begin{array}{c} Y\;=\;{log}_{10}(N+0.01) \end{array}$$

Note that the number of *B. pseudomallei* colonies was always increased by 0.01 to avoid zero values when performing logarithmic transformations.

The initial model, referred to as the full cubic model, included all main effects, two-, three-and four-way interactions, and linear, quadratic and cubic terms for each factor. The model was fitted using ordinary least squares regression (“lm” function in R version 4.10). We applied stepwise model reduction by minimizing the Akaike Information Criterion (AIC) using the “step()” function in R. The result was a reduced cubic model that retained only significant predictors. To assess whether the reduced model was statistically different from the full cubic model, we performed a likelihood ratio test (analysis of variance or ANOVA) using the “anova()” function in R. The ANOVA test results were not significant (p > 0.05) in all three pairs studied. Thus, the reduced models were considered sufficient and selected as the final models, as it provided a more parsimonious description without loss of explanatory power. To evaluate whether the complexity of the final models was justified, they were compared with simpler models (linear and quadratic interaction models) using ANOVA and comparing AIC. In all cases, the simpler models lacking higher-order terms showed significantly poorer fit (p < 0.05) and had higher AIC, indicating that these terms in the final models captured meaningful structure in the data rather than overfitting (S1 Table). We further assessed model assumptions of the final models using standard residual diagnostics, including residuals versus fitted values, normal Q–Q plots, scale–location plots, and leverage plots. These indicated that residuals were approximately normally distributed, with no influential observations and only moderate deviations from homoscedasticity (S1–S3 Figs). We utilized the final models to predict the expected base-10 logarithm number of *B. pseudomallei* in each parameter combination.

## Results

### Effects of pH and salinity under different temperatures and moisture levels

**Fig 1:**
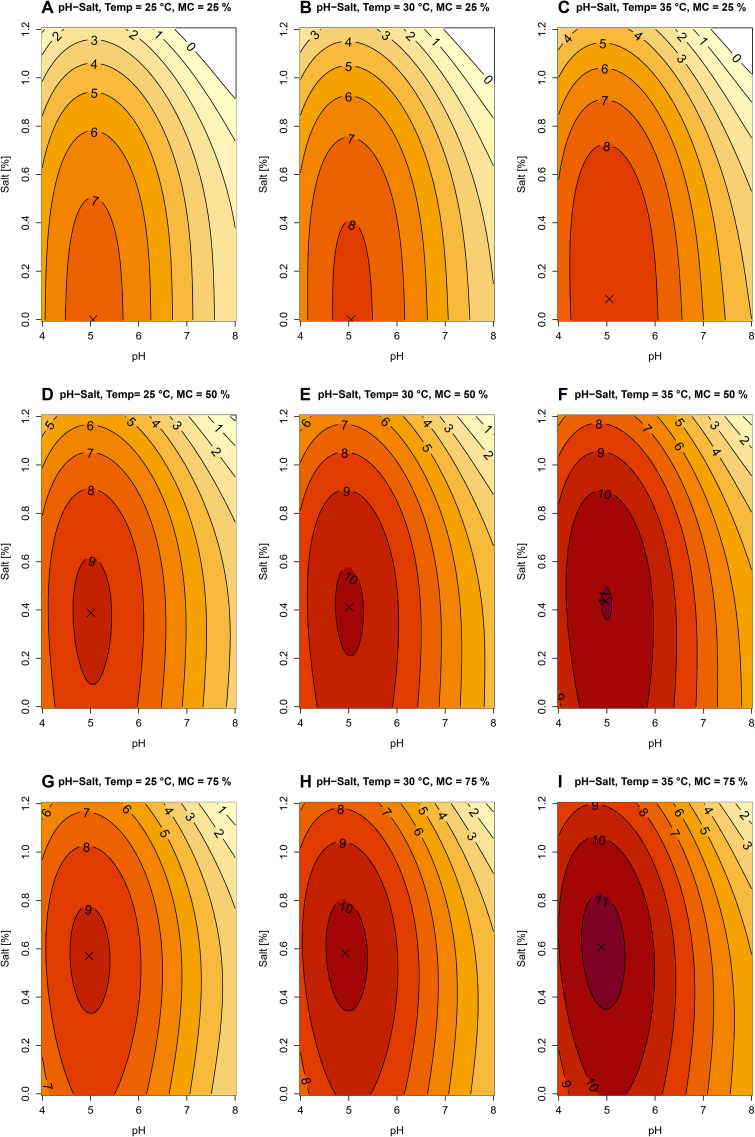
Predicted base-10 logarithm of *B. pseudomallei* number plotted over pH and salinity. Color gradients and contour lines represent model-predicted values. Panels A–C: moisture content (MC) = 25%, and temperature (Temp) = 25, 30 and 35 °C, respectively. Panels D–F: moisture content (MC) = 50%, and temperature (Temp) = 25, 30 and 35 °C, respectively. Panels G–I: moisture content (MC) = 75%, and temperature (Temp) = 25, 30 and 35 °C, respectively. Crosses indicate the optimal conditions, with the highest number of *B. pseudomallei* in each figure.

The model indicated that four factors (pH, salinity, temperature and moisture) exhibited linear, quadratic, cubic or multi-way interaction effects on the predicted number of *B. pseudomallei* (Table 2 and S4 Fig: residual standard error = 1.03 on 658 degrees of freedom; adjusted R^2^ = 0.88; F_16,658_ = 321.6; p < 2.2e-16). At the lowest temperature (25 °C) and moisture level (25%) examined, the highest number of *B. pseudomallei* was predicted at pH = 5.05 in the absence of salinity (Fig 1A). Deviations from this pH and/or increase in salinity resulted in a reduction in *B. pseudomallei* number (Fig 1A). Increasing temperature and/or moisture content generally promoted higher numbers of *B. pseudomallei* (Fig 1B – 1I). Interestingly, changes in temperature and/or moisture content shifted the optimal salinity (Fig 1A – 1I); higher temperature and moisture were associated with higher optimal salinity, increasing from 0% at 25 °C and 25% moisture content to 0.61% at 35 °C and 75% moisture content. The optimal pH, however, shifted only slightly, ranging from 4.89 to 5.05 (Fig 1A – 1I).

**Table 2:** Fit of the elements in the final model predicting effects of pH and salinity under different temperatures and moisture levels. Asterisks indicate statistical significance at α = 0.05 (Salt: salinity of soil, Temp: temperature of soil, MC: moisture content of soil).

	Estimate	Std. Error	T value	*p*
(Intercept)	-59.23	5.27	-11.25	<2.00E-16*
pH	29.64	2.50	11.88	<2.00E-16*
Salt	-8.66	3.20	-2.71	0.007*
Temp	8.91E-02	7.73E-02	1.15	0.25
MC	0.16	2.46E-02	6.66	5.96E-11*
pH^2^	-4.66	0.42	-11.04	<2.00E-16*
MC^2^	-1.69E-03	1.35E-04	-12.50	<2.00E-16*
pH^3^	0.22	2.34E-02	9.58	<2.00E-16*
Salt^3^	-2.72	0.15	-18.83	<2.00E-16*
pH:Salt	1.27	0.52	2.46	0.014*
pH:Temp	9.26E-03	1.19E-02	0.78	0.44
pH:MC	6.80E-04	2.38E-03	0.29	0.78
Salt:Temp	0.24	0.10	2.39	0.02*
Salt:MC	9.65E-02	2.00E-02	4.82	1.77E-06*
Temp:MC	6.93E-04	4.77E-04	1.45	0.15
pH:Salt:Temp	-4.22E-02	1.62E-02	-2.60	0.01*
pH:Salt:MC	-7.97E-03	3.25E-03	-2.46	0.014*

### Effects of C/N ratio and salinity under different temperatures and moisture levels

**Fig 2:**
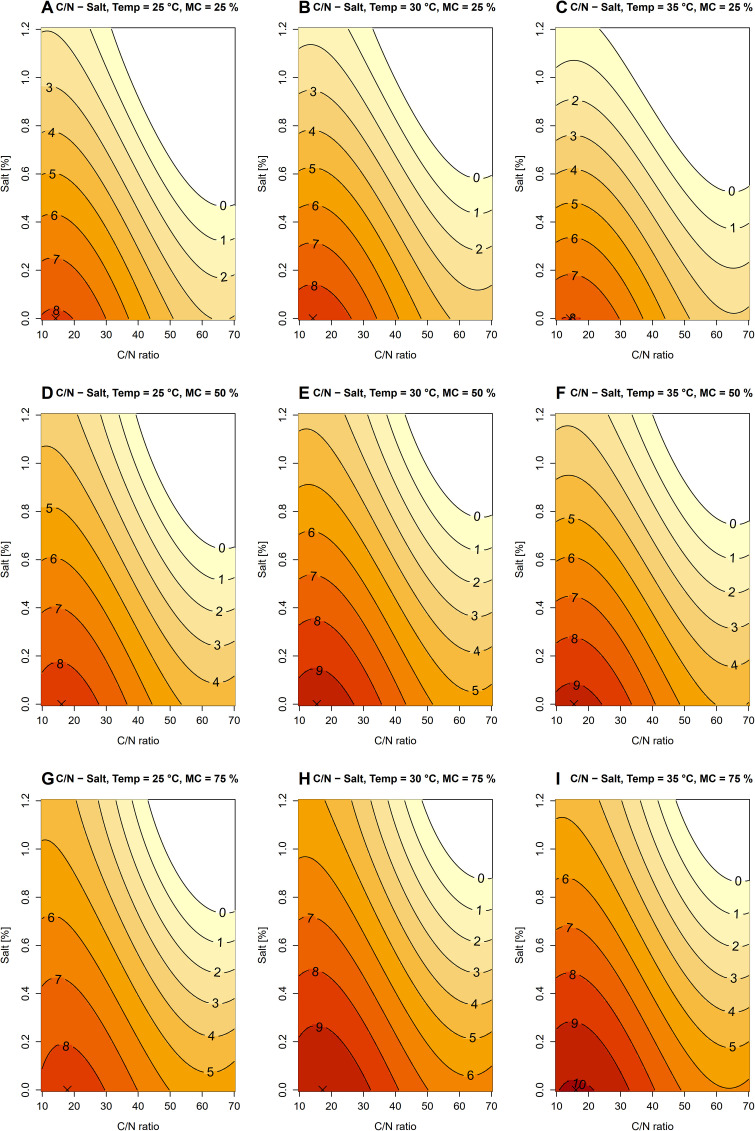
Predicted base-10 logarithm of *B. pseudomallei* number plotted over C/N ratio and salinity. Color gradients and contour lines represent model-predicted values. Panels A–C: moisture content (MC) = 25%, and temperature (Temp) = 25, 30 and 35 °C, respectively. Panels D–F: moisture content (MC) = 50%, and temperature (Temp) = 25, 30 and 35 °C, respectively. Panels G–I: moisture content (MC) = 75%, and temperature (Temp) = 25, 30 and 35 °C, respectively. Crosses indicate the optimal conditions with the highest number of *B. pseudomallei* in each figure.

The model revealed significant associations of C/N ratio, salinity, temperature, and moisture with the predicted number of *B. pseudomallei* (Table 3 and S5 Fig: residual standard error = 1.42 on 655 degrees of freedom; adjusted R^2^ = 0.85; F_19,655_ = 208.9; p < 2.2e-16). In all parameter combinations, the optimal salinity was consistently 0%, and any increase in salinity resulted in a reduction in the number of *B. pseudomallei* (Fig 2A – 2I). At the lowest moisture level (25%), the highest number of *B. pseudomallei* was predicted at C/N ratio = 14.24 in all tested temperatures (Fig 2A – 2C). With increasing moisture content, the optimal C/N ratio shifted upward, ranging from 15.45 to 16.06 at 50% moisture and from 16.06 to 17.88 at 75% moisture (Fig 2D – 2I). Increases or decreases in the C/N ratio away from the optimal value generally reduced the number of *B. pseudomallei* except at high C/N ratio (C/N ratio > 60), where the number of *B. pseudomallei* appeared relatively stable. Temperature effects varied with moisture content, with the highest number of *B. pseudomallei* observed at 30 °C under 25 and 50% moisture (Fig 2B and 2E) and at 35 °C under 75% moisture (Fig 2I).

**Table 3:** Fit of the elements in the final model predicting effects of C/N ratio and salinity under different temperatures and moisture levels. Asterisks indicate statistical significance at α = 0.05 (C/N: carbon to nitrogen ratio of soil, Salt: salinity of soil, Temp: temperature of soil, MC: moisture content of soil).

	Estimate	Std. Error	T value	*p*
(Intercept)	-11.49	4.88	-2.35	0.019*
C/N	0.14	7.04E-02	1.97	0.049*
Salt	-0.49	2.37	-0.21	0.84
Temp	1.30	0.29	4.47	9.22E-06*
MC	-6.95E-02	4.69E-02	-1.48	0.14
(C/N)^2^	-8.98E-03	1.15E-03	-7.79	2.55E-14*
Salt^2^	-3.47	2.17	-1.60	0.11
Temp^2^	-2.38E-02	4.63E-03	-5.14	3.58E-07*
MC^2^	-4.70E-04	1.85E-04	-2.54	0.011*
(C/N)^3^	7.49E-05	9.52E-06	7.87	1.48E-14*
Salt^3^	1.97	1.19	1.66	0.098
C/N:Salt	-8.51E-02	4.73E-02	-1.80	0.073
C/N:Temp	1.88E-03	1.89E-03	1.00	0.320
C/N:MC	2.24E-03	9.51E-04	2.36	0.019*
Salt:Temp	-1.75E-01	6.72E-02	-2.61	0.009*
Salt:MC	5.46E-02	1.34E-02	4.06	5.42E-05*
Temp:MC	4.39E-03	1.40E-03	3.14	0.002*
C/N:Salt:Temp	3.48E-03	1.48E-03	2.35	0.019*
C/N:Salt:MC	-1.09E-03	2.97E-04	-3.67	0.0003*
C/N:Temp:MC	-5.44E-05	3.09E-05	-1.76	0.078

### Effects of iron content and salinity under different temperatures and moisture levels

**Fig 3:**
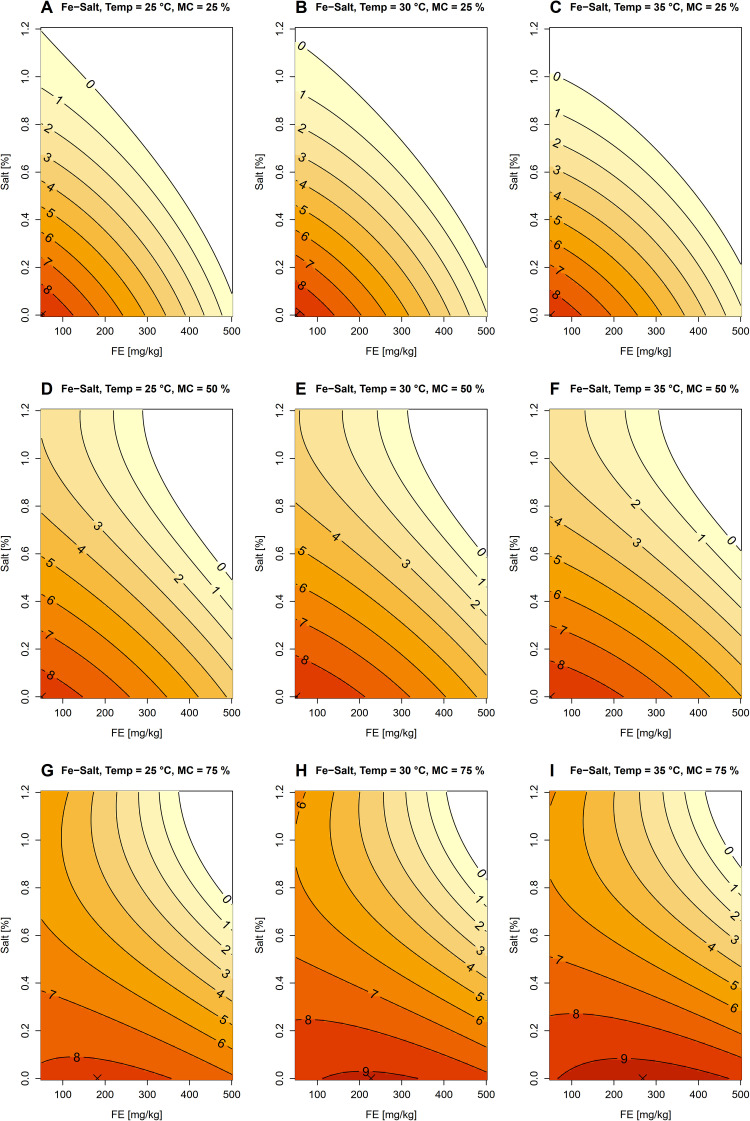
Predicted base-10 logarithm of *B. pseudomallei* number plotted over iron content and salinity. Color gradients and contour lines represent model-predicted values. Panels A–C: moisture content (MC) = 25%, and temperature (Temp) = 25, 30 and 35 °C, respectively. Panels D–F: moisture content (MC) = 50%, and temperature (Temp) = 25, 30 and 35 °C, respectively. Panels G–I: moisture content (MC) = 75%, and temperature (Temp) = 25, 30 and 35 °C, respectively. Crosses indicate the optimal conditions with the highest number of *B. pseudomallei* in each figure.

Iron content, salinity, temperature and moisture were also significantly associated with the predicted number of *B. pseudomallei* (Table 4 and S6 Fig: residual standard error = 1.65 on 794 degrees of freedom; adjusted R^2^ = 0.83; F_15,794_ = 255.7; p < 2.2e-16). In all parameter combinations, the optimal salinity was consistently 0% (Fig 3A – 3I). Increases in salinity reduced the number of *B. pseudomallei* at 25 and 50% moisture content (Fig 3A – 3F), whereas at 75% moisture content, the number of *B. pseudomallei* tended to stabilize when salinity exceeded 0.6% (Fig 3G – 3I). At 25 and 50% moisture levels, the highest number of *B. pseudomallei* was observed at 0 mg/kg iron content at all temperatures, with increasing iron content leading to a decline in *B. pseudomallei* number (Fig 3A – 3F). In contrast, at the 75% moisture level, the optimal iron content shifted upward with increasing temperature, occurring at 181.82, 227.27, and 268.18 mg/kg at 25, 30, and 35 °C, respectively (Fig 3G – 3I).

**Table 4:** Fit of the elements in the final model predicting effects of iron contents and salinity under different temperatures and moisture levels. Asterisks indicate statistical significance at α = 0.05 (FE: iron content of soil, Salt: salinity of soil, Temp: temperature of soil, MC: moisture content of soil).

	Estimate	Std. Error	T value	*p*
(Intercept)	3.91	4.60	0.85	0.395
FE	-2.64E-02	3.52E-03	-7.52	1.50E-13*
Salt	-10.60	1.63	-6.52	1.23E-10*
Temp	0.52	0.30	1.73	0.085
MC	-8.21E-02	2.28E-02	-3.60	0.0003*
FE^2^	-1.20E-05	2.90E-06	-4.13	3.97E-05*
Salt^2^	-3.90	2.30	-1.69	0.091
Temp^2^	-9.84E-03	4.91E-03	-2.01	0.045*
Salt^3^	3.51	1.26	2.78	0.006*
FE:Salt	1.89E-02	2.27E-03	8.35	3.06E-16*
FE:Temp	2.12E-04	8.90E-05	2.38	0.018*
FE:MC	3.40E-04	3.08E-05	11.03	<2e-16*
Salt:Temp	-4.75E-02	3.34E-02	-1.42	0.155
Salt:MC	0.14	1.27E-02	10.82	<2e-16*
Temp:MC	1.86E-03	6.94E-04	2.68	0.008*
FE:Salt:MC	-4.48E-04	4.19E-05	-10.69	<2e-16*

## Discussion

Variations in soil physicochemical parameters including pH, temperature, moisture and salinity have been reported to significantly influence the prevalence of *B. pseudomallei*. The previous work demonstrated that, at a soil temperature of 37 °C and soil moisture level of 9%, the optimal point for *B. pseudomallei* numbers is at pH 6 and 0.25% NaCl. Also, bacterial growth was not sustained in soil containing more than 0.7% NaCl [17]. Given the complexity of the soil environment, a combined-physicochemical parameter analysis can generate a model that more accurately reflects natural conditions, where multiple environmental factors interact with one another. This approach may provide a more realistic and comprehensive representation than single-factor analyses. Accordingly, this study investigated how interactions between multiple physicochemical factors (pH, C/N ratios, iron content, salinity, soil moisture and temperature) influence the optimal conditions for the survival and growth of *B. pseudomallei* in soil microcosms.

The current study examined the combined effects of pH and salinity under different temperatures and moisture levels and revealed shifts in the optimal physiochemical factors when temperature and moisture level are changed. The optimal soil pH for *B. pseudomallei* growth in this study was consistently slightly acidic**,** ranging from 4.89 to 5.05 across all combined conditions. In contrast, bacterial growth declined as soil pH increased, indicating that alkaline conditions negatively affect bacterial proliferation. These findings suggest that *B. pseudomallei* is better adapted to slightly acidic soil environments**,** while increases in soil pH may limit its growth and persistence. Previous studies have similarly reported that *B. pseudomallei* grows well in soils with a pH range of approximately 5–7 [11,15,17,36], whereas increasing soil pH to 8 significantly reduces bacterial numbers [17]. Consistent with these observations, our results demonstrate that even modest increases in soil pH can suppress bacterial growth, highlighting soil alkalinity as an important inhibitory factor for *B. pseudomallei*. Therefore, this study provides additional evidence that alkaline soil conditions restrict the persistence of *B. pseudomallei* and further refines the understanding of the pH range that supports or limits its survival under combined soil physicochemical conditions.

As high temperature and high soil moisture have previously been identified as a factor promoting the growth of *B. pseudomallei* [7,8,12,40–42], our models predicted that under more favorable conditions of 35 °C, 75% soil moisture, and approximately pH 5, the optimal salinity shifted upward to nearly 0.7%, supporting *B. pseudomallei* growth up to 10¹¹ CFU/mL, compared with approximately 10⁷ CFU/mL at 25 °C and 25% soil moisture (at approximately pH 5 and 0% salinity). While moderate to high salinity (from 0.7% up to 5% NaCl) can inhibit *B. pseudomallei* growth in soil [17], *B. pseudomallei* is able to survive in saline solutions at concentrations up to 2.5% and in groundwater with elevated salinity during periods of heavy rainfall [21,28]. Our results suggest that increases in moisture level, for example, during the rainy season, are likely to enhance bacterial growth, and expand the salinity threshold of *B. pseudomallei*, thereby facilitating its environmental persistence and proliferation. These findings are in alignment with previous reports of *B. pseudomallei* being detected at higher levels in soil during the rainy season [34] and support the idea that rainfall-driven changes in soil conditions play a key role in shaping the environmental distribution of *B. pseudomallei*. Furthermore, these results suggest that ongoing global change, which is associated with higher temperature, increased climate variability and more frequent extreme rainfall events, may contribute to the expansion of *B. pseudomallei* distribution and potential exposure risk in affected regions [40].

C/N ratio is a critical determinant of *B. pseudomallei* growth, as carbon provides the primary energy source and nitrogen is required for protein and nucleic acid synthesis [43]. In the present study, when the C/N ratio was evaluated in combination with salinity under varying temperatures and moisture levels, *B. pseudomallei* growth was enhanced at C/N ratios ranging from 15 to 18. These results are consistent with the findings of Wang-Ngarm et al. [17], who reported *B. pseudomallei* proliferation at C/N ratios of 10–25, with a marked reduction at low nitrogen ratio (C/N ratio = 40). Interestingly, under combined C/N ratio and salinity conditions, the optimal salinity for *B. pseudomallei* growth was 0%, with any increase in salinity resulting in reduced predicted bacterial numbers. In contrast to our findings, previous field studies have reported that *B. pseudomallei* in natural environments persist preferentially in nutrient-depleted soils [20,34] while C/N ratios ranging from 15 to 18 are considered good for general microbial growth, neither excessively nitrogen-limited nor prone to rapid nutrient loss. The differences between this study and previous studies may be explained by the complexity of bacterial communities in natural soil. In nutrient-rich soils, competition between bacteria is likely to be strong and may limit the proliferation of *B. pseudomallei*, whereas in nutrient-depleted soils, reduced competition may allow *B. pseudomallei* to become dominant as *B. pseudomallei* is more resistant to environmental stress [44].

Iron is a critical micronutrient for microbial metabolism, functioning as a cofactor in energy generation, DNA synthesis, and redox reactions [45–48]. Several studies have reported that iron significantly enhances the growth of *B. pseudomallei* and is strongly associated with high water availability [17,28,29,31,49]. This may be linked to changes in iron speciation under different redox conditions: high soil moisture and low oxygen promote reducing conditions (Fe²⁺ or ferrous iron) which is more soluble and bioavailable for bacteria. In contrast, under drier and more oxygen-rich conditions, iron predominantly exists as ferric iron (Fe³⁺), which is poorly soluble, often bound in mineral phases and less accessible to bacteria [50,51]. In addition, iron may act as an alternative energy source under anoxic conditions, with water saturation generating bioavailable forms that facilitate bacterial persistence [29,30].

Consistent with these mechanisms, we observed that at low moisture levels across all temperatures, increases in iron content led to decreased numbers of *B. pseudomallei*. In contrast, under high soil moisture (75%), the optimal iron concentration for *B. pseudomallei* proliferation shifted upward with increasing temperature. Our findings further suggest that elevated temperature and moisture may enhance the metabolic capacity of *B. pseudomallei* to utilize iron as a growth-promoting factor, whereas under drier conditions, excess iron may exert inhibitory effects. However, as iron speciation and redox potential were not directly measured in this study, this mechanism remains speculative.

Our findings could identify the key physicochemical drivers of *B. pseudomallei* growth and persistence; however, translating these observations into practical environmental mitigation strategies remains inherently challenging. Direct eradication of the organism from natural environments is currently unfeasible due to its widespread distribution and considerable ecological adaptability. Potential strategies may include environmental management interventions such as improved drainage to minimize prolonged soil saturation [29,52] alongside targeted modifications of soil physicochemical properties; for example, applications of specific fertilizers to alter the carbon-to-nitrogen (C/N) ratio or quicklime (calcium oxide) to elevate soil pH [14,53]. These recommendations are supported by our predictive models, which indicate that the interaction of low soil moisture with either a high C/N ratio or elevated pH significantly reduces *B. pseudomallei* abundance. Alternatively, increase in soil salinity in combination with elevated pH, C/N ratios or iron contents may serve as an effective strategy to reduce number of *B. pseudomallei*. Nevertheless, because salt application adversely affects crop yield [26], such interventions are primarily viable only in non-agricultural areas, such as zoos [54].

While this study provides insights into the influence of physicochemical soil parameters on the survival of an environmental *B. pseudomallei* isolate, the use of a single strain without genetic characterization should be considered when interpreting broader applicability. Given the substantial genomic diversity of *B. pseudomallei* across regions and ecological niches, variation in environmental fitness and persistence among strains is likely [55]. Thus, although the observed trends likely reflect fundamental responses to key soil conditions, their magnitude may differ across *B. pseudomallei* lineages. The lack of molecular typing data limits direct comparison with other environmental and clinical isolates and makes it more challenging to position this strain within the global population structure [55–57]. Despite this limitation, our controlled experimental models provide a clear and useful baseline for understanding how physicochemical factors influence *B. pseudomallei* survival. Future studies integrating genomic characterization with similar experimental approaches will be important to determine whether these findings are consistent across diverse strains and to strengthen their broader relevance.

In conclusion, this study demonstrates that *B. pseudomallei* abundance in soil is governed by complex interactions among soil physicochemical factors. Optimal growth occurred under slightly acidic conditions, moderate C/N ratio, and low salinity, while elevated temperature and moisture enhanced bacterial tolerance to salinity and iron. The interactions among these factors further indicate that multiple environmental factors jointly shape the ecological niche of *B. pseudomallei*, supporting the importance of multifactorial approaches in assessing melioidosis risk.

Our findings are likely representative of conditions in northeastern Thailand, where environmental characteristics, soil properties, and agricultural practices are comparable to those examined in this study (see “Methods” section for more details). Furthermore, because key drivers of *B. pseudomallei* persistence—such as soil moisture, salinity, temperature, and nutrient availability—are common across many rice-growing regions, our findings may also be applicable to other parts of Southeast Asia with similar tropical agricultural conditions. Overall, our results suggest that managing soil conditions, including reducing water saturation, maintaining moderate to high pH, and regulating nutrient and iron levels may help limit *B. pseudomallei* proliferation in endemic areas. Environmental management strategies combined with the promotion of protective behaviors [12,58] could therefore complement clinical and public health measures to reduce melioidosis incidence.

However, the applicability of these findings may be limited in areas with substantially different soil types, hydrological regimes, or land-use practices, as such factors can influence bacterial dynamics. Moreover, the experiments were conducted under controlled microcosm conditions designed to investigate the effects of soil physicochemical factors on *B. pseudomallei* growth. Consequently, this system does not fully represent natural soil environments where complex microbial communities and biotic interactions may influence bacterial dynamics. Therefore, the results of the present study should be interpreted as reflecting the growth potential and responses of *B. pseudomallei* to environmental conditions under controlled settings, rather than direct measures of ecological persistence in natural soils. In addition, while this study is limited to soil microcosms, *B. pseudomallei* can also be present in aquatic environments. Future research investigating the influence of physicochemical water properties on the proliferation and persistence of *B. pseudomallei* would be highly relevant because contaminated water serves as a significant route of human exposure [12,59,60].

## Supporting information

S1 Fig Standard residual diagnostics for the reduced cubic interaction model (final model) predicting the effects of pH and salinity under varying temperature and moisture conditions, including (A) residuals versus fitted values, (B) normal Q–Q plots, (C) scale–location plots, and (D) leverage plots. (DOCX)

S2 Fig Standard residual diagnostics for the reduced cubic interaction model (final model) predicting the effects of C/N ratios and salinity under varying temperature and moisture conditions, including (A) residuals versus fitted values, (B) normal Q–Q plots, (C) scale–location plots, and (D) leverage plots. (DOCX)

S3 Fig Standard residual diagnostics for the reduced cubic interaction model (final model) predicting the effects of iron contents and salinity under varying temperature and moisture conditions, including (A) residuals versus fitted values, (B) normal Q–Q plots, (C) scale–location plots, and (D) leverage plots. (DOCX)

S4 Fig Predicted and observed effects of pH and salinity on *B. pseudomallei* growth under different temperature and soil moisture conditions. Panels A–C: moisture content (MC) = 25%, and temperature (Temp) = 25, 30 and 35 °C, respectively. Panels D–F: moisture content (MC) = 50%, and temperature (Temp) = 25, 30 and 35 °C, respectively. Panels G–I: moisture content (MC) = 75%, and temperature (Temp) = 25, 30 and 35 °C, respectively. Colored lines indicate model-predicted values at different pH (4–8), while points represent observed mean colony counts (log₁₀ (CFU + 0.01)) with error bars showing standard error.
(DOCX)

S5 Fig Predicted and observed effects of C/N ratio and salinity on *B. pseudomallei* growth under different temperature and soil moisture conditions. Panels A–C: moisture content (MC) = 25%, and temperature (Temp) = 25, 30 and 35 °C, respectively. Panels D–F: moisture content (MC) = 50%, and temperature (Temp) = 25, 30 and 35 °C, respectively. Panels G–I: moisture content (MC) = 75%, and temperature (Temp) = 25, 30 and 35 °C, respectively. Colored lines indicate model-predicted values at different C/N ratios (10–70), while points represent observed mean colony counts (log₁₀ (CFU + 0.01)) with error bars showing standard error.
(DOCX)

S6 Fig Predicted and observed effects of iron contents (FE) and salinity on *B. pseudomallei* growth under different temperature and soil moisture conditions. Panels A–C: moisture content (MC) = 25%, and temperature (Temp) = 25, 30 and 35 °C, respectively. Panels D–F: moisture content (MC) = 50%, and temperature (Temp) = 25, 30 and 35 °C, respectively. Panels G–I: moisture content (MC) = 75%, and temperature (Temp) = 25, 30 and 35 °C, respectively. Colored lines indicate model-predicted values at different iron concentrations (50–500 mg/kg), while points represent observed mean colony counts (log₁₀ (CFU + 0.01)) with error bars showing standard error.
(DOCX)

S1 Table Comparison of Akaike Information Criterion (AIC) values and pairwise analysis of variance (ANOVA) results among the reduced cubic interaction model (final model), the full cubic interaction model, the quadratic interaction model, and the linear interaction model. (DOCX)

S2 Table Number of *B. pseudomallei* colonies on day 7 (N, CFU/mL) in three replicates under varying pH and salinity conditions at different soil temperatures and moisture contents, with C/N ratio and iron content kept constant (Salt: salinity of soil, Temp: temperature of soil, MC: moisture content of soil, C/N ratio: carbon to nitrogen ratio of soil, FE: iron content of soil). (DOCX)

S3 Table Number of *B. pseudomallei* colonies on day 7 (N, CFU/mL) in three replicates under varying C/N ratio and salinity conditions at different soil temperatures and moisture contents, with pH and iron content kept constant (Salt: salinity of soil, Temp: temperature of soil, MC: moisture content of soil, C/N ratio: carbon to nitrogen ratio of soil, FE: iron content of soil). (DOCX)

S4 Table Number of *B. pseudomallei* colonies on day 7 (N, CFU/mL) in three replicates under varying iron content and salinity conditions at different soil temperatures and moisture contents, with pH and iron content kept constant (Salt: salinity of soil, Temp: temperature of soil, MC: moisture content of soil, C/N ratio: carbon to nitrogen ratio of soil, FE: iron content of soil). (DOCX)
