# Supplementary material for: Interaction effects of physicochemical factors on the growth of Burkholderia pseudomallei in soil microcosms
Source: PLoS Negl Trop Dis. 2026 May 18;20(5):e0014339. doi: 10.1371/journal.pntd.0014339 (PMC13197065; doi:10.1371/journal.pntd.0014339)
Supplement: S1 Fig — (DOCX) [file pntd.0014339.s001.docx]

**
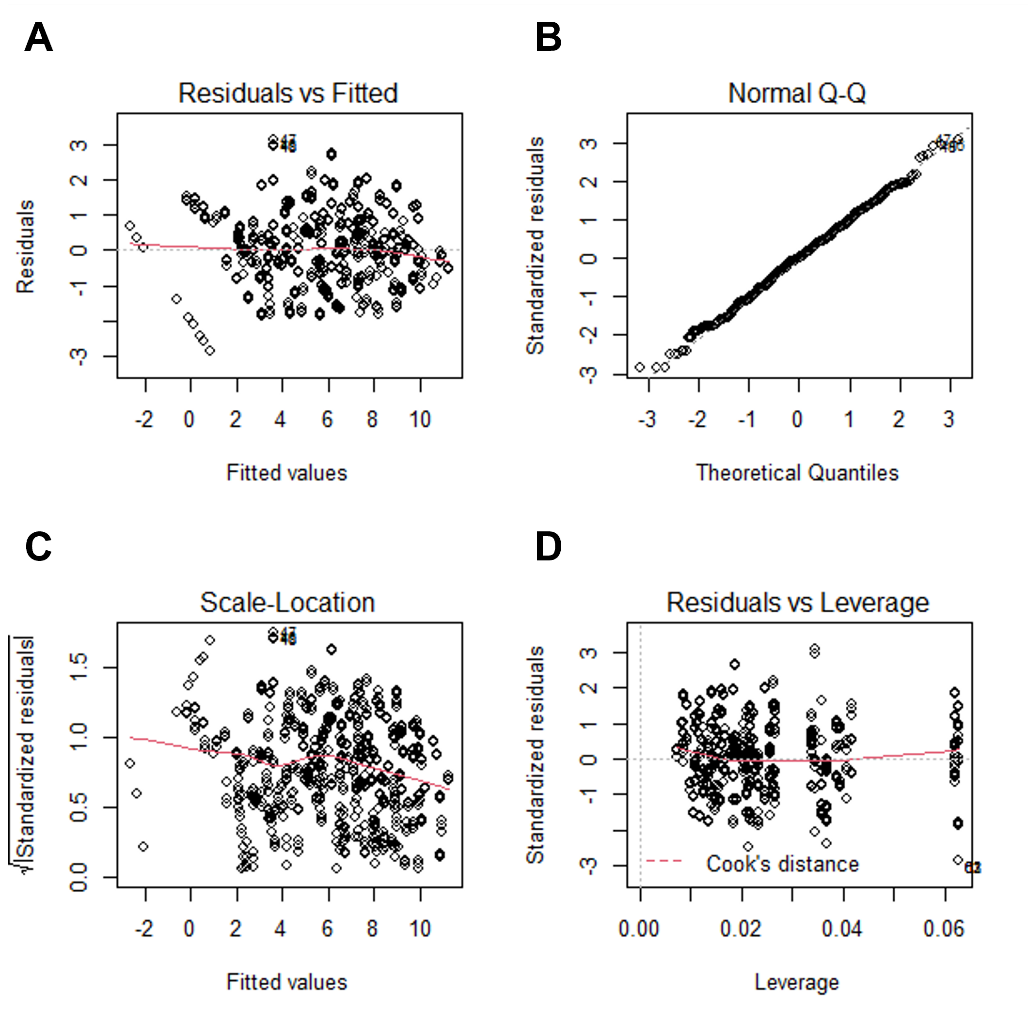
**

**S1 Fig.** Standard residual diagnostics for the reduced cubic interaction model (final model) predicting the effects of pH and salinity under varying temperature and moisture conditions, including (A) residuals versus fitted values, (B) normal Q–Q plots, (C) scale–location plots, and (D) leverage plots.
