# Supplementary material for: Interaction effects of physicochemical factors on the growth of Burkholderia pseudomallei in soil microcosms
Source: PLoS Negl Trop Dis. 2026 May 18;20(5):e0014339. doi: 10.1371/journal.pntd.0014339 (PMC13197065; doi:10.1371/journal.pntd.0014339)
Supplement: S2 Fig — (DOCX) [file pntd.0014339.s002.docx]

**
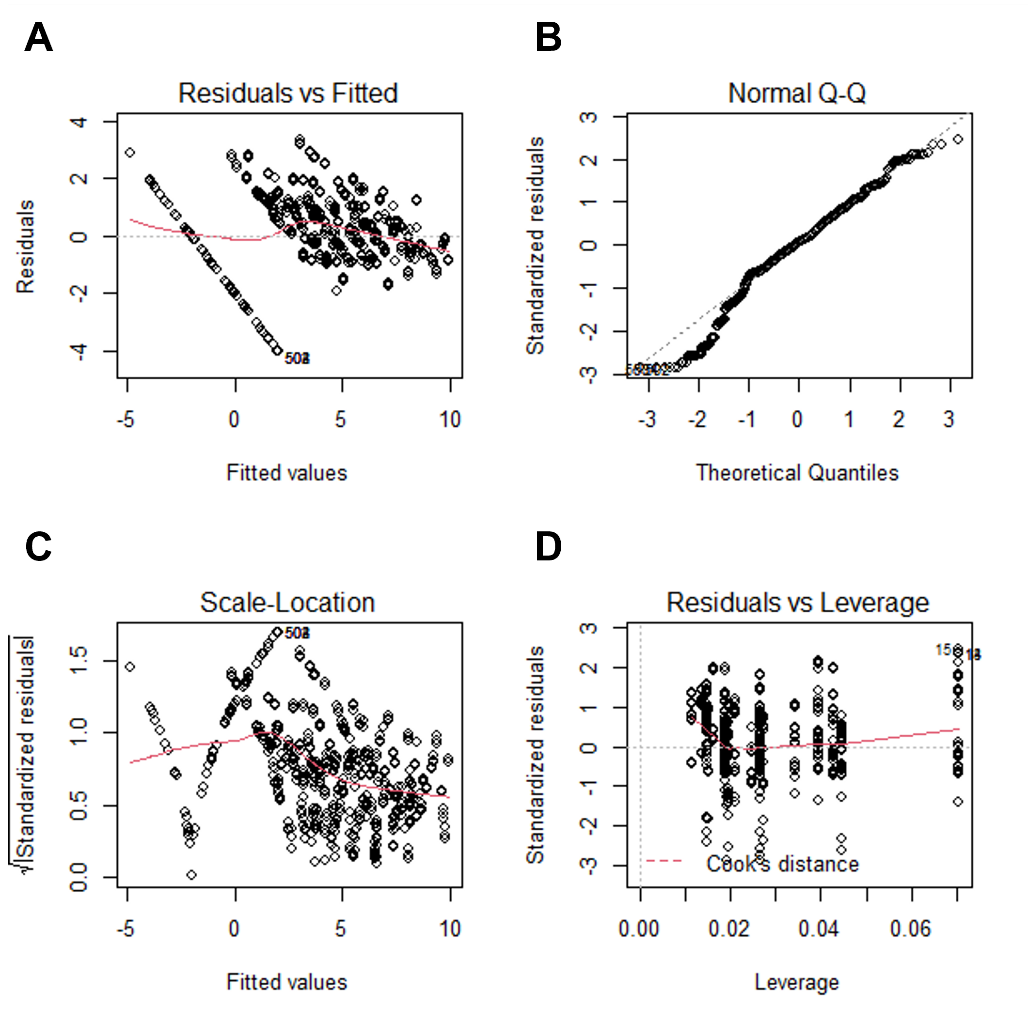
**

**S2 Fig.** Standard residual diagnostics for the reduced cubic interaction model (final model) predicting the effects of C/N ratios and salinity under varying temperature and moisture conditions, including (A) residuals versus fitted values, (B) normal Q–Q plots, (C) scale–location plots, and (D) leverage plots.
