# Supplementary material for: Interaction effects of physicochemical factors on the growth of Burkholderia pseudomallei in soil microcosms
Source: PLoS Negl Trop Dis. 2026 May 18;20(5):e0014339. doi: 10.1371/journal.pntd.0014339 (PMC13197065; doi:10.1371/journal.pntd.0014339)
Supplement: S5 Fig — Panels A–C: moisture content (MC) = 25%, and temperature (Temp) = 25, 30 and 35 °C, respectively. Panels D–F: moisture content (MC) = 50%, and temperature (Temp) = 25, 30 and 35 °C, respectively. Panels G–I: moisture content (MC) = 75%, and temperature (Temp) = 25, 30 and 35 °C, respectively. Colored lines indicate model-predicted values at different C/N ratios (10–70), while points represent observed mean colony counts (log₁₀ (CFU + 0.01)) with error bars showing standard error. (DOCX) [file pntd.0014339.s005.docx]

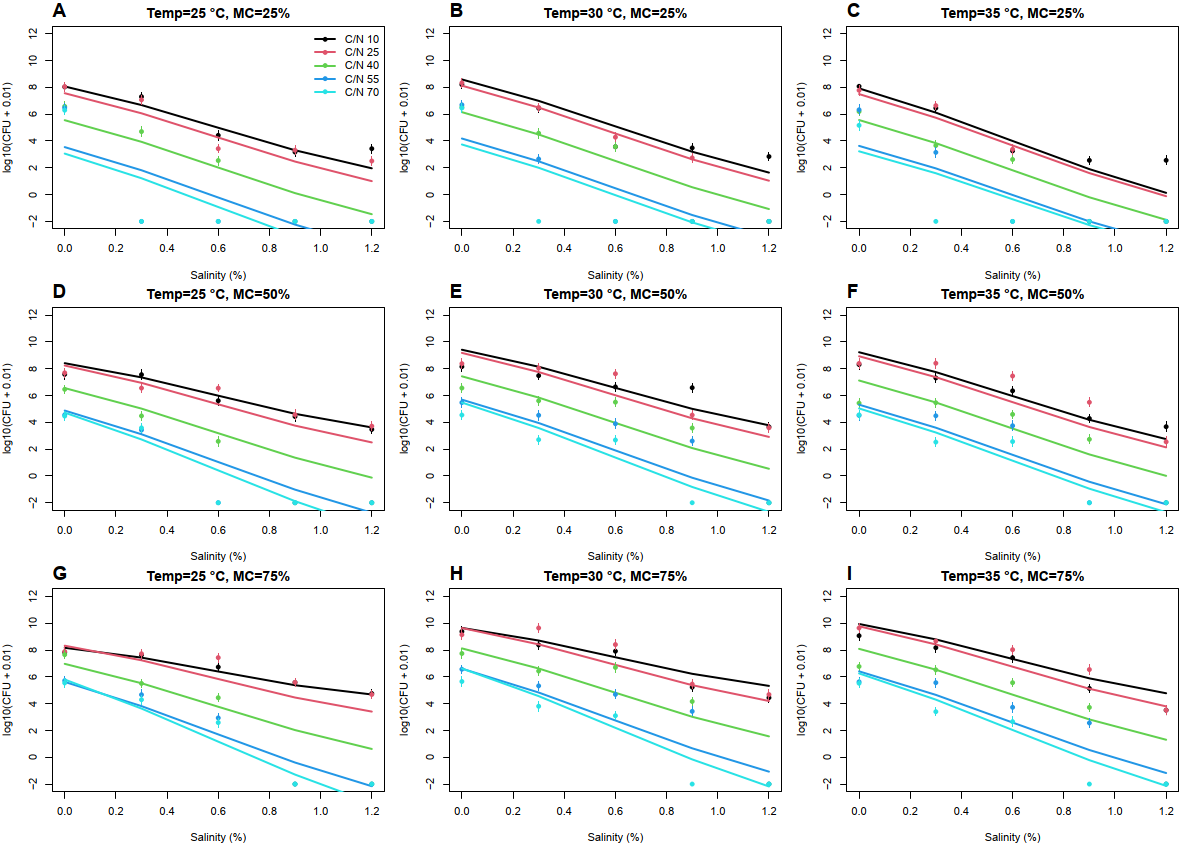


**S5 Fig.**  Predicted and observed effects of C/N ratio and salinity on *B. pseudomallei* growth under different temperature and soil moisture conditions. Panels A–C: moisture content (MC) = 25 %, and temperature (Temp) = 25, 30 and 35 °C, respectively. Panels D–F: moisture content (MC) = 50 %, and temperature (Temp) = 25, 30 and 35 °C, respectively. Panels G–I: moisture content (MC) = 75 %, and temperature (Temp) = 25, 30 and 35 °C, respectively. Colored lines indicate model-predicted values at different C/N ratios (10–70), while points represent observed mean colony counts (log₁₀ (CFU + 0.01)) with error bars showing standard error.
