# Supplementary material for: Interaction effects of physicochemical factors on the growth of Burkholderia pseudomallei in soil microcosms
Source: PLoS Negl Trop Dis. 2026 May 18;20(5):e0014339. doi: 10.1371/journal.pntd.0014339 (PMC13197065; doi:10.1371/journal.pntd.0014339)
Supplement: S1 Table — (DOCX) [file pntd.0014339.s007.docx]

**S1 Table.** Comparison of Akaike Information Criterion (AIC) values and pairwise analysis of variance (ANOVA) results among the reduced cubic interaction model (final model), the full cubic interaction model, the quadratic interaction model, and the linear interaction model.

| **Model** | **AIC** |
| --- | --- |
| **1) pH and salinity** |  |
| Complete cubic interaction model | 1986.773 |
| Reduced cubic interaction model (Final model) | 1977.517 |
| Quadratic interaction model | 2081.941* |
| Linear interaction model | 2706.877* |
| **2) C/N ratio and salinity** |  |
| Complete cubic interaction model | 2409.504 |
| Reduced cubic interaction model (Final model) | 2407.485 |
| Quadratic interaction model | 2469.238* |
| Linear interaction model | 2491.536* |
| **3) Iron contents and salinity** |  |
| Complete cubic interaction model | 3131.020 |
| Reduced cubic interaction model (Final model) | 3123.453 |
| Quadratic interaction model | 3134.906* |
| Linear interaction model | 3186.980* |

*****Asterisks indicate statistical significance at α = 0.05 from final models
