# Supplementary material for: Interaction effects of physicochemical factors on the growth of Burkholderia pseudomallei in soil microcosms
Source: PLoS Negl Trop Dis. 2026 May 18;20(5):e0014339. doi: 10.1371/journal.pntd.0014339 (PMC13197065; doi:10.1371/journal.pntd.0014339)
Supplement: S2 Table — (DOCX) [file pntd.0014339.s008.docx]

**Table S2.** Number of *B. pseudomallei* colonies on day 7 (N, CFU/mL) in three replicates under varying pH and salinity conditions at different soil temperatures and moisture contents, with C/N ratio and iron content kept constant (Salt: salinity of soil, Temp: temperature of soil, MC: moisture content of soil, C/N ratio: carbon to nitrogen ratio of soil, FE: iron content of soil).

| **Replicate** | **pH** | **Salt (%)** | **Temp (°C)** | **MC (%)** | **FE (mg/Kg)** | **C/N ratio** | **N (CFU/ml)** |
| --- | --- | --- | --- | --- | --- | --- | --- |
| 1 | 4 | 0 | 25 | 25 | 48 | 50.8982 | 2.60E+05 |
| 2 | 4 | 0 | 25 | 25 | 48 | 50.8982 | 1.50E+05 |
| 3 | 4 | 0 | 25 | 25 | 48 | 50.8982 | 1.93E+05 |
| 1 | 5 | 0 | 25 | 25 | 48 | 50.8982 | 5.20E+06 |
| 2 | 5 | 0 | 25 | 25 | 48 | 50.8982 | 5.03E+06 |
| 3 | 5 | 0 | 25 | 25 | 48 | 50.8982 | 4.77E+06 |
| 1 | 6 | 0 | 25 | 25 | 48 | 50.8982 | 4.30E+06 |
| 2 | 6 | 0 | 25 | 25 | 48 | 50.8982 | 5.00E+06 |
| 3 | 6 | 0 | 25 | 25 | 48 | 50.8982 | 5.67E+06 |
| 1 | 7 | 0 | 25 | 25 | 48 | 50.8982 | 6.30E+02 |
| 2 | 7 | 0 | 25 | 25 | 48 | 50.8982 | 5.30E+02 |
| 3 | 7 | 0 | 25 | 25 | 48 | 50.8982 | 6.40E+02 |
| 1 | 8 | 0 | 25 | 25 | 48 | 50.8982 | 1.60E+02 |
| 2 | 8 | 0 | 25 | 25 | 48 | 50.8982 | 1.20E+02 |
| 3 | 8 | 0 | 25 | 25 | 48 | 50.8982 | 2.00E+02 |
| 1 | 4 | 0.3 | 25 | 25 | 48 | 50.8982 | 2.61E+06 |
| 2 | 4 | 0.3 | 25 | 25 | 48 | 50.8982 | 3.00E+06 |
| 3 | 4 | 0.3 | 25 | 25 | 48 | 50.8982 | 2.00E+06 |
| 1 | 5 | 0.3 | 25 | 25 | 48 | 50.8982 | 1.80E+06 |
| 2 | 5 | 0.3 | 25 | 25 | 48 | 50.8982 | 2.20E+06 |
| 3 | 5 | 0.3 | 25 | 25 | 48 | 50.8982 | 2.00E+06 |
| 1 | 6 | 0.3 | 25 | 25 | 48 | 50.8982 | 2.00E+06 |
| 2 | 6 | 0.3 | 25 | 25 | 48 | 50.8982 | 1.83E+06 |
| 3 | 6 | 0.3 | 25 | 25 | 48 | 50.8982 | 2.30E+06 |
| 1 | 7 | 0.3 | 25 | 25 | 48 | 50.8982 | 2.40E+05 |
| 2 | 7 | 0.3 | 25 | 25 | 48 | 50.8982 | 3.50E+05 |
| 3 | 7 | 0.3 | 25 | 25 | 48 | 50.8982 | 3.43E+05 |
| 1 | 8 | 0.3 | 25 | 25 | 48 | 50.8982 | 3.00E+02 |
| 2 | 8 | 0.3 | 25 | 25 | 48 | 50.8982 | 3.30E+02 |
| 3 | 8 | 0.3 | 25 | 25 | 48 | 50.8982 | 3.88E+02 |
| 1 | 4 | 0.6 | 25 | 25 | 48 | 50.8982 | 3.70E+06 |
| 2 | 4 | 0.6 | 25 | 25 | 48 | 50.8982 | 4.70E+06 |
| 3 | 4 | 0.6 | 25 | 25 | 48 | 50.8982 | 4.20E+06 |
| 1 | 5 | 0.6 | 25 | 25 | 48 | 50.8982 | 7.00E+06 |
| 2 | 5 | 0.6 | 25 | 25 | 48 | 50.8982 | 6.49E+06 |
| 3 | 5 | 0.6 | 25 | 25 | 48 | 50.8982 | 7.50E+06 |
| 1 | 6 | 0.6 | 25 | 25 | 48 | 50.8982 | 7.26E+06 |
| 2 | 6 | 0.6 | 25 | 25 | 48 | 50.8982 | 7.50E+06 |
| 3 | 6 | 0.6 | 25 | 25 | 48 | 50.8982 | 6.70E+06 |
| 1 | 7 | 0.6 | 25 | 25 | 48 | 50.8982 | 3.80E+05 |
| 2 | 7 | 0.6 | 25 | 25 | 48 | 50.8982 | 3.90E+05 |
| 3 | 7 | 0.6 | 25 | 25 | 48 | 50.8982 | 4.10E+05 |
| 1 | 8 | 0.6 | 25 | 25 | 48 | 50.8982 | 3.80E+02 |
| 2 | 8 | 0.6 | 25 | 25 | 48 | 50.8982 | 4.20E+02 |
| 3 | 8 | 0.6 | 25 | 25 | 48 | 50.8982 | 3.63E+02 |
| 1 | 4 | 0.9 | 25 | 25 | 48 | 50.8982 | 4.27E+06 |
| 2 | 4 | 0.9 | 25 | 25 | 48 | 50.8982 | 5.90E+06 |
| 3 | 4 | 0.9 | 25 | 25 | 48 | 50.8982 | 4.00E+06 |
| 1 | 5 | 0.9 | 25 | 25 | 48 | 50.8982 | 5.73E+06 |
| 2 | 5 | 0.9 | 25 | 25 | 48 | 50.8982 | 5.50E+06 |
| 3 | 5 | 0.9 | 25 | 25 | 48 | 50.8982 | 6.50E+06 |
| 1 | 6 | 0.9 | 25 | 25 | 48 | 50.8982 | 4.80E+05 |
| 2 | 6 | 0.9 | 25 | 25 | 48 | 50.8982 | 5.20E+05 |
| 3 | 6 | 0.9 | 25 | 25 | 48 | 50.8982 | 4.33E+05 |
| 1 | 7 | 0.9 | 25 | 25 | 48 | 50.8982 | 3.30E+02 |
| 2 | 7 | 0.9 | 25 | 25 | 48 | 50.8982 | 2.47E+02 |
| 3 | 7 | 0.9 | 25 | 25 | 48 | 50.8982 | 2.30E+02 |
| 1 | 8 | 0.9 | 25 | 25 | 48 | 50.8982 | 0.00E+00 |
| 2 | 8 | 0.9 | 25 | 25 | 48 | 50.8982 | 0.00E+00 |
| 3 | 8 | 0.9 | 25 | 25 | 48 | 50.8982 | 0.00E+00 |
| 1 | 4 | 1.2 | 25 | 25 | 48 | 50.8982 | 0.00E+00 |
| 2 | 4 | 1.2 | 25 | 25 | 48 | 50.8982 | 0.00E+00 |
| 3 | 4 | 1.2 | 25 | 25 | 48 | 50.8982 | 0.00E+00 |
| 1 | 5 | 1.2 | 25 | 25 | 48 | 50.8982 | 1.55E+01 |
| 2 | 5 | 1.2 | 25 | 25 | 48 | 50.8982 | 1.35E+01 |
| 3 | 5 | 1.2 | 25 | 25 | 48 | 50.8982 | 1.60E+01 |
| 1 | 6 | 1.2 | 25 | 25 | 48 | 50.8982 | 1.80E+01 |
| 2 | 6 | 1.2 | 25 | 25 | 48 | 50.8982 | 1.30E+01 |
| 3 | 6 | 1.2 | 25 | 25 | 48 | 50.8982 | 2.00E+01 |
| 1 | 7 | 1.2 | 25 | 25 | 48 | 50.8982 | 0.00E+00 |
| 2 | 7 | 1.2 | 25 | 25 | 48 | 50.8982 | 0.00E+00 |
| 3 | 7 | 1.2 | 25 | 25 | 48 | 50.8982 | 0.00E+00 |
| 1 | 8 | 1.2 | 25 | 25 | 48 | 50.8982 | 0.00E+00 |
| 2 | 8 | 1.2 | 25 | 25 | 48 | 50.8982 | 0.00E+00 |
| 3 | 8 | 1.2 | 25 | 25 | 48 | 50.8982 | 0.00E+00 |
| 1 | 4 | 0 | 30 | 25 | 48 | 50.8982 | 1.07E+06 |
| 2 | 4 | 0 | 30 | 25 | 48 | 50.8982 | 9.22E+05 |
| 3 | 4 | 0 | 30 | 25 | 48 | 50.8982 | 9.70E+05 |
| 1 | 5 | 0 | 30 | 25 | 48 | 50.8982 | 2.50E+06 |
| 2 | 5 | 0 | 30 | 25 | 48 | 50.8982 | 2.47E+06 |
| 3 | 5 | 0 | 30 | 25 | 48 | 50.8982 | 3.30E+06 |
| 1 | 6 | 0 | 30 | 25 | 48 | 50.8982 | 3.28E+06 |
| 2 | 6 | 0 | 30 | 25 | 48 | 50.8982 | 3.44E+06 |
| 3 | 6 | 0 | 30 | 25 | 48 | 50.8982 | 2.70E+06 |
| 1 | 7 | 0 | 30 | 25 | 48 | 50.8982 | 5.30E+05 |
| 2 | 7 | 0 | 30 | 25 | 48 | 50.8982 | 5.20E+05 |
| 3 | 7 | 0 | 30 | 25 | 48 | 50.8982 | 4.76E+05 |
| 1 | 8 | 0 | 30 | 25 | 48 | 50.8982 | 2.50E+03 |
| 2 | 8 | 0 | 30 | 25 | 48 | 50.8982 | 3.00E+03 |
| 3 | 8 | 0 | 30 | 25 | 48 | 50.8982 | 2.30E+03 |
| 1 | 4 | 0.3 | 30 | 25 | 48 | 50.8982 | 3.30E+07 |
| 2 | 4 | 0.3 | 30 | 25 | 48 | 50.8982 | 1.37E+07 |
| 3 | 4 | 0.3 | 30 | 25 | 48 | 50.8982 | 1.70E+07 |
| 1 | 5 | 0.3 | 30 | 25 | 48 | 50.8982 | 2.36E+07 |
| 2 | 5 | 0.3 | 30 | 25 | 48 | 50.8982 | 2.47E+07 |
| 3 | 5 | 0.3 | 30 | 25 | 48 | 50.8982 | 2.40E+07 |
| 1 | 6 | 0.3 | 30 | 25 | 48 | 50.8982 | 1.17E+07 |
| 2 | 6 | 0.3 | 30 | 25 | 48 | 50.8982 | 2.76E+07 |
| 3 | 6 | 0.3 | 30 | 25 | 48 | 50.8982 | 2.23E+07 |
| 1 | 7 | 0.3 | 30 | 25 | 48 | 50.8982 | 7.10E+05 |
| 2 | 7 | 0.3 | 30 | 25 | 48 | 50.8982 | 6.97E+05 |
| 3 | 7 | 0.3 | 30 | 25 | 48 | 50.8982 | 7.44E+05 |
| 1 | 8 | 0.3 | 30 | 25 | 48 | 50.8982 | 1.37E+03 |
| 2 | 8 | 0.3 | 30 | 25 | 48 | 50.8982 | 1.40E+03 |
| 3 | 8 | 0.3 | 30 | 25 | 48 | 50.8982 | 1.50E+03 |
| 1 | 4 | 0.6 | 30 | 25 | 48 | 50.8982 | 7.30E+06 |
| 2 | 4 | 0.6 | 30 | 25 | 48 | 50.8982 | 7.30E+06 |
| 3 | 4 | 0.6 | 30 | 25 | 48 | 50.8982 | 7.40E+06 |
| 1 | 5 | 0.6 | 30 | 25 | 48 | 50.8982 | 4.20E+07 |
| 2 | 5 | 0.6 | 30 | 25 | 48 | 50.8982 | 5.40E+07 |
| 3 | 5 | 0.6 | 30 | 25 | 48 | 50.8982 | 5.39E+07 |
| 1 | 6 | 0.6 | 30 | 25 | 48 | 50.8982 | 2.14E+07 |
| 2 | 6 | 0.6 | 30 | 25 | 48 | 50.8982 | 2.00E+07 |
| 3 | 6 | 0.6 | 30 | 25 | 48 | 50.8982 | 2.20E+07 |
| 1 | 7 | 0.6 | 30 | 25 | 48 | 50.8982 | 3.39E+05 |
| 2 | 7 | 0.6 | 30 | 25 | 48 | 50.8982 | 4.00E+05 |
| 3 | 7 | 0.6 | 30 | 25 | 48 | 50.8982 | 3.40E+05 |
| 1 | 8 | 0.6 | 30 | 25 | 48 | 50.8982 | 5.60E+02 |
| 2 | 8 | 0.6 | 30 | 25 | 48 | 50.8982 | 6.30E+02 |
| 3 | 8 | 0.6 | 30 | 25 | 48 | 50.8982 | 5.50E+02 |
| 1 | 4 | 0.9 | 30 | 25 | 48 | 50.8982 | 5.30E+04 |
| 2 | 4 | 0.9 | 30 | 25 | 48 | 50.8982 | 5.55E+04 |
| 3 | 4 | 0.9 | 30 | 25 | 48 | 50.8982 | 5.06E+04 |
| 1 | 5 | 0.9 | 30 | 25 | 48 | 50.8982 | 5.28E+05 |
| 2 | 5 | 0.9 | 30 | 25 | 48 | 50.8982 | 5.50E+05 |
| 3 | 5 | 0.9 | 30 | 25 | 48 | 50.8982 | 4.63E+05 |
| 1 | 6 | 0.9 | 30 | 25 | 48 | 50.8982 | 4.75E+05 |
| 2 | 6 | 0.9 | 30 | 25 | 48 | 50.8982 | 5.14E+05 |
| 3 | 6 | 0.9 | 30 | 25 | 48 | 50.8982 | 3.70E+05 |
| 1 | 7 | 0.9 | 30 | 25 | 48 | 50.8982 | 1.28E+03 |
| 2 | 7 | 0.9 | 30 | 25 | 48 | 50.8982 | 1.12E+03 |
| 3 | 7 | 0.9 | 30 | 25 | 48 | 50.8982 | 1.53E+03 |
| 1 | 8 | 0.9 | 30 | 25 | 48 | 50.8982 | 0.00E+00 |
| 2 | 8 | 0.9 | 30 | 25 | 48 | 50.8982 | 0.00E+00 |
| 3 | 8 | 0.9 | 30 | 25 | 48 | 50.8982 | 0.00E+00 |
| 1 | 4 | 1.2 | 30 | 25 | 48 | 50.8982 | 1.52E+01 |
| 2 | 4 | 1.2 | 30 | 25 | 48 | 50.8982 | 1.70E+01 |
| 3 | 4 | 1.2 | 30 | 25 | 48 | 50.8982 | 1.58E+01 |
| 1 | 5 | 1.2 | 30 | 25 | 48 | 50.8982 | 5.00E+01 |
| 2 | 5 | 1.2 | 30 | 25 | 48 | 50.8982 | 4.40E+01 |
| 3 | 5 | 1.2 | 30 | 25 | 48 | 50.8982 | 8.40E+01 |
| 1 | 6 | 1.2 | 30 | 25 | 48 | 50.8982 | 8.90E+01 |
| 2 | 6 | 1.2 | 30 | 25 | 48 | 50.8982 | 4.14E+01 |
| 3 | 6 | 1.2 | 30 | 25 | 48 | 50.8982 | 4.35E+01 |
| 1 | 7 | 1.2 | 30 | 25 | 48 | 50.8982 | 0.00E+00 |
| 2 | 7 | 1.2 | 30 | 25 | 48 | 50.8982 | 0.00E+00 |
| 3 | 7 | 1.2 | 30 | 25 | 48 | 50.8982 | 0.00E+00 |
| 1 | 8 | 1.2 | 30 | 25 | 48 | 50.8982 | 0.00E+00 |
| 2 | 8 | 1.2 | 30 | 25 | 48 | 50.8982 | 0.00E+00 |
| 3 | 8 | 1.2 | 30 | 25 | 48 | 50.8982 | 0.00E+00 |
| 1 | 4 | 0 | 35 | 25 | 48 | 50.8982 | 4.45E+07 |
| 2 | 4 | 0 | 35 | 25 | 48 | 50.8982 | 4.30E+07 |
| 3 | 4 | 0 | 35 | 25 | 48 | 50.8982 | 3.60E+07 |
| 1 | 5 | 0 | 35 | 25 | 48 | 50.8982 | 3.00E+07 |
| 2 | 5 | 0 | 35 | 25 | 48 | 50.8982 | 2.30E+07 |
| 3 | 5 | 0 | 35 | 25 | 48 | 50.8982 | 2.50E+07 |
| 1 | 6 | 0 | 35 | 25 | 48 | 50.8982 | 3.00E+06 |
| 2 | 6 | 0 | 35 | 25 | 48 | 50.8982 | 3.23E+06 |
| 3 | 6 | 0 | 35 | 25 | 48 | 50.8982 | 3.20E+06 |
| 1 | 7 | 0 | 35 | 25 | 48 | 50.8982 | 4.80E+04 |
| 2 | 7 | 0 | 35 | 25 | 48 | 50.8982 | 5.08E+04 |
| 3 | 7 | 0 | 35 | 25 | 48 | 50.8982 | 5.40E+04 |
| 1 | 8 | 0 | 35 | 25 | 48 | 50.8982 | 5.80E+03 |
| 2 | 8 | 0 | 35 | 25 | 48 | 50.8982 | 3.83E+03 |
| 3 | 8 | 0 | 35 | 25 | 48 | 50.8982 | 3.60E+03 |
| 1 | 4 | 0.3 | 35 | 25 | 48 | 50.8982 | 3.17E+08 |
| 2 | 4 | 0.3 | 35 | 25 | 48 | 50.8982 | 3.20E+08 |
| 3 | 4 | 0.3 | 35 | 25 | 48 | 50.8982 | 3.80E+08 |
| 1 | 5 | 0.3 | 35 | 25 | 48 | 50.8982 | 4.00E+08 |
| 2 | 5 | 0.3 | 35 | 25 | 48 | 50.8982 | 4.78E+08 |
| 3 | 5 | 0.3 | 35 | 25 | 48 | 50.8982 | 4.50E+08 |
| 1 | 6 | 0.3 | 35 | 25 | 48 | 50.8982 | 5.70E+08 |
| 2 | 6 | 0.3 | 35 | 25 | 48 | 50.8982 | 5.27E+08 |
| 3 | 6 | 0.3 | 35 | 25 | 48 | 50.8982 | 6.00E+08 |
| 1 | 7 | 0.3 | 35 | 25 | 48 | 50.8982 | 8.20E+05 |
| 2 | 7 | 0.3 | 35 | 25 | 48 | 50.8982 | 7.50E+05 |
| 3 | 7 | 0.3 | 35 | 25 | 48 | 50.8982 | 7.80E+05 |
| 1 | 8 | 0.3 | 35 | 25 | 48 | 50.8982 | 1.99E+04 |
| 2 | 8 | 0.3 | 35 | 25 | 48 | 50.8982 | 1.85E+04 |
| 3 | 8 | 0.3 | 35 | 25 | 48 | 50.8982 | 2.00E+04 |
| 1 | 4 | 0.6 | 35 | 25 | 48 | 50.8982 | 2.10E+08 |
| 2 | 4 | 0.6 | 35 | 25 | 48 | 50.8982 | 1.90E+08 |
| 3 | 4 | 0.6 | 35 | 25 | 48 | 50.8982 | 2.16E+08 |
| 1 | 5 | 0.6 | 35 | 25 | 48 | 50.8982 | 5.70E+08 |
| 2 | 5 | 0.6 | 35 | 25 | 48 | 50.8982 | 5.30E+08 |
| 3 | 5 | 0.6 | 35 | 25 | 48 | 50.8982 | 5.50E+08 |
| 1 | 6 | 0.6 | 35 | 25 | 48 | 50.8982 | 1.80E+09 |
| 2 | 6 | 0.6 | 35 | 25 | 48 | 50.8982 | 1.60E+09 |
| 3 | 6 | 0.6 | 35 | 25 | 48 | 50.8982 | 1.95E+09 |
| 1 | 7 | 0.6 | 35 | 25 | 48 | 50.8982 | 3.16E+05 |
| 2 | 7 | 0.6 | 35 | 25 | 48 | 50.8982 | 3.00E+05 |
| 3 | 7 | 0.6 | 35 | 25 | 48 | 50.8982 | 3.60E+05 |
| 1 | 8 | 0.6 | 35 | 25 | 48 | 50.8982 | 2.70E+03 |
| 2 | 8 | 0.6 | 35 | 25 | 48 | 50.8982 | 3.80E+03 |
| 3 | 8 | 0.6 | 35 | 25 | 48 | 50.8982 | 3.40E+03 |
| 1 | 4 | 0.9 | 35 | 25 | 48 | 50.8982 | 1.40E+06 |
| 2 | 4 | 0.9 | 35 | 25 | 48 | 50.8982 | 3.30E+06 |
| 3 | 4 | 0.9 | 35 | 25 | 48 | 50.8982 | 1.98E+06 |
| 1 | 5 | 0.9 | 35 | 25 | 48 | 50.8982 | 4.60E+07 |
| 2 | 5 | 0.9 | 35 | 25 | 48 | 50.8982 | 5.00E+07 |
| 3 | 5 | 0.9 | 35 | 25 | 48 | 50.8982 | 4.40E+07 |
| 1 | 6 | 0.9 | 35 | 25 | 48 | 50.8982 | 6.00E+07 |
| 2 | 6 | 0.9 | 35 | 25 | 48 | 50.8982 | 7.27E+07 |
| 3 | 6 | 0.9 | 35 | 25 | 48 | 50.8982 | 6.90E+07 |
| 1 | 7 | 0.9 | 35 | 25 | 48 | 50.8982 | 5.70E+03 |
| 2 | 7 | 0.9 | 35 | 25 | 48 | 50.8982 | 6.57E+03 |
| 3 | 7 | 0.9 | 35 | 25 | 48 | 50.8982 | 6.50E+03 |
| 1 | 8 | 0.9 | 35 | 25 | 48 | 50.8982 | 6.20E+01 |
| 2 | 8 | 0.9 | 35 | 25 | 48 | 50.8982 | 6.30E+01 |
| 3 | 8 | 0.9 | 35 | 25 | 48 | 50.8982 | 6.28E+01 |
| 1 | 4 | 1.2 | 35 | 25 | 48 | 50.8982 | 2.20E+01 |
| 2 | 4 | 1.2 | 35 | 25 | 48 | 50.8982 | 2.00E+01 |
| 3 | 4 | 1.2 | 35 | 25 | 48 | 50.8982 | 1.81E+01 |
| 1 | 5 | 1.2 | 35 | 25 | 48 | 50.8982 | 3.80E+02 |
| 2 | 5 | 1.2 | 35 | 25 | 48 | 50.8982 | 4.47E+02 |
| 3 | 5 | 1.2 | 35 | 25 | 48 | 50.8982 | 4.00E+02 |
| 1 | 6 | 1.2 | 35 | 25 | 48 | 50.8982 | 1.60E+02 |
| 2 | 6 | 1.2 | 35 | 25 | 48 | 50.8982 | 1.83E+02 |
| 3 | 6 | 1.2 | 35 | 25 | 48 | 50.8982 | 2.00E+02 |
| 1 | 7 | 1.2 | 35 | 25 | 48 | 50.8982 | 0.00E+00 |
| 2 | 7 | 1.2 | 35 | 25 | 48 | 50.8982 | 0.00E+00 |
| 3 | 7 | 1.2 | 35 | 25 | 48 | 50.8982 | 0.00E+00 |
| 1 | 8 | 1.2 | 35 | 25 | 48 | 50.8982 | 0.00E+00 |
| 2 | 8 | 1.2 | 35 | 25 | 48 | 50.8982 | 0.00E+00 |
| 3 | 8 | 1.2 | 35 | 25 | 48 | 50.8982 | 0.00E+00 |
| 1 | 4 | 0 | 25 | 50 | 48 | 50.8982 | 4.80E+07 |
| 2 | 4 | 0 | 25 | 50 | 48 | 50.8982 | 4.50E+07 |
| 3 | 4 | 0 | 25 | 50 | 48 | 50.8982 | 5.40E+07 |
| 1 | 5 | 0 | 25 | 50 | 48 | 50.8982 | 7.20E+07 |
| 2 | 5 | 0 | 25 | 50 | 48 | 50.8982 | 9.60E+07 |
| 3 | 5 | 0 | 25 | 50 | 48 | 50.8982 | 5.10E+07 |
| 1 | 6 | 0 | 25 | 50 | 48 | 50.8982 | 1.17E+08 |
| 2 | 6 | 0 | 25 | 50 | 48 | 50.8982 | 1.33E+08 |
| 3 | 6 | 0 | 25 | 50 | 48 | 50.8982 | 1.45E+08 |
| 1 | 7 | 0 | 25 | 50 | 48 | 50.8982 | 4.34E+04 |
| 2 | 7 | 0 | 25 | 50 | 48 | 50.8982 | 4.30E+04 |
| 3 | 7 | 0 | 25 | 50 | 48 | 50.8982 | 5.00E+04 |
| 1 | 8 | 0 | 25 | 50 | 48 | 50.8982 | 8.90E+03 |
| 2 | 8 | 0 | 25 | 50 | 48 | 50.8982 | 8.25E+03 |
| 3 | 8 | 0 | 25 | 50 | 48 | 50.8982 | 8.50E+03 |
| 1 | 4 | 0.3 | 25 | 50 | 48 | 50.8982 | 3.40E+07 |
| 2 | 4 | 0.3 | 25 | 50 | 48 | 50.8982 | 3.30E+07 |
| 3 | 4 | 0.3 | 25 | 50 | 48 | 50.8982 | 4.10E+07 |
| 1 | 5 | 0.3 | 25 | 50 | 48 | 50.8982 | 1.15E+08 |
| 2 | 5 | 0.3 | 25 | 50 | 48 | 50.8982 | 1.27E+08 |
| 3 | 5 | 0.3 | 25 | 50 | 48 | 50.8982 | 1.20E+08 |
| 1 | 6 | 0.3 | 25 | 50 | 48 | 50.8982 | 1.98E+08 |
| 2 | 6 | 0.3 | 25 | 50 | 48 | 50.8982 | 1.82E+08 |
| 3 | 6 | 0.3 | 25 | 50 | 48 | 50.8982 | 8.70E+07 |
| 1 | 7 | 0.3 | 25 | 50 | 48 | 50.8982 | 5.70E+04 |
| 2 | 7 | 0.3 | 25 | 50 | 48 | 50.8982 | 6.25E+04 |
| 3 | 7 | 0.3 | 25 | 50 | 48 | 50.8982 | 6.80E+04 |
| 1 | 8 | 0.3 | 25 | 50 | 48 | 50.8982 | 3.10E+03 |
| 2 | 8 | 0.3 | 25 | 50 | 48 | 50.8982 | 2.70E+03 |
| 3 | 8 | 0.3 | 25 | 50 | 48 | 50.8982 | 3.40E+03 |
| 1 | 4 | 0.6 | 25 | 50 | 48 | 50.8982 | 8.80E+07 |
| 2 | 4 | 0.6 | 25 | 50 | 48 | 50.8982 | 9.40E+07 |
| 3 | 4 | 0.6 | 25 | 50 | 48 | 50.8982 | 8.65E+07 |
| 1 | 5 | 0.6 | 25 | 50 | 48 | 50.8982 | 8.45E+07 |
| 2 | 5 | 0.6 | 25 | 50 | 48 | 50.8982 | 8.13E+07 |
| 3 | 5 | 0.6 | 25 | 50 | 48 | 50.8982 | 8.70E+07 |
| 1 | 6 | 0.6 | 25 | 50 | 48 | 50.8982 | 8.00E+07 |
| 2 | 6 | 0.6 | 25 | 50 | 48 | 50.8982 | 8.47E+07 |
| 3 | 6 | 0.6 | 25 | 50 | 48 | 50.8982 | 8.50E+07 |
| 1 | 7 | 0.6 | 25 | 50 | 48 | 50.8982 | 4.70E+04 |
| 2 | 7 | 0.6 | 25 | 50 | 48 | 50.8982 | 5.50E+04 |
| 3 | 7 | 0.6 | 25 | 50 | 48 | 50.8982 | 5.27E+04 |
| 1 | 8 | 0.6 | 25 | 50 | 48 | 50.8982 | 4.60E+03 |
| 2 | 8 | 0.6 | 25 | 50 | 48 | 50.8982 | 4.20E+03 |
| 3 | 8 | 0.6 | 25 | 50 | 48 | 50.8982 | 5.00E+03 |
| 1 | 4 | 0.9 | 25 | 50 | 48 | 50.8982 | 3.70E+06 |
| 2 | 4 | 0.9 | 25 | 50 | 48 | 50.8982 | 3.00E+06 |
| 3 | 4 | 0.9 | 25 | 50 | 48 | 50.8982 | 4.70E+06 |
| 1 | 5 | 0.9 | 25 | 50 | 48 | 50.8982 | 7.33E+07 |
| 2 | 5 | 0.9 | 25 | 50 | 48 | 50.8982 | 6.90E+07 |
| 3 | 5 | 0.9 | 25 | 50 | 48 | 50.8982 | 7.14E+07 |
| 1 | 6 | 0.9 | 25 | 50 | 48 | 50.8982 | 4.30E+07 |
| 2 | 6 | 0.9 | 25 | 50 | 48 | 50.8982 | 4.70E+07 |
| 3 | 6 | 0.9 | 25 | 50 | 48 | 50.8982 | 4.20E+07 |
| 1 | 7 | 0.9 | 25 | 50 | 48 | 50.8982 | 2.00E+03 |
| 2 | 7 | 0.9 | 25 | 50 | 48 | 50.8982 | 2.46E+03 |
| 3 | 7 | 0.9 | 25 | 50 | 48 | 50.8982 | 2.30E+03 |
| 1 | 8 | 0.9 | 25 | 50 | 48 | 50.8982 | 1.87E+02 |
| 2 | 8 | 0.9 | 25 | 50 | 48 | 50.8982 | 2.13E+02 |
| 3 | 8 | 0.9 | 25 | 50 | 48 | 50.8982 | 2.00E+02 |
| 1 | 4 | 1.2 | 25 | 50 | 48 | 50.8982 | 5.30E+04 |
| 2 | 4 | 1.2 | 25 | 50 | 48 | 50.8982 | 6.00E+04 |
| 3 | 4 | 1.2 | 25 | 50 | 48 | 50.8982 | 5.20E+04 |
| 1 | 5 | 1.2 | 25 | 50 | 48 | 50.8982 | 1.40E+05 |
| 2 | 5 | 1.2 | 25 | 50 | 48 | 50.8982 | 1.53E+05 |
| 3 | 5 | 1.2 | 25 | 50 | 48 | 50.8982 | 2.00E+05 |
| 1 | 6 | 1.2 | 25 | 50 | 48 | 50.8982 | 1.30E+05 |
| 2 | 6 | 1.2 | 25 | 50 | 48 | 50.8982 | 1.43E+05 |
| 3 | 6 | 1.2 | 25 | 50 | 48 | 50.8982 | 7.55E+04 |
| 1 | 7 | 1.2 | 25 | 50 | 48 | 50.8982 | 2.70E+02 |
| 2 | 7 | 1.2 | 25 | 50 | 48 | 50.8982 | 2.28E+02 |
| 3 | 7 | 1.2 | 25 | 50 | 48 | 50.8982 | 2.60E+02 |
| 1 | 8 | 1.2 | 25 | 50 | 48 | 50.8982 | 2.20E+01 |
| 2 | 8 | 1.2 | 25 | 50 | 48 | 50.8982 | 1.60E+01 |
| 3 | 8 | 1.2 | 25 | 50 | 48 | 50.8982 | 1.70E+01 |
| 1 | 4 | 0 | 30 | 50 | 48 | 50.8982 | 7.90E+07 |
| 2 | 4 | 0 | 30 | 50 | 48 | 50.8982 | 7.85E+07 |
| 3 | 4 | 0 | 30 | 50 | 48 | 50.8982 | 8.10E+07 |
| 1 | 5 | 0 | 30 | 50 | 48 | 50.8982 | 9.70E+08 |
| 2 | 5 | 0 | 30 | 50 | 48 | 50.8982 | 9.50E+08 |
| 3 | 5 | 0 | 30 | 50 | 48 | 50.8982 | 9.80E+08 |
| 1 | 6 | 0 | 30 | 50 | 48 | 50.8982 | 7.40E+09 |
| 2 | 6 | 0 | 30 | 50 | 48 | 50.8982 | 7.50E+09 |
| 3 | 6 | 0 | 30 | 50 | 48 | 50.8982 | 8.00E+09 |
| 1 | 7 | 0 | 30 | 50 | 48 | 50.8982 | 4.80E+06 |
| 2 | 7 | 0 | 30 | 50 | 48 | 50.8982 | 4.20E+06 |
| 3 | 7 | 0 | 30 | 50 | 48 | 50.8982 | 4.76E+06 |
| 1 | 8 | 0 | 30 | 50 | 48 | 50.8982 | 8.53E+04 |
| 2 | 8 | 0 | 30 | 50 | 48 | 50.8982 | 8.40E+04 |
| 3 | 8 | 0 | 30 | 50 | 48 | 50.8982 | 8.30E+04 |
| 1 | 4 | 0.3 | 30 | 50 | 48 | 50.8982 | 9.80E+08 |
| 2 | 4 | 0.3 | 30 | 50 | 48 | 50.8982 | 9.50E+08 |
| 3 | 4 | 0.3 | 30 | 50 | 48 | 50.8982 | 9.45E+08 |
| 1 | 5 | 0.3 | 30 | 50 | 48 | 50.8982 | 2.60E+09 |
| 2 | 5 | 0.3 | 30 | 50 | 48 | 50.8982 | 2.14E+09 |
| 3 | 5 | 0.3 | 30 | 50 | 48 | 50.8982 | 2.20E+09 |
| 1 | 6 | 0.3 | 30 | 50 | 48 | 50.8982 | 1.30E+09 |
| 2 | 6 | 0.3 | 30 | 50 | 48 | 50.8982 | 2.11E+09 |
| 3 | 6 | 0.3 | 30 | 50 | 48 | 50.8982 | 2.13E+09 |
| 1 | 7 | 0.3 | 30 | 50 | 48 | 50.8982 | 7.63E+05 |
| 2 | 7 | 0.3 | 30 | 50 | 48 | 50.8982 | 8.00E+05 |
| 3 | 7 | 0.3 | 30 | 50 | 48 | 50.8982 | 7.60E+05 |
| 1 | 8 | 0.3 | 30 | 50 | 48 | 50.8982 | 1.19E+04 |
| 2 | 8 | 0.3 | 30 | 50 | 48 | 50.8982 | 1.09E+04 |
| 3 | 8 | 0.3 | 30 | 50 | 48 | 50.8982 | 1.10E+04 |
| 1 | 4 | 0.6 | 30 | 50 | 48 | 50.8982 | 6.10E+08 |
| 2 | 4 | 0.6 | 30 | 50 | 48 | 50.8982 | 6.30E+08 |
| 3 | 4 | 0.6 | 30 | 50 | 48 | 50.8982 | 6.25E+08 |
| 1 | 5 | 0.6 | 30 | 50 | 48 | 50.8982 | 7.07E+08 |
| 2 | 5 | 0.6 | 30 | 50 | 48 | 50.8982 | 7.50E+08 |
| 3 | 5 | 0.6 | 30 | 50 | 48 | 50.8982 | 7.97E+08 |
| 1 | 6 | 0.6 | 30 | 50 | 48 | 50.8982 | 7.80E+09 |
| 2 | 6 | 0.6 | 30 | 50 | 48 | 50.8982 | 8.00E+09 |
| 3 | 6 | 0.6 | 30 | 50 | 48 | 50.8982 | 7.65E+09 |
| 1 | 7 | 0.6 | 30 | 50 | 48 | 50.8982 | 8.20E+04 |
| 2 | 7 | 0.6 | 30 | 50 | 48 | 50.8982 | 7.70E+04 |
| 3 | 7 | 0.6 | 30 | 50 | 48 | 50.8982 | 7.30E+04 |
| 1 | 8 | 0.6 | 30 | 50 | 48 | 50.8982 | 6.35E+04 |
| 2 | 8 | 0.6 | 30 | 50 | 48 | 50.8982 | 5.80E+04 |
| 3 | 8 | 0.6 | 30 | 50 | 48 | 50.8982 | 5.70E+04 |
| 1 | 4 | 0.9 | 30 | 50 | 48 | 50.8982 | 1.20E+08 |
| 2 | 4 | 0.9 | 30 | 50 | 48 | 50.8982 | 1.50E+08 |
| 3 | 4 | 0.9 | 30 | 50 | 48 | 50.8982 | 1.15E+08 |
| 1 | 5 | 0.9 | 30 | 50 | 48 | 50.8982 | 6.60E+08 |
| 2 | 5 | 0.9 | 30 | 50 | 48 | 50.8982 | 7.00E+08 |
| 3 | 5 | 0.9 | 30 | 50 | 48 | 50.8982 | 6.17E+08 |
| 1 | 6 | 0.9 | 30 | 50 | 48 | 50.8982 | 5.85E+08 |
| 2 | 6 | 0.9 | 30 | 50 | 48 | 50.8982 | 6.14E+08 |
| 3 | 6 | 0.9 | 30 | 50 | 48 | 50.8982 | 5.70E+08 |
| 1 | 7 | 0.9 | 30 | 50 | 48 | 50.8982 | 3.96E+04 |
| 2 | 7 | 0.9 | 30 | 50 | 48 | 50.8982 | 4.00E+04 |
| 3 | 7 | 0.9 | 30 | 50 | 48 | 50.8982 | 4.30E+04 |
| 1 | 8 | 0.9 | 30 | 50 | 48 | 50.8982 | 3.50E+02 |
| 2 | 8 | 0.9 | 30 | 50 | 48 | 50.8982 | 3.60E+02 |
| 3 | 8 | 0.9 | 30 | 50 | 48 | 50.8982 | 3.00E+02 |
| 1 | 4 | 1.2 | 30 | 50 | 48 | 50.8982 | 2.17E+06 |
| 2 | 4 | 1.2 | 30 | 50 | 48 | 50.8982 | 2.60E+06 |
| 3 | 4 | 1.2 | 30 | 50 | 48 | 50.8982 | 2.20E+06 |
| 1 | 5 | 1.2 | 30 | 50 | 48 | 50.8982 | 8.00E+06 |
| 2 | 5 | 1.2 | 30 | 50 | 48 | 50.8982 | 7.50E+06 |
| 3 | 5 | 1.2 | 30 | 50 | 48 | 50.8982 | 7.42E+06 |
| 1 | 6 | 1.2 | 30 | 50 | 48 | 50.8982 | 9.35E+06 |
| 2 | 6 | 1.2 | 30 | 50 | 48 | 50.8982 | 8.90E+06 |
| 3 | 6 | 1.2 | 30 | 50 | 48 | 50.8982 | 9.20E+06 |
| 1 | 7 | 1.2 | 30 | 50 | 48 | 50.8982 | 4.70E+02 |
| 2 | 7 | 1.2 | 30 | 50 | 48 | 50.8982 | 5.00E+02 |
| 3 | 7 | 1.2 | 30 | 50 | 48 | 50.8982 | 5.30E+02 |
| 1 | 8 | 1.2 | 30 | 50 | 48 | 50.8982 | 3.13E+01 |
| 2 | 8 | 1.2 | 30 | 50 | 48 | 50.8982 | 2.20E+01 |
| 3 | 8 | 1.2 | 30 | 50 | 48 | 50.8982 | 2.43E+01 |
| 1 | 4 | 0 | 35 | 50 | 48 | 50.8982 | 5.10E+07 |
| 2 | 4 | 0 | 35 | 50 | 48 | 50.8982 | 5.07E+07 |
| 3 | 4 | 0 | 35 | 50 | 48 | 50.8982 | 5.15E+07 |
| 1 | 5 | 0 | 35 | 50 | 48 | 50.8982 | 4.13E+09 |
| 2 | 5 | 0 | 35 | 50 | 48 | 50.8982 | 4.30E+09 |
| 3 | 5 | 0 | 35 | 50 | 48 | 50.8982 | 4.33E+09 |
| 1 | 6 | 0 | 35 | 50 | 48 | 50.8982 | 8.00E+09 |
| 2 | 6 | 0 | 35 | 50 | 48 | 50.8982 | 7.30E+09 |
| 3 | 6 | 0 | 35 | 50 | 48 | 50.8982 | 7.60E+09 |
| 1 | 7 | 0 | 35 | 50 | 48 | 50.8982 | 4.48E+09 |
| 2 | 7 | 0 | 35 | 50 | 48 | 50.8982 | 5.00E+09 |
| 3 | 7 | 0 | 35 | 50 | 48 | 50.8982 | 4.30E+09 |
| 1 | 8 | 0 | 35 | 50 | 48 | 50.8982 | 2.24E+05 |
| 2 | 8 | 0 | 35 | 50 | 48 | 50.8982 | 2.70E+05 |
| 3 | 8 | 0 | 35 | 50 | 48 | 50.8982 | 2.30E+05 |
| 1 | 4 | 0.3 | 35 | 50 | 48 | 50.8982 | 3.37E+09 |
| 2 | 4 | 0.3 | 35 | 50 | 48 | 50.8982 | 3.20E+09 |
| 3 | 4 | 0.3 | 35 | 50 | 48 | 50.8982 | 3.60E+09 |
| 1 | 5 | 0.3 | 35 | 50 | 48 | 50.8982 | 7.15E+10 |
| 2 | 5 | 0.3 | 35 | 50 | 48 | 50.8982 | 6.20E+10 |
| 3 | 5 | 0.3 | 35 | 50 | 48 | 50.8982 | 6.59E+10 |
| 1 | 6 | 0.3 | 35 | 50 | 48 | 50.8982 | 6.40E+10 |
| 2 | 6 | 0.3 | 35 | 50 | 48 | 50.8982 | 6.70E+10 |
| 3 | 6 | 0.3 | 35 | 50 | 48 | 50.8982 | 6.84E+10 |
| 1 | 7 | 0.3 | 35 | 50 | 48 | 50.8982 | 1.93E+06 |
| 2 | 7 | 0.3 | 35 | 50 | 48 | 50.8982 | 1.80E+06 |
| 3 | 7 | 0.3 | 35 | 50 | 48 | 50.8982 | 1.50E+06 |
| 1 | 8 | 0.3 | 35 | 50 | 48 | 50.8982 | 1.50E+05 |
| 2 | 8 | 0.3 | 35 | 50 | 48 | 50.8982 | 1.48E+05 |
| 3 | 8 | 0.3 | 35 | 50 | 48 | 50.8982 | 1.60E+05 |
| 1 | 4 | 0.6 | 35 | 50 | 48 | 50.8982 | 6.00E+09 |
| 2 | 4 | 0.6 | 35 | 50 | 48 | 50.8982 | 6.60E+09 |
| 3 | 4 | 0.6 | 35 | 50 | 48 | 50.8982 | 6.38E+09 |
| 1 | 5 | 0.6 | 35 | 50 | 48 | 50.8982 | 3.57E+10 |
| 2 | 5 | 0.6 | 35 | 50 | 48 | 50.8982 | 3.60E+10 |
| 3 | 5 | 0.6 | 35 | 50 | 48 | 50.8982 | 3.70E+10 |
| 1 | 6 | 0.6 | 35 | 50 | 48 | 50.8982 | 8.50E+10 |
| 2 | 6 | 0.6 | 35 | 50 | 48 | 50.8982 | 7.80E+10 |
| 3 | 6 | 0.6 | 35 | 50 | 48 | 50.8982 | 8.30E+10 |
| 1 | 7 | 0.6 | 35 | 50 | 48 | 50.8982 | 7.80E+05 |
| 2 | 7 | 0.6 | 35 | 50 | 48 | 50.8982 | 8.30E+05 |
| 3 | 7 | 0.6 | 35 | 50 | 48 | 50.8982 | 7.90E+05 |
| 1 | 8 | 0.6 | 35 | 50 | 48 | 50.8982 | 5.70E+03 |
| 2 | 8 | 0.6 | 35 | 50 | 48 | 50.8982 | 5.48E+03 |
| 3 | 8 | 0.6 | 35 | 50 | 48 | 50.8982 | 5.30E+03 |
| 1 | 4 | 0.9 | 35 | 50 | 48 | 50.8982 | 4.70E+08 |
| 2 | 4 | 0.9 | 35 | 50 | 48 | 50.8982 | 4.88E+08 |
| 3 | 4 | 0.9 | 35 | 50 | 48 | 50.8982 | 5.00E+08 |
| 1 | 5 | 0.9 | 35 | 50 | 48 | 50.8982 | 9.30E+09 |
| 2 | 5 | 0.9 | 35 | 50 | 48 | 50.8982 | 9.20E+09 |
| 3 | 5 | 0.9 | 35 | 50 | 48 | 50.8982 | 9.10E+09 |
| 1 | 6 | 0.9 | 35 | 50 | 48 | 50.8982 | 7.80E+09 |
| 2 | 6 | 0.9 | 35 | 50 | 48 | 50.8982 | 7.60E+09 |
| 3 | 6 | 0.9 | 35 | 50 | 48 | 50.8982 | 8.16E+09 |
| 1 | 7 | 0.9 | 35 | 50 | 48 | 50.8982 | 5.83E+04 |
| 2 | 7 | 0.9 | 35 | 50 | 48 | 50.8982 | 5.88E+04 |
| 3 | 7 | 0.9 | 35 | 50 | 48 | 50.8982 | 6.20E+04 |
| 1 | 8 | 0.9 | 35 | 50 | 48 | 50.8982 | 1.70E+03 |
| 2 | 8 | 0.9 | 35 | 50 | 48 | 50.8982 | 1.47E+03 |
| 3 | 8 | 0.9 | 35 | 50 | 48 | 50.8982 | 1.50E+03 |
| 1 | 4 | 1.2 | 35 | 50 | 48 | 50.8982 | 1.75E+06 |
| 2 | 4 | 1.2 | 35 | 50 | 48 | 50.8982 | 1.89E+06 |
| 3 | 4 | 1.2 | 35 | 50 | 48 | 50.8982 | 1.90E+06 |
| 1 | 5 | 1.2 | 35 | 50 | 48 | 50.8982 | 3.90E+08 |
| 2 | 5 | 1.2 | 35 | 50 | 48 | 50.8982 | 3.84E+08 |
| 3 | 5 | 1.2 | 35 | 50 | 48 | 50.8982 | 4.07E+08 |
| 1 | 6 | 1.2 | 35 | 50 | 48 | 50.8982 | 6.20E+08 |
| 2 | 6 | 1.2 | 35 | 50 | 48 | 50.8982 | 7.30E+08 |
| 3 | 6 | 1.2 | 35 | 50 | 48 | 50.8982 | 6.60E+08 |
| 1 | 7 | 1.2 | 35 | 50 | 48 | 50.8982 | 6.33E+02 |
| 2 | 7 | 1.2 | 35 | 50 | 48 | 50.8982 | 5.50E+02 |
| 3 | 7 | 1.2 | 35 | 50 | 48 | 50.8982 | 6.70E+02 |
| 1 | 8 | 1.2 | 35 | 50 | 48 | 50.8982 | 6.88E+01 |
| 2 | 8 | 1.2 | 35 | 50 | 48 | 50.8982 | 6.57E+01 |
| 3 | 8 | 1.2 | 35 | 50 | 48 | 50.8982 | 6.50E+01 |
| 1 | 4 | 0 | 25 | 75 | 48 | 50.8982 | 5.20E+07 |
| 2 | 4 | 0 | 25 | 75 | 48 | 50.8982 | 5.60E+07 |
| 3 | 4 | 0 | 25 | 75 | 48 | 50.8982 | 5.31E+07 |
| 1 | 5 | 0 | 25 | 75 | 48 | 50.8982 | 7.19E+07 |
| 2 | 5 | 0 | 25 | 75 | 48 | 50.8982 | 7.20E+07 |
| 3 | 5 | 0 | 25 | 75 | 48 | 50.8982 | 7.50E+07 |
| 1 | 6 | 0 | 25 | 75 | 48 | 50.8982 | 1.30E+08 |
| 2 | 6 | 0 | 25 | 75 | 48 | 50.8982 | 1.20E+08 |
| 3 | 6 | 0 | 25 | 75 | 48 | 50.8982 | 1.46E+08 |
| 1 | 7 | 0 | 25 | 75 | 48 | 50.8982 | 4.40E+05 |
| 2 | 7 | 0 | 25 | 75 | 48 | 50.8982 | 4.50E+05 |
| 3 | 7 | 0 | 25 | 75 | 48 | 50.8982 | 4.75E+05 |
| 1 | 8 | 0 | 25 | 75 | 48 | 50.8982 | 8.77E+04 |
| 2 | 8 | 0 | 25 | 75 | 48 | 50.8982 | 8.08E+04 |
| 3 | 8 | 0 | 25 | 75 | 48 | 50.8982 | 8.80E+04 |
| 1 | 4 | 0.3 | 25 | 75 | 48 | 50.8982 | 7.10E+07 |
| 2 | 4 | 0.3 | 25 | 75 | 48 | 50.8982 | 7.46E+07 |
| 3 | 4 | 0.3 | 25 | 75 | 48 | 50.8982 | 7.15E+07 |
| 1 | 5 | 0.3 | 25 | 75 | 48 | 50.8982 | 8.60E+07 |
| 2 | 5 | 0.3 | 25 | 75 | 48 | 50.8982 | 8.20E+07 |
| 3 | 5 | 0.3 | 25 | 75 | 48 | 50.8982 | 8.81E+07 |
| 1 | 6 | 0.3 | 25 | 75 | 48 | 50.8982 | 2.50E+08 |
| 2 | 6 | 0.3 | 25 | 75 | 48 | 50.8982 | 2.40E+08 |
| 3 | 6 | 0.3 | 25 | 75 | 48 | 50.8982 | 2.60E+08 |
| 1 | 7 | 0.3 | 25 | 75 | 48 | 50.8982 | 9.60E+04 |
| 2 | 7 | 0.3 | 25 | 75 | 48 | 50.8982 | 9.45E+04 |
| 3 | 7 | 0.3 | 25 | 75 | 48 | 50.8982 | 9.00E+04 |
| 1 | 8 | 0.3 | 25 | 75 | 48 | 50.8982 | 4.62E+03 |
| 2 | 8 | 0.3 | 25 | 75 | 48 | 50.8982 | 4.30E+03 |
| 3 | 8 | 0.3 | 25 | 75 | 48 | 50.8982 | 4.30E+03 |
| 1 | 4 | 0.6 | 25 | 75 | 48 | 50.8982 | 8.10E+06 |
| 2 | 4 | 0.6 | 25 | 75 | 48 | 50.8982 | 8.40E+06 |
| 3 | 4 | 0.6 | 25 | 75 | 48 | 50.8982 | 8.20E+06 |
| 1 | 5 | 0.6 | 25 | 75 | 48 | 50.8982 | 1.20E+08 |
| 2 | 5 | 0.6 | 25 | 75 | 48 | 50.8982 | 1.00E+08 |
| 3 | 5 | 0.6 | 25 | 75 | 48 | 50.8982 | 1.06E+08 |
| 1 | 6 | 0.6 | 25 | 75 | 48 | 50.8982 | 1.20E+08 |
| 2 | 6 | 0.6 | 25 | 75 | 48 | 50.8982 | 1.31E+08 |
| 3 | 6 | 0.6 | 25 | 75 | 48 | 50.8982 | 1.40E+08 |
| 1 | 7 | 0.6 | 25 | 75 | 48 | 50.8982 | 6.75E+03 |
| 2 | 7 | 0.6 | 25 | 75 | 48 | 50.8982 | 7.76E+03 |
| 3 | 7 | 0.6 | 25 | 75 | 48 | 50.8982 | 6.30E+03 |
| 1 | 8 | 0.6 | 25 | 75 | 48 | 50.8982 | 2.02E+02 |
| 2 | 8 | 0.6 | 25 | 75 | 48 | 50.8982 | 1.26E+02 |
| 3 | 8 | 0.6 | 25 | 75 | 48 | 50.8982 | 2.35E+02 |
| 1 | 4 | 0.9 | 25 | 75 | 48 | 50.8982 | 1.60E+07 |
| 2 | 4 | 0.9 | 25 | 75 | 48 | 50.8982 | 1.12E+07 |
| 3 | 4 | 0.9 | 25 | 75 | 48 | 50.8982 | 1.50E+07 |
| 1 | 5 | 0.9 | 25 | 75 | 48 | 50.8982 | 9.20E+07 |
| 2 | 5 | 0.9 | 25 | 75 | 48 | 50.8982 | 9.55E+07 |
| 3 | 5 | 0.9 | 25 | 75 | 48 | 50.8982 | 9.00E+07 |
| 1 | 6 | 0.9 | 25 | 75 | 48 | 50.8982 | 6.33E+07 |
| 2 | 6 | 0.9 | 25 | 75 | 48 | 50.8982 | 6.20E+07 |
| 3 | 6 | 0.9 | 25 | 75 | 48 | 50.8982 | 6.00E+07 |
| 1 | 7 | 0.9 | 25 | 75 | 48 | 50.8982 | 1.68E+03 |
| 2 | 7 | 0.9 | 25 | 75 | 48 | 50.8982 | 1.30E+03 |
| 3 | 7 | 0.9 | 25 | 75 | 48 | 50.8982 | 1.16E+03 |
| 1 | 8 | 0.9 | 25 | 75 | 48 | 50.8982 | 2.91E+02 |
| 2 | 8 | 0.9 | 25 | 75 | 48 | 50.8982 | 2.74E+02 |
| 3 | 8 | 0.9 | 25 | 75 | 48 | 50.8982 | 2.40E+02 |
| 1 | 4 | 1.2 | 25 | 75 | 48 | 50.8982 | 9.22E+05 |
| 2 | 4 | 1.2 | 25 | 75 | 48 | 50.8982 | 8.32E+05 |
| 3 | 4 | 1.2 | 25 | 75 | 48 | 50.8982 | 8.37E+05 |
| 1 | 5 | 1.2 | 25 | 75 | 48 | 50.8982 | 3.60E+06 |
| 2 | 5 | 1.2 | 25 | 75 | 48 | 50.8982 | 3.74E+06 |
| 3 | 5 | 1.2 | 25 | 75 | 48 | 50.8982 | 4.20E+06 |
| 1 | 6 | 1.2 | 25 | 75 | 48 | 50.8982 | 3.40E+07 |
| 2 | 6 | 1.2 | 25 | 75 | 48 | 50.8982 | 2.97E+07 |
| 3 | 6 | 1.2 | 25 | 75 | 48 | 50.8982 | 3.48E+07 |
| 1 | 7 | 1.2 | 25 | 75 | 48 | 50.8982 | 2.30E+02 |
| 2 | 7 | 1.2 | 25 | 75 | 48 | 50.8982 | 2.06E+02 |
| 3 | 7 | 1.2 | 25 | 75 | 48 | 50.8982 | 2.17E+02 |
| 1 | 8 | 1.2 | 25 | 75 | 48 | 50.8982 | 4.17E+01 |
| 2 | 8 | 1.2 | 25 | 75 | 48 | 50.8982 | 4.50E+01 |
| 3 | 8 | 1.2 | 25 | 75 | 48 | 50.8982 | 4.20E+01 |
| 1 | 4 | 0 | 30 | 75 | 48 | 50.8982 | 2.11E+08 |
| 2 | 4 | 0 | 30 | 75 | 48 | 50.8982 | 2.00E+08 |
| 3 | 4 | 0 | 30 | 75 | 48 | 50.8982 | 1.93E+08 |
| 1 | 5 | 0 | 30 | 75 | 48 | 50.8982 | 5.00E+09 |
| 2 | 5 | 0 | 30 | 75 | 48 | 50.8982 | 4.84E+09 |
| 3 | 5 | 0 | 30 | 75 | 48 | 50.8982 | 5.15E+09 |
| 1 | 6 | 0 | 30 | 75 | 48 | 50.8982 | 4.88E+09 |
| 2 | 6 | 0 | 30 | 75 | 48 | 50.8982 | 5.30E+09 |
| 3 | 6 | 0 | 30 | 75 | 48 | 50.8982 | 4.80E+09 |
| 1 | 7 | 0 | 30 | 75 | 48 | 50.8982 | 2.81E+07 |
| 2 | 7 | 0 | 30 | 75 | 48 | 50.8982 | 3.00E+07 |
| 3 | 7 | 0 | 30 | 75 | 48 | 50.8982 | 3.20E+07 |
| 1 | 8 | 0 | 30 | 75 | 48 | 50.8982 | 1.40E+05 |
| 2 | 8 | 0 | 30 | 75 | 48 | 50.8982 | 1.65E+05 |
| 3 | 8 | 0 | 30 | 75 | 48 | 50.8982 | 1.74E+05 |
| 1 | 4 | 0.3 | 30 | 75 | 48 | 50.8982 | 2.19E+08 |
| 2 | 4 | 0.3 | 30 | 75 | 48 | 50.8982 | 1.86E+08 |
| 3 | 4 | 0.3 | 30 | 75 | 48 | 50.8982 | 2.08E+08 |
| 1 | 5 | 0.3 | 30 | 75 | 48 | 50.8982 | 4.42E+09 |
| 2 | 5 | 0.3 | 30 | 75 | 48 | 50.8982 | 3.70E+09 |
| 3 | 5 | 0.3 | 30 | 75 | 48 | 50.8982 | 4.20E+09 |
| 1 | 6 | 0.3 | 30 | 75 | 48 | 50.8982 | 2.10E+09 |
| 2 | 6 | 0.3 | 30 | 75 | 48 | 50.8982 | 1.88E+09 |
| 3 | 6 | 0.3 | 30 | 75 | 48 | 50.8982 | 2.33E+09 |
| 1 | 7 | 0.3 | 30 | 75 | 48 | 50.8982 | 4.19E+05 |
| 2 | 7 | 0.3 | 30 | 75 | 48 | 50.8982 | 4.40E+05 |
| 3 | 7 | 0.3 | 30 | 75 | 48 | 50.8982 | 3.84E+05 |
| 1 | 8 | 0.3 | 30 | 75 | 48 | 50.8982 | 1.04E+04 |
| 2 | 8 | 0.3 | 30 | 75 | 48 | 50.8982 | 1.28E+04 |
| 3 | 8 | 0.3 | 30 | 75 | 48 | 50.8982 | 1.19E+04 |
| 1 | 4 | 0.6 | 30 | 75 | 48 | 50.8982 | 3.16E+08 |
| 2 | 4 | 0.6 | 30 | 75 | 48 | 50.8982 | 3.70E+08 |
| 3 | 4 | 0.6 | 30 | 75 | 48 | 50.8982 | 3.31E+08 |
| 1 | 5 | 0.6 | 30 | 75 | 48 | 50.8982 | 6.49E+09 |
| 2 | 5 | 0.6 | 30 | 75 | 48 | 50.8982 | 6.68E+09 |
| 3 | 5 | 0.6 | 30 | 75 | 48 | 50.8982 | 6.77E+09 |
| 1 | 6 | 0.6 | 30 | 75 | 48 | 50.8982 | 7.20E+10 |
| 2 | 6 | 0.6 | 30 | 75 | 48 | 50.8982 | 6.55E+10 |
| 3 | 6 | 0.6 | 30 | 75 | 48 | 50.8982 | 6.20E+10 |
| 1 | 7 | 0.6 | 30 | 75 | 48 | 50.8982 | 7.83E+04 |
| 2 | 7 | 0.6 | 30 | 75 | 48 | 50.8982 | 7.58E+04 |
| 3 | 7 | 0.6 | 30 | 75 | 48 | 50.8982 | 7.80E+04 |
| 1 | 8 | 0.6 | 30 | 75 | 48 | 50.8982 | 1.77E+04 |
| 2 | 8 | 0.6 | 30 | 75 | 48 | 50.8982 | 1.50E+04 |
| 3 | 8 | 0.6 | 30 | 75 | 48 | 50.8982 | 1.32E+04 |
| 1 | 4 | 0.9 | 30 | 75 | 48 | 50.8982 | 3.79E+08 |
| 2 | 4 | 0.9 | 30 | 75 | 48 | 50.8982 | 3.47E+08 |
| 3 | 4 | 0.9 | 30 | 75 | 48 | 50.8982 | 3.70E+08 |
| 1 | 5 | 0.9 | 30 | 75 | 48 | 50.8982 | 8.52E+08 |
| 2 | 5 | 0.9 | 30 | 75 | 48 | 50.8982 | 7.96E+08 |
| 3 | 5 | 0.9 | 30 | 75 | 48 | 50.8982 | 8.45E+08 |
| 1 | 6 | 0.9 | 30 | 75 | 48 | 50.8982 | 7.20E+09 |
| 2 | 6 | 0.9 | 30 | 75 | 48 | 50.8982 | 6.30E+09 |
| 3 | 6 | 0.9 | 30 | 75 | 48 | 50.8982 | 6.44E+09 |
| 1 | 7 | 0.9 | 30 | 75 | 48 | 50.8982 | 6.75E+03 |
| 2 | 7 | 0.9 | 30 | 75 | 48 | 50.8982 | 6.50E+03 |
| 3 | 7 | 0.9 | 30 | 75 | 48 | 50.8982 | 6.68E+03 |
| 1 | 8 | 0.9 | 30 | 75 | 48 | 50.8982 | 2.00E+02 |
| 2 | 8 | 0.9 | 30 | 75 | 48 | 50.8982 | 2.09E+02 |
| 3 | 8 | 0.9 | 30 | 75 | 48 | 50.8982 | 1.91E+02 |
| 1 | 4 | 1.2 | 30 | 75 | 48 | 50.8982 | 1.47E+06 |
| 2 | 4 | 1.2 | 30 | 75 | 48 | 50.8982 | 1.50E+06 |
| 3 | 4 | 1.2 | 30 | 75 | 48 | 50.8982 | 1.15E+06 |
| 1 | 5 | 1.2 | 30 | 75 | 48 | 50.8982 | 1.57E+07 |
| 2 | 5 | 1.2 | 30 | 75 | 48 | 50.8982 | 1.12E+07 |
| 3 | 5 | 1.2 | 30 | 75 | 48 | 50.8982 | 1.20E+07 |
| 1 | 6 | 1.2 | 30 | 75 | 48 | 50.8982 | 1.12E+08 |
| 2 | 6 | 1.2 | 30 | 75 | 48 | 50.8982 | 1.05E+08 |
| 3 | 6 | 1.2 | 30 | 75 | 48 | 50.8982 | 1.23E+08 |
| 1 | 7 | 1.2 | 30 | 75 | 48 | 50.8982 | 3.80E+02 |
| 2 | 7 | 1.2 | 30 | 75 | 48 | 50.8982 | 3.70E+02 |
| 3 | 7 | 1.2 | 30 | 75 | 48 | 50.8982 | 4.14E+02 |
| 1 | 8 | 1.2 | 30 | 75 | 48 | 50.8982 | 4.20E+01 |
| 2 | 8 | 1.2 | 30 | 75 | 48 | 50.8982 | 3.60E+01 |
| 3 | 8 | 1.2 | 30 | 75 | 48 | 50.8982 | 3.30E+01 |
| 1 | 4 | 0 | 35 | 75 | 48 | 50.8982 | 7.00E+07 |
| 2 | 4 | 0 | 35 | 75 | 48 | 50.8982 | 6.80E+07 |
| 3 | 4 | 0 | 35 | 75 | 48 | 50.8982 | 6.67E+07 |
| 1 | 5 | 0 | 35 | 75 | 48 | 50.8982 | 8.65E+09 |
| 2 | 5 | 0 | 35 | 75 | 48 | 50.8982 | 8.30E+09 |
| 3 | 5 | 0 | 35 | 75 | 48 | 50.8982 | 8.40E+09 |
| 1 | 6 | 0 | 35 | 75 | 48 | 50.8982 | 1.23E+10 |
| 2 | 6 | 0 | 35 | 75 | 48 | 50.8982 | 1.17E+10 |
| 3 | 6 | 0 | 35 | 75 | 48 | 50.8982 | 1.27E+10 |
| 1 | 7 | 0 | 35 | 75 | 48 | 50.8982 | 7.36E+08 |
| 2 | 7 | 0 | 35 | 75 | 48 | 50.8982 | 7.20E+08 |
| 3 | 7 | 0 | 35 | 75 | 48 | 50.8982 | 7.48E+08 |
| 1 | 8 | 0 | 35 | 75 | 48 | 50.8982 | 4.88E+05 |
| 2 | 8 | 0 | 35 | 75 | 48 | 50.8982 | 4.61E+05 |
| 3 | 8 | 0 | 35 | 75 | 48 | 50.8982 | 5.05E+05 |
| 1 | 4 | 0.3 | 35 | 75 | 48 | 50.8982 | 2.33E+09 |
| 2 | 4 | 0.3 | 35 | 75 | 48 | 50.8982 | 2.30E+09 |
| 3 | 4 | 0.3 | 35 | 75 | 48 | 50.8982 | 2.45E+09 |
| 1 | 5 | 0.3 | 35 | 75 | 48 | 50.8982 | 7.50E+10 |
| 2 | 5 | 0.3 | 35 | 75 | 48 | 50.8982 | 7.38E+10 |
| 3 | 5 | 0.3 | 35 | 75 | 48 | 50.8982 | 7.44E+10 |
| 1 | 6 | 0.3 | 35 | 75 | 48 | 50.8982 | 1.36E+11 |
| 2 | 6 | 0.3 | 35 | 75 | 48 | 50.8982 | 1.18E+11 |
| 3 | 6 | 0.3 | 35 | 75 | 48 | 50.8982 | 1.20E+11 |
| 1 | 7 | 0.3 | 35 | 75 | 48 | 50.8982 | 9.24E+05 |
| 2 | 7 | 0.3 | 35 | 75 | 48 | 50.8982 | 8.27E+05 |
| 3 | 7 | 0.3 | 35 | 75 | 48 | 50.8982 | 8.43E+05 |
| 1 | 8 | 0.3 | 35 | 75 | 48 | 50.8982 | 3.25E+04 |
| 2 | 8 | 0.3 | 35 | 75 | 48 | 50.8982 | 3.61E+04 |
| 3 | 8 | 0.3 | 35 | 75 | 48 | 50.8982 | 3.40E+04 |
| 1 | 4 | 0.6 | 35 | 75 | 48 | 50.8982 | 4.24E+09 |
| 2 | 4 | 0.6 | 35 | 75 | 48 | 50.8982 | 4.13E+09 |
| 3 | 4 | 0.6 | 35 | 75 | 48 | 50.8982 | 4.63E+09 |
| 1 | 5 | 0.6 | 35 | 75 | 48 | 50.8982 | 5.14E+10 |
| 2 | 5 | 0.6 | 35 | 75 | 48 | 50.8982 | 5.85E+10 |
| 3 | 5 | 0.6 | 35 | 75 | 48 | 50.8982 | 5.50E+10 |
| 1 | 6 | 0.6 | 35 | 75 | 48 | 50.8982 | 6.20E+10 |
| 2 | 6 | 0.6 | 35 | 75 | 48 | 50.8982 | 6.40E+10 |
| 3 | 6 | 0.6 | 35 | 75 | 48 | 50.8982 | 6.25E+10 |
| 1 | 7 | 0.6 | 35 | 75 | 48 | 50.8982 | 4.11E+05 |
| 2 | 7 | 0.6 | 35 | 75 | 48 | 50.8982 | 4.16E+05 |
| 3 | 7 | 0.6 | 35 | 75 | 48 | 50.8982 | 4.33E+05 |
| 1 | 8 | 0.6 | 35 | 75 | 48 | 50.8982 | 7.28E+03 |
| 2 | 8 | 0.6 | 35 | 75 | 48 | 50.8982 | 7.74E+03 |
| 3 | 8 | 0.6 | 35 | 75 | 48 | 50.8982 | 7.30E+03 |
| 1 | 4 | 0.9 | 35 | 75 | 48 | 50.8982 | 7.12E+08 |
| 2 | 4 | 0.9 | 35 | 75 | 48 | 50.8982 | 7.00E+08 |
| 3 | 4 | 0.9 | 35 | 75 | 48 | 50.8982 | 7.00E+08 |
| 1 | 5 | 0.9 | 35 | 75 | 48 | 50.8982 | 8.73E+09 |
| 2 | 5 | 0.9 | 35 | 75 | 48 | 50.8982 | 9.05E+09 |
| 3 | 5 | 0.9 | 35 | 75 | 48 | 50.8982 | 8.72E+09 |
| 1 | 6 | 0.9 | 35 | 75 | 48 | 50.8982 | 3.85E+09 |
| 2 | 6 | 0.9 | 35 | 75 | 48 | 50.8982 | 4.00E+09 |
| 3 | 6 | 0.9 | 35 | 75 | 48 | 50.8982 | 3.90E+09 |
| 1 | 7 | 0.9 | 35 | 75 | 48 | 50.8982 | 6.10E+04 |
| 2 | 7 | 0.9 | 35 | 75 | 48 | 50.8982 | 6.74E+04 |
| 3 | 7 | 0.9 | 35 | 75 | 48 | 50.8982 | 7.14E+04 |
| 1 | 8 | 0.9 | 35 | 75 | 48 | 50.8982 | 4.38E+03 |
| 2 | 8 | 0.9 | 35 | 75 | 48 | 50.8982 | 4.50E+03 |
| 3 | 8 | 0.9 | 35 | 75 | 48 | 50.8982 | 4.30E+03 |
| 1 | 4 | 1.2 | 35 | 75 | 48 | 50.8982 | 1.15E+08 |
| 2 | 4 | 1.2 | 35 | 75 | 48 | 50.8982 | 1.09E+08 |
| 3 | 4 | 1.2 | 35 | 75 | 48 | 50.8982 | 1.01E+08 |
| 1 | 5 | 1.2 | 35 | 75 | 48 | 50.8982 | 7.40E+08 |
| 2 | 5 | 1.2 | 35 | 75 | 48 | 50.8982 | 7.00E+08 |
| 3 | 5 | 1.2 | 35 | 75 | 48 | 50.8982 | 6.80E+08 |
| 1 | 6 | 1.2 | 35 | 75 | 48 | 50.8982 | 4.80E+08 |
| 2 | 6 | 1.2 | 35 | 75 | 48 | 50.8982 | 4.69E+08 |
| 3 | 6 | 1.2 | 35 | 75 | 48 | 50.8982 | 3.80E+08 |
| 1 | 7 | 1.2 | 35 | 75 | 48 | 50.8982 | 6.37E+03 |
| 2 | 7 | 1.2 | 35 | 75 | 48 | 50.8982 | 7.50E+03 |
| 3 | 7 | 1.2 | 35 | 75 | 48 | 50.8982 | 6.50E+03 |
| 1 | 8 | 1.2 | 35 | 75 | 48 | 50.8982 | 1.20E+02 |
| 2 | 8 | 1.2 | 35 | 75 | 48 | 50.8982 | 1.26E+02 |
| 3 | 8 | 1.2 | 35 | 75 | 48 | 50.8982 | 1.04E+02 |
