# Supplementary material for: Interaction effects of physicochemical factors on the growth of Burkholderia pseudomallei in soil microcosms
Source: PLoS Negl Trop Dis. 2026 May 18;20(5):e0014339. doi: 10.1371/journal.pntd.0014339 (PMC13197065; doi:10.1371/journal.pntd.0014339)
Supplement: S3 Table — (DOCX) [file pntd.0014339.s009.docx]

**S3 Table.** Number of *B. pseudomallei* colonies on day 7 (N, CFU/mL) in three replicates under varying C/N ratio and salinity conditions at different soil temperatures and moisture contents, with pH and iron content kept constant (Salt: salinity of soil, Temp: temperature of soil, MC: moisture content of soil, C/N ratio: carbon to nitrogen ratio of soil, FE: iron content of soil).

| **Replicate** | **pH** | **Salt (%)** | **Temp (°C)** | **MC (%)** | **FE (mg/Kg)** | **C/N ratio** | **N (CFU/ml)** |
| --- | --- | --- | --- | --- | --- | --- | --- |
| 1 | 6.3 | 0 | 25 | 25 | 48 | 10 | 1.08E+08 |
| 2 | 6.3 | 0 | 25 | 25 | 48 | 10 | 1.03E+08 |
| 3 | 6.3 | 0 | 25 | 25 | 48 | 10 | 1.11E+08 |
| 1 | 6.3 | 0 | 25 | 25 | 48 | 25 | 1.24E+08 |
| 2 | 6.3 | 0 | 25 | 25 | 48 | 25 | 1.10E+08 |
| 3 | 6.3 | 0 | 25 | 25 | 48 | 25 | 1.04E+08 |
| 1 | 6.3 | 0 | 25 | 25 | 48 | 40 | 3.44E+06 |
| 2 | 6.3 | 0 | 25 | 25 | 48 | 40 | 4.20E+06 |
| 3 | 6.3 | 0 | 25 | 25 | 48 | 40 | 3.78E+06 |
| 1 | 6.3 | 0 | 25 | 25 | 48 | 55 | 3.20E+06 |
| 2 | 6.3 | 0 | 25 | 25 | 48 | 55 | 2.75E+06 |
| 3 | 6.3 | 0 | 25 | 25 | 48 | 55 | 3.00E+06 |
| 1 | 6.3 | 0 | 25 | 25 | 48 | 70 | 1.67E+06 |
| 2 | 6.3 | 0 | 25 | 25 | 48 | 70 | 1.81E+06 |
| 3 | 6.3 | 0 | 25 | 25 | 48 | 70 | 2.54E+06 |
| 1 | 6.3 | 0.3 | 25 | 25 | 48 | 10 | 2.22E+07 |
| 2 | 6.3 | 0.3 | 25 | 25 | 48 | 10 | 1.86E+07 |
| 3 | 6.3 | 0.3 | 25 | 25 | 48 | 10 | 1.94E+07 |
| 1 | 6.3 | 0.3 | 25 | 25 | 48 | 25 | 1.04E+07 |
| 2 | 6.3 | 0.3 | 25 | 25 | 48 | 25 | 1.28E+07 |
| 3 | 6.3 | 0.3 | 25 | 25 | 48 | 25 | 1.19E+07 |
| 1 | 6.3 | 0.3 | 25 | 25 | 48 | 40 | 4.82E+04 |
| 2 | 6.3 | 0.3 | 25 | 25 | 48 | 40 | 5.21E+04 |
| 3 | 6.3 | 0.3 | 25 | 25 | 48 | 40 | 5.00E+04 |
| 1 | 6.3 | 0.3 | 25 | 25 | 48 | 55 | 0.00E+00 |
| 2 | 6.3 | 0.3 | 25 | 25 | 48 | 55 | 0.00E+00 |
| 3 | 6.3 | 0.3 | 25 | 25 | 48 | 55 | 0.00E+00 |
| 1 | 6.3 | 0.3 | 25 | 25 | 48 | 70 | 0.00E+00 |
| 2 | 6.3 | 0.3 | 25 | 25 | 48 | 70 | 0.00E+00 |
| 3 | 6.3 | 0.3 | 25 | 25 | 48 | 70 | 0.00E+00 |
| 1 | 6.3 | 0.6 | 25 | 25 | 48 | 10 | 2.54E+04 |
| 2 | 6.3 | 0.6 | 25 | 25 | 48 | 10 | 2.61E+04 |
| 3 | 6.3 | 0.6 | 25 | 25 | 48 | 10 | 2.70E+04 |
| 1 | 6.3 | 0.6 | 25 | 25 | 48 | 25 | 3.33E+03 |
| 2 | 6.3 | 0.6 | 25 | 25 | 48 | 25 | 2.29E+03 |
| 3 | 6.3 | 0.6 | 25 | 25 | 48 | 25 | 2.50E+03 |
| 1 | 6.3 | 0.6 | 25 | 25 | 48 | 40 | 3.30E+02 |
| 2 | 6.3 | 0.6 | 25 | 25 | 48 | 40 | 4.09E+02 |
| 3 | 6.3 | 0.6 | 25 | 25 | 48 | 40 | 3.10E+02 |
| 1 | 6.3 | 0.6 | 25 | 25 | 48 | 55 | 0.00E+00 |
| 2 | 6.3 | 0.6 | 25 | 25 | 48 | 55 | 0.00E+00 |
| 3 | 6.3 | 0.6 | 25 | 25 | 48 | 55 | 0.00E+00 |
| 1 | 6.3 | 0.6 | 25 | 25 | 48 | 70 | 0.00E+00 |
| 2 | 6.3 | 0.6 | 25 | 25 | 48 | 70 | 0.00E+00 |
| 3 | 6.3 | 0.6 | 25 | 25 | 48 | 70 | 0.00E+00 |
| 1 | 6.3 | 0.9 | 25 | 25 | 48 | 10 | 1.60E+03 |
| 2 | 6.3 | 0.9 | 25 | 25 | 48 | 10 | 1.41E+03 |
| 3 | 6.3 | 0.9 | 25 | 25 | 48 | 10 | 1.45E+03 |
| 1 | 6.3 | 0.9 | 25 | 25 | 48 | 25 | 2.60E+03 |
| 2 | 6.3 | 0.9 | 25 | 25 | 48 | 25 | 1.79E+03 |
| 3 | 6.3 | 0.9 | 25 | 25 | 48 | 25 | 1.82E+03 |
| 1 | 6.3 | 0.9 | 25 | 25 | 48 | 40 | 0.00E+00 |
| 2 | 6.3 | 0.9 | 25 | 25 | 48 | 40 | 0.00E+00 |
| 3 | 6.3 | 0.9 | 25 | 25 | 48 | 40 | 0.00E+00 |
| 1 | 6.3 | 0.9 | 25 | 25 | 48 | 55 | 0.00E+00 |
| 2 | 6.3 | 0.9 | 25 | 25 | 48 | 55 | 0.00E+00 |
| 3 | 6.3 | 0.9 | 25 | 25 | 48 | 55 | 0.00E+00 |
| 1 | 6.3 | 0.9 | 25 | 25 | 48 | 70 | 0.00E+00 |
| 2 | 6.3 | 0.9 | 25 | 25 | 48 | 70 | 0.00E+00 |
| 3 | 6.3 | 0.9 | 25 | 25 | 48 | 70 | 0.00E+00 |
| 1 | 6.3 | 1.2 | 25 | 25 | 48 | 10 | 2.92E+03 |
| 2 | 6.3 | 1.2 | 25 | 25 | 48 | 10 | 2.86E+03 |
| 3 | 6.3 | 1.2 | 25 | 25 | 48 | 10 | 2.24E+03 |
| 1 | 6.3 | 1.2 | 25 | 25 | 48 | 25 | 3.03E+02 |
| 2 | 6.3 | 1.2 | 25 | 25 | 48 | 25 | 3.39E+02 |
| 3 | 6.3 | 1.2 | 25 | 25 | 48 | 25 | 3.17E+02 |
| 1 | 6.3 | 1.2 | 25 | 25 | 48 | 40 | 0.00E+00 |
| 2 | 6.3 | 1.2 | 25 | 25 | 48 | 40 | 0.00E+00 |
| 3 | 6.3 | 1.2 | 25 | 25 | 48 | 40 | 0.00E+00 |
| 1 | 6.3 | 1.2 | 25 | 25 | 48 | 55 | 0.00E+00 |
| 2 | 6.3 | 1.2 | 25 | 25 | 48 | 55 | 0.00E+00 |
| 3 | 6.3 | 1.2 | 25 | 25 | 48 | 55 | 0.00E+00 |
| 1 | 6.3 | 1.2 | 25 | 25 | 48 | 70 | 0.00E+00 |
| 2 | 6.3 | 1.2 | 25 | 25 | 48 | 70 | 0.00E+00 |
| 3 | 6.3 | 1.2 | 25 | 25 | 48 | 70 | 0.00E+00 |
| 1 | 6.3 | 0 | 30 | 25 | 48 | 10 | 1.63E+08 |
| 2 | 6.3 | 0 | 30 | 25 | 48 | 10 | 1.33E+08 |
| 3 | 6.3 | 0 | 30 | 25 | 48 | 10 | 1.88E+08 |
| 1 | 6.3 | 0 | 30 | 25 | 48 | 25 | 2.16E+08 |
| 2 | 6.3 | 0 | 30 | 25 | 48 | 25 | 2.19E+08 |
| 3 | 6.3 | 0 | 30 | 25 | 48 | 25 | 2.08E+08 |
| 1 | 6.3 | 0 | 30 | 25 | 48 | 40 | 3.24E+06 |
| 2 | 6.3 | 0 | 30 | 25 | 48 | 40 | 3.15E+06 |
| 3 | 6.3 | 0 | 30 | 25 | 48 | 40 | 3.44E+06 |
| 1 | 6.3 | 0 | 30 | 25 | 48 | 55 | 4.47E+06 |
| 2 | 6.3 | 0 | 30 | 25 | 48 | 55 | 5.19E+06 |
| 3 | 6.3 | 0 | 30 | 25 | 48 | 55 | 5.22E+06 |
| 1 | 6.3 | 0 | 30 | 25 | 48 | 70 | 2.87E+06 |
| 2 | 6.3 | 0 | 30 | 25 | 48 | 70 | 3.03E+06 |
| 3 | 6.3 | 0 | 30 | 25 | 48 | 70 | 2.66E+06 |
| 1 | 6.3 | 0.3 | 30 | 25 | 48 | 10 | 3.29E+06 |
| 2 | 6.3 | 0.3 | 30 | 25 | 48 | 10 | 2.65E+06 |
| 3 | 6.3 | 0.3 | 30 | 25 | 48 | 10 | 2.43E+06 |
| 1 | 6.3 | 0.3 | 30 | 25 | 48 | 25 | 2.92E+06 |
| 2 | 6.3 | 0.3 | 30 | 25 | 48 | 25 | 3.80E+06 |
| 3 | 6.3 | 0.3 | 30 | 25 | 48 | 25 | 2.75E+06 |
| 1 | 6.3 | 0.3 | 30 | 25 | 48 | 40 | 3.82E+04 |
| 2 | 6.3 | 0.3 | 30 | 25 | 48 | 40 | 3.92E+04 |
| 3 | 6.3 | 0.3 | 30 | 25 | 48 | 40 | 3.55E+04 |
| 1 | 6.3 | 0.3 | 30 | 25 | 48 | 55 | 4.30E+02 |
| 2 | 6.3 | 0.3 | 30 | 25 | 48 | 55 | 4.88E+02 |
| 3 | 6.3 | 0.3 | 30 | 25 | 48 | 55 | 4.40E+02 |
| 1 | 6.3 | 0.3 | 30 | 25 | 48 | 70 | 0.00E+00 |
| 2 | 6.3 | 0.3 | 30 | 25 | 48 | 70 | 0.00E+00 |
| 3 | 6.3 | 0.3 | 30 | 25 | 48 | 70 | 0.00E+00 |
| 1 | 6.3 | 0.6 | 30 | 25 | 48 | 10 | 3.18E+03 |
| 2 | 6.3 | 0.6 | 30 | 25 | 48 | 10 | 3.85E+03 |
| 3 | 6.3 | 0.6 | 30 | 25 | 48 | 10 | 4.11E+03 |
| 1 | 6.3 | 0.6 | 30 | 25 | 48 | 25 | 1.72E+04 |
| 2 | 6.3 | 0.6 | 30 | 25 | 48 | 25 | 1.63E+04 |
| 3 | 6.3 | 0.6 | 30 | 25 | 48 | 25 | 2.50E+04 |
| 1 | 6.3 | 0.6 | 30 | 25 | 48 | 40 | 3.82E+03 |
| 2 | 6.3 | 0.6 | 30 | 25 | 48 | 40 | 3.17E+03 |
| 3 | 6.3 | 0.6 | 30 | 25 | 48 | 40 | 4.30E+03 |
| 1 | 6.3 | 0.6 | 30 | 25 | 48 | 55 | 0.00E+00 |
| 2 | 6.3 | 0.6 | 30 | 25 | 48 | 55 | 0.00E+00 |
| 3 | 6.3 | 0.6 | 30 | 25 | 48 | 55 | 0.00E+00 |
| 1 | 6.3 | 0.6 | 30 | 25 | 48 | 70 | 0.00E+00 |
| 2 | 6.3 | 0.6 | 30 | 25 | 48 | 70 | 0.00E+00 |
| 3 | 6.3 | 0.6 | 30 | 25 | 48 | 70 | 0.00E+00 |
| 1 | 6.3 | 0.9 | 30 | 25 | 48 | 10 | 2.42E+03 |
| 2 | 6.3 | 0.9 | 30 | 25 | 48 | 10 | 4.25E+03 |
| 3 | 6.3 | 0.9 | 30 | 25 | 48 | 10 | 2.68E+03 |
| 1 | 6.3 | 0.9 | 30 | 25 | 48 | 25 | 6.20E+02 |
| 2 | 6.3 | 0.9 | 30 | 25 | 48 | 25 | 4.80E+02 |
| 3 | 6.3 | 0.9 | 30 | 25 | 48 | 25 | 5.50E+02 |
| 1 | 6.3 | 0.9 | 30 | 25 | 48 | 40 | 0.00E+00 |
| 2 | 6.3 | 0.9 | 30 | 25 | 48 | 40 | 0.00E+00 |
| 3 | 6.3 | 0.9 | 30 | 25 | 48 | 40 | 0.00E+00 |
| 1 | 6.3 | 0.9 | 30 | 25 | 48 | 55 | 0.00E+00 |
| 2 | 6.3 | 0.9 | 30 | 25 | 48 | 55 | 0.00E+00 |
| 3 | 6.3 | 0.9 | 30 | 25 | 48 | 55 | 0.00E+00 |
| 1 | 6.3 | 0.9 | 30 | 25 | 48 | 70 | 0.00E+00 |
| 2 | 6.3 | 0.9 | 30 | 25 | 48 | 70 | 0.00E+00 |
| 3 | 6.3 | 0.9 | 30 | 25 | 48 | 70 | 0.00E+00 |
| 1 | 6.3 | 1.2 | 30 | 25 | 48 | 10 | 5.33E+02 |
| 2 | 6.3 | 1.2 | 30 | 25 | 48 | 10 | 7.22E+02 |
| 3 | 6.3 | 1.2 | 30 | 25 | 48 | 10 | 8.40E+02 |
| 1 | 6.3 | 1.2 | 30 | 25 | 48 | 25 | 0.00E+00 |
| 2 | 6.3 | 1.2 | 30 | 25 | 48 | 25 | 0.00E+00 |
| 3 | 6.3 | 1.2 | 30 | 25 | 48 | 25 | 0.00E+00 |
| 1 | 6.3 | 1.2 | 30 | 25 | 48 | 40 | 0.00E+00 |
| 2 | 6.3 | 1.2 | 30 | 25 | 48 | 40 | 0.00E+00 |
| 3 | 6.3 | 1.2 | 30 | 25 | 48 | 40 | 0.00E+00 |
| 1 | 6.3 | 1.2 | 30 | 25 | 48 | 55 | 0.00E+00 |
| 2 | 6.3 | 1.2 | 30 | 25 | 48 | 55 | 0.00E+00 |
| 3 | 6.3 | 1.2 | 30 | 25 | 48 | 55 | 0.00E+00 |
| 1 | 6.3 | 1.2 | 30 | 25 | 48 | 70 | 0.00E+00 |
| 2 | 6.3 | 1.2 | 30 | 25 | 48 | 70 | 0.00E+00 |
| 3 | 6.3 | 1.2 | 30 | 25 | 48 | 70 | 0.00E+00 |
| 1 | 6.3 | 0 | 35 | 25 | 48 | 10 | 1.19E+08 |
| 2 | 6.3 | 0 | 35 | 25 | 48 | 10 | 1.19E+08 |
| 3 | 6.3 | 0 | 35 | 25 | 48 | 10 | 1.20E+08 |
| 1 | 6.3 | 0 | 35 | 25 | 48 | 25 | 5.80E+07 |
| 2 | 6.3 | 0 | 35 | 25 | 48 | 25 | 6.20E+07 |
| 3 | 6.3 | 0 | 35 | 25 | 48 | 25 | 6.00E+07 |
| 1 | 6.3 | 0 | 35 | 25 | 48 | 40 | 1.63E+06 |
| 2 | 6.3 | 0 | 35 | 25 | 48 | 40 | 1.87E+06 |
| 3 | 6.3 | 0 | 35 | 25 | 48 | 40 | 1.27E+06 |
| 1 | 6.3 | 0 | 35 | 25 | 48 | 55 | 2.11E+06 |
| 2 | 6.3 | 0 | 35 | 25 | 48 | 55 | 2.06E+06 |
| 3 | 6.3 | 0 | 35 | 25 | 48 | 55 | 2.20E+06 |
| 1 | 6.3 | 0 | 35 | 25 | 48 | 70 | 1.35E+05 |
| 2 | 6.3 | 0 | 35 | 25 | 48 | 70 | 1.55E+05 |
| 3 | 6.3 | 0 | 35 | 25 | 48 | 70 | 1.49E+05 |
| 1 | 6.3 | 0.3 | 35 | 25 | 48 | 10 | 2.25E+06 |
| 2 | 6.3 | 0.3 | 35 | 25 | 48 | 10 | 3.62E+06 |
| 3 | 6.3 | 0.3 | 35 | 25 | 48 | 10 | 2.77E+06 |
| 1 | 6.3 | 0.3 | 35 | 25 | 48 | 25 | 4.30E+06 |
| 2 | 6.3 | 0.3 | 35 | 25 | 48 | 25 | 3.50E+06 |
| 3 | 6.3 | 0.3 | 35 | 25 | 48 | 25 | 5.50E+06 |
| 1 | 6.3 | 0.3 | 35 | 25 | 48 | 40 | 4.56E+03 |
| 2 | 6.3 | 0.3 | 35 | 25 | 48 | 40 | 4.37E+03 |
| 3 | 6.3 | 0.3 | 35 | 25 | 48 | 40 | 4.81E+03 |
| 1 | 6.3 | 0.3 | 35 | 25 | 48 | 55 | 1.42E+03 |
| 2 | 6.3 | 0.3 | 35 | 25 | 48 | 55 | 1.36E+03 |
| 3 | 6.3 | 0.3 | 35 | 25 | 48 | 55 | 1.44E+03 |
| 1 | 6.3 | 0.3 | 35 | 25 | 48 | 70 | 0.00E+00 |
| 2 | 6.3 | 0.3 | 35 | 25 | 48 | 70 | 0.00E+00 |
| 3 | 6.3 | 0.3 | 35 | 25 | 48 | 70 | 0.00E+00 |
| 1 | 6.3 | 0.6 | 35 | 25 | 48 | 10 | 1.85E+03 |
| 2 | 6.3 | 0.6 | 35 | 25 | 48 | 10 | 1.48E+03 |
| 3 | 6.3 | 0.6 | 35 | 25 | 48 | 10 | 2.21E+03 |
| 1 | 6.3 | 0.6 | 35 | 25 | 48 | 25 | 2.20E+03 |
| 2 | 6.3 | 0.6 | 35 | 25 | 48 | 25 | 2.55E+03 |
| 3 | 6.3 | 0.6 | 35 | 25 | 48 | 25 | 2.40E+03 |
| 1 | 6.3 | 0.6 | 35 | 25 | 48 | 40 | 4.29E+02 |
| 2 | 6.3 | 0.6 | 35 | 25 | 48 | 40 | 5.11E+02 |
| 3 | 6.3 | 0.6 | 35 | 25 | 48 | 40 | 3.30E+02 |
| 1 | 6.3 | 0.6 | 35 | 25 | 48 | 55 | 0.00E+00 |
| 2 | 6.3 | 0.6 | 35 | 25 | 48 | 55 | 0.00E+00 |
| 3 | 6.3 | 0.6 | 35 | 25 | 48 | 55 | 0.00E+00 |
| 1 | 6.3 | 0.6 | 35 | 25 | 48 | 70 | 0.00E+00 |
| 2 | 6.3 | 0.6 | 35 | 25 | 48 | 70 | 0.00E+00 |
| 3 | 6.3 | 0.6 | 35 | 25 | 48 | 70 | 0.00E+00 |
| 1 | 6.3 | 0.9 | 35 | 25 | 48 | 10 | 3.20E+02 |
| 2 | 6.3 | 0.9 | 35 | 25 | 48 | 10 | 4.75E+02 |
| 3 | 6.3 | 0.9 | 35 | 25 | 48 | 10 | 3.00E+02 |
| 1 | 6.3 | 0.9 | 35 | 25 | 48 | 25 | 0.00E+00 |
| 2 | 6.3 | 0.9 | 35 | 25 | 48 | 25 | 0.00E+00 |
| 3 | 6.3 | 0.9 | 35 | 25 | 48 | 25 | 0.00E+00 |
| 1 | 6.3 | 0.9 | 35 | 25 | 48 | 40 | 0.00E+00 |
| 2 | 6.3 | 0.9 | 35 | 25 | 48 | 40 | 0.00E+00 |
| 3 | 6.3 | 0.9 | 35 | 25 | 48 | 40 | 0.00E+00 |
| 1 | 6.3 | 0.9 | 35 | 25 | 48 | 55 | 0.00E+00 |
| 2 | 6.3 | 0.9 | 35 | 25 | 48 | 55 | 0.00E+00 |
| 3 | 6.3 | 0.9 | 35 | 25 | 48 | 55 | 0.00E+00 |
| 1 | 6.3 | 0.9 | 35 | 25 | 48 | 70 | 0.00E+00 |
| 2 | 6.3 | 0.9 | 35 | 25 | 48 | 70 | 0.00E+00 |
| 3 | 6.3 | 0.9 | 35 | 25 | 48 | 70 | 0.00E+00 |
| 1 | 6.3 | 1.2 | 35 | 25 | 48 | 10 | 3.22E+02 |
| 2 | 6.3 | 1.2 | 35 | 25 | 48 | 10 | 3.88E+02 |
| 3 | 6.3 | 1.2 | 35 | 25 | 48 | 10 | 3.78E+02 |
| 1 | 6.3 | 1.2 | 35 | 25 | 48 | 25 | 0.00E+00 |
| 2 | 6.3 | 1.2 | 35 | 25 | 48 | 25 | 0.00E+00 |
| 3 | 6.3 | 1.2 | 35 | 25 | 48 | 25 | 0.00E+00 |
| 1 | 6.3 | 1.2 | 35 | 25 | 48 | 40 | 0.00E+00 |
| 2 | 6.3 | 1.2 | 35 | 25 | 48 | 40 | 0.00E+00 |
| 3 | 6.3 | 1.2 | 35 | 25 | 48 | 40 | 0.00E+00 |
| 1 | 6.3 | 1.2 | 35 | 25 | 48 | 55 | 0.00E+00 |
| 2 | 6.3 | 1.2 | 35 | 25 | 48 | 55 | 0.00E+00 |
| 3 | 6.3 | 1.2 | 35 | 25 | 48 | 55 | 0.00E+00 |
| 1 | 6.3 | 1.2 | 35 | 25 | 48 | 70 | 0.00E+00 |
| 2 | 6.3 | 1.2 | 35 | 25 | 48 | 70 | 0.00E+00 |
| 3 | 6.3 | 1.2 | 35 | 25 | 48 | 70 | 0.00E+00 |
| 1 | 6.3 | 0 | 25 | 50 | 48 | 10 | 3.66E+07 |
| 2 | 6.3 | 0 | 25 | 50 | 48 | 10 | 3.71E+07 |
| 3 | 6.3 | 0 | 25 | 50 | 48 | 10 | 3.92E+07 |
| 1 | 6.3 | 0 | 25 | 50 | 48 | 25 | 4.60E+07 |
| 2 | 6.3 | 0 | 25 | 50 | 48 | 25 | 4.40E+07 |
| 3 | 6.3 | 0 | 25 | 50 | 48 | 25 | 5.81E+07 |
| 1 | 6.3 | 0 | 25 | 50 | 48 | 40 | 3.30E+06 |
| 2 | 6.3 | 0 | 25 | 50 | 48 | 40 | 2.30E+06 |
| 3 | 6.3 | 0 | 25 | 50 | 48 | 40 | 3.12E+06 |
| 1 | 6.3 | 0 | 25 | 50 | 48 | 55 | 3.66E+04 |
| 2 | 6.3 | 0 | 25 | 50 | 48 | 55 | 3.90E+04 |
| 3 | 6.3 | 0 | 25 | 50 | 48 | 55 | 3.22E+04 |
| 1 | 6.3 | 0 | 25 | 50 | 48 | 70 | 2.48E+04 |
| 2 | 6.3 | 0 | 25 | 50 | 48 | 70 | 3.11E+04 |
| 3 | 6.3 | 0 | 25 | 50 | 48 | 70 | 3.25E+04 |
| 1 | 6.3 | 0.3 | 25 | 50 | 48 | 10 | 3.47E+07 |
| 2 | 6.3 | 0.3 | 25 | 50 | 48 | 10 | 3.88E+07 |
| 3 | 6.3 | 0.3 | 25 | 50 | 48 | 10 | 3.44E+07 |
| 1 | 6.3 | 0.3 | 25 | 50 | 48 | 25 | 3.65E+06 |
| 2 | 6.3 | 0.3 | 25 | 50 | 48 | 25 | 3.34E+06 |
| 3 | 6.3 | 0.3 | 25 | 50 | 48 | 25 | 3.74E+06 |
| 1 | 6.3 | 0.3 | 25 | 50 | 48 | 40 | 2.33E+04 |
| 2 | 6.3 | 0.3 | 25 | 50 | 48 | 40 | 3.45E+04 |
| 3 | 6.3 | 0.3 | 25 | 50 | 48 | 40 | 3.36E+04 |
| 1 | 6.3 | 0.3 | 25 | 50 | 48 | 55 | 2.44E+03 |
| 2 | 6.3 | 0.3 | 25 | 50 | 48 | 55 | 3.00E+03 |
| 3 | 6.3 | 0.3 | 25 | 50 | 48 | 55 | 2.54E+03 |
| 1 | 6.3 | 0.3 | 25 | 50 | 48 | 70 | 3.71E+03 |
| 2 | 6.3 | 0.3 | 25 | 50 | 48 | 70 | 4.22E+03 |
| 3 | 6.3 | 0.3 | 25 | 50 | 48 | 70 | 3.59E+03 |
| 1 | 6.3 | 0.6 | 25 | 50 | 48 | 10 | 3.95E+05 |
| 2 | 6.3 | 0.6 | 25 | 50 | 48 | 10 | 3.90E+05 |
| 3 | 6.3 | 0.6 | 25 | 50 | 48 | 10 | 4.33E+05 |
| 1 | 6.3 | 0.6 | 25 | 50 | 48 | 25 | 2.33E+06 |
| 2 | 6.3 | 0.6 | 25 | 50 | 48 | 25 | 4.25E+06 |
| 3 | 6.3 | 0.6 | 25 | 50 | 48 | 25 | 4.33E+06 |
| 1 | 6.3 | 0.6 | 25 | 50 | 48 | 40 | 3.77E+02 |
| 2 | 6.3 | 0.6 | 25 | 50 | 48 | 40 | 4.11E+02 |
| 3 | 6.3 | 0.6 | 25 | 50 | 48 | 40 | 3.55E+02 |
| 1 | 6.3 | 0.6 | 25 | 50 | 48 | 55 | 0.00E+00 |
| 2 | 6.3 | 0.6 | 25 | 50 | 48 | 55 | 0.00E+00 |
| 3 | 6.3 | 0.6 | 25 | 50 | 48 | 55 | 0.00E+00 |
| 1 | 6.3 | 0.6 | 25 | 50 | 48 | 70 | 0.00E+00 |
| 2 | 6.3 | 0.6 | 25 | 50 | 48 | 70 | 0.00E+00 |
| 3 | 6.3 | 0.6 | 25 | 50 | 48 | 70 | 0.00E+00 |
| 1 | 6.3 | 0.9 | 25 | 50 | 48 | 10 | 2.45E+04 |
| 2 | 6.3 | 0.9 | 25 | 50 | 48 | 10 | 2.93E+04 |
| 3 | 6.3 | 0.9 | 25 | 50 | 48 | 10 | 2.73E+04 |
| 1 | 6.3 | 0.9 | 25 | 50 | 48 | 25 | 3.59E+04 |
| 2 | 6.3 | 0.9 | 25 | 50 | 48 | 25 | 3.50E+04 |
| 3 | 6.3 | 0.9 | 25 | 50 | 48 | 25 | 4.09E+04 |
| 1 | 6.3 | 0.9 | 25 | 50 | 48 | 40 | 0.00E+00 |
| 2 | 6.3 | 0.9 | 25 | 50 | 48 | 40 | 0.00E+00 |
| 3 | 6.3 | 0.9 | 25 | 50 | 48 | 40 | 0.00E+00 |
| 1 | 6.3 | 0.9 | 25 | 50 | 48 | 55 | 0.00E+00 |
| 2 | 6.3 | 0.9 | 25 | 50 | 48 | 55 | 0.00E+00 |
| 3 | 6.3 | 0.9 | 25 | 50 | 48 | 55 | 0.00E+00 |
| 1 | 6.3 | 0.9 | 25 | 50 | 48 | 70 | 0.00E+00 |
| 2 | 6.3 | 0.9 | 25 | 50 | 48 | 70 | 0.00E+00 |
| 3 | 6.3 | 0.9 | 25 | 50 | 48 | 70 | 0.00E+00 |
| 1 | 6.3 | 1.2 | 25 | 50 | 48 | 10 | 3.42E+03 |
| 2 | 6.3 | 1.2 | 25 | 50 | 48 | 10 | 3.08E+03 |
| 3 | 6.3 | 1.2 | 25 | 50 | 48 | 10 | 2.42E+03 |
| 1 | 6.3 | 1.2 | 25 | 50 | 48 | 25 | 4.49E+03 |
| 2 | 6.3 | 1.2 | 25 | 50 | 48 | 25 | 5.57E+03 |
| 3 | 6.3 | 1.2 | 25 | 50 | 48 | 25 | 5.55E+03 |
| 1 | 6.3 | 1.2 | 25 | 50 | 48 | 40 | 0.00E+00 |
| 2 | 6.3 | 1.2 | 25 | 50 | 48 | 40 | 0.00E+00 |
| 3 | 6.3 | 1.2 | 25 | 50 | 48 | 40 | 0.00E+00 |
| 1 | 6.3 | 1.2 | 25 | 50 | 48 | 55 | 0.00E+00 |
| 2 | 6.3 | 1.2 | 25 | 50 | 48 | 55 | 0.00E+00 |
| 3 | 6.3 | 1.2 | 25 | 50 | 48 | 55 | 0.00E+00 |
| 1 | 6.3 | 1.2 | 25 | 50 | 48 | 70 | 0.00E+00 |
| 2 | 6.3 | 1.2 | 25 | 50 | 48 | 70 | 0.00E+00 |
| 3 | 6.3 | 1.2 | 25 | 50 | 48 | 70 | 0.00E+00 |
| 1 | 6.3 | 0 | 30 | 50 | 48 | 10 | 1.62E+08 |
| 2 | 6.3 | 0 | 30 | 50 | 48 | 10 | 1.22E+08 |
| 3 | 6.3 | 0 | 30 | 50 | 48 | 10 | 1.42E+08 |
| 1 | 6.3 | 0 | 30 | 50 | 48 | 25 | 2.36E+08 |
| 2 | 6.3 | 0 | 30 | 50 | 48 | 25 | 2.42E+08 |
| 3 | 6.3 | 0 | 30 | 50 | 48 | 25 | 2.35E+08 |
| 1 | 6.3 | 0 | 30 | 50 | 48 | 40 | 3.68E+06 |
| 2 | 6.3 | 0 | 30 | 50 | 48 | 40 | 3.52E+06 |
| 3 | 6.3 | 0 | 30 | 50 | 48 | 40 | 3.56E+06 |
| 1 | 6.3 | 0 | 30 | 50 | 48 | 55 | 3.37E+05 |
| 2 | 6.3 | 0 | 30 | 50 | 48 | 55 | 2.77E+05 |
| 3 | 6.3 | 0 | 30 | 50 | 48 | 55 | 2.82E+05 |
| 1 | 6.3 | 0 | 30 | 50 | 48 | 70 | 3.62E+04 |
| 2 | 6.3 | 0 | 30 | 50 | 48 | 70 | 3.22E+04 |
| 3 | 6.3 | 0 | 30 | 50 | 48 | 70 | 3.60E+04 |
| 1 | 6.3 | 0.3 | 30 | 50 | 48 | 10 | 2.45E+07 |
| 2 | 6.3 | 0.3 | 30 | 50 | 48 | 10 | 3.22E+07 |
| 3 | 6.3 | 0.3 | 30 | 50 | 48 | 10 | 3.50E+07 |
| 1 | 6.3 | 0.3 | 30 | 50 | 48 | 25 | 1.16E+08 |
| 2 | 6.3 | 0.3 | 30 | 50 | 48 | 25 | 1.03E+08 |
| 3 | 6.3 | 0.3 | 30 | 50 | 48 | 25 | 1.12E+08 |
| 1 | 6.3 | 0.3 | 30 | 50 | 48 | 40 | 3.90E+05 |
| 2 | 6.3 | 0.3 | 30 | 50 | 48 | 40 | 4.30E+05 |
| 3 | 6.3 | 0.3 | 30 | 50 | 48 | 40 | 3.88E+05 |
| 1 | 6.3 | 0.3 | 30 | 50 | 48 | 55 | 3.17E+04 |
| 2 | 6.3 | 0.3 | 30 | 50 | 48 | 55 | 3.39E+04 |
| 3 | 6.3 | 0.3 | 30 | 50 | 48 | 55 | 3.52E+04 |
| 1 | 6.3 | 0.3 | 30 | 50 | 48 | 70 | 4.56E+02 |
| 2 | 6.3 | 0.3 | 30 | 50 | 48 | 70 | 5.22E+02 |
| 3 | 6.3 | 0.3 | 30 | 50 | 48 | 70 | 5.37E+02 |
| 1 | 6.3 | 0.6 | 30 | 50 | 48 | 10 | 3.81E+06 |
| 2 | 6.3 | 0.6 | 30 | 50 | 48 | 10 | 4.63E+06 |
| 3 | 6.3 | 0.6 | 30 | 50 | 48 | 10 | 4.77E+06 |
| 1 | 6.3 | 0.6 | 30 | 50 | 48 | 25 | 3.51E+07 |
| 2 | 6.3 | 0.6 | 30 | 50 | 48 | 25 | 4.50E+07 |
| 3 | 6.3 | 0.6 | 30 | 50 | 48 | 25 | 4.66E+07 |
| 1 | 6.3 | 0.6 | 30 | 50 | 48 | 40 | 2.94E+05 |
| 2 | 6.3 | 0.6 | 30 | 50 | 48 | 40 | 3.37E+05 |
| 3 | 6.3 | 0.6 | 30 | 50 | 48 | 40 | 3.28E+05 |
| 1 | 6.3 | 0.6 | 30 | 50 | 48 | 55 | 7.58E+03 |
| 2 | 6.3 | 0.6 | 30 | 50 | 48 | 55 | 7.64E+03 |
| 3 | 6.3 | 0.6 | 30 | 50 | 48 | 55 | 8.24E+03 |
| 1 | 6.3 | 0.6 | 30 | 50 | 48 | 70 | 5.31E+02 |
| 2 | 6.3 | 0.6 | 30 | 50 | 48 | 70 | 4.95E+02 |
| 3 | 6.3 | 0.6 | 30 | 50 | 48 | 70 | 4.11E+02 |
| 1 | 6.3 | 0.9 | 30 | 50 | 48 | 10 | 3.52E+06 |
| 2 | 6.3 | 0.9 | 30 | 50 | 48 | 10 | 4.33E+06 |
| 3 | 6.3 | 0.9 | 30 | 50 | 48 | 10 | 3.60E+06 |
| 1 | 6.3 | 0.9 | 30 | 50 | 48 | 25 | 3.22E+04 |
| 2 | 6.3 | 0.9 | 30 | 50 | 48 | 25 | 3.67E+04 |
| 3 | 6.3 | 0.9 | 30 | 50 | 48 | 25 | 3.52E+04 |
| 1 | 6.3 | 0.9 | 30 | 50 | 48 | 40 | 3.39E+03 |
| 2 | 6.3 | 0.9 | 30 | 50 | 48 | 40 | 4.10E+03 |
| 3 | 6.3 | 0.9 | 30 | 50 | 48 | 40 | 3.80E+03 |
| 1 | 6.3 | 0.9 | 30 | 50 | 48 | 55 | 3.52E+02 |
| 2 | 6.3 | 0.9 | 30 | 50 | 48 | 55 | 3.88E+02 |
| 3 | 6.3 | 0.9 | 30 | 50 | 48 | 55 | 4.72E+02 |
| 1 | 6.3 | 0.9 | 30 | 50 | 48 | 70 | 0.00E+00 |
| 2 | 6.3 | 0.9 | 30 | 50 | 48 | 70 | 0.00E+00 |
| 3 | 6.3 | 0.9 | 30 | 50 | 48 | 70 | 0.00E+00 |
| 1 | 6.3 | 1.2 | 30 | 50 | 48 | 10 | 3.92E+03 |
| 2 | 6.3 | 1.2 | 30 | 50 | 48 | 10 | 4.13E+03 |
| 3 | 6.3 | 1.2 | 30 | 50 | 48 | 10 | 5.72E+03 |
| 1 | 6.3 | 1.2 | 30 | 50 | 48 | 25 | 3.44E+03 |
| 2 | 6.3 | 1.2 | 30 | 50 | 48 | 25 | 4.27E+03 |
| 3 | 6.3 | 1.2 | 30 | 50 | 48 | 25 | 4.06E+03 |
| 1 | 6.3 | 1.2 | 30 | 50 | 48 | 40 | 0.00E+00 |
| 2 | 6.3 | 1.2 | 30 | 50 | 48 | 40 | 0.00E+00 |
| 3 | 6.3 | 1.2 | 30 | 50 | 48 | 40 | 0.00E+00 |
| 1 | 6.3 | 1.2 | 30 | 50 | 48 | 55 | 0.00E+00 |
| 2 | 6.3 | 1.2 | 30 | 50 | 48 | 55 | 0.00E+00 |
| 3 | 6.3 | 1.2 | 30 | 50 | 48 | 55 | 0.00E+00 |
| 1 | 6.3 | 1.2 | 30 | 50 | 48 | 70 | 0.00E+00 |
| 2 | 6.3 | 1.2 | 30 | 50 | 48 | 70 | 0.00E+00 |
| 3 | 6.3 | 1.2 | 30 | 50 | 48 | 70 | 0.00E+00 |
| 1 | 6.3 | 0 | 35 | 50 | 48 | 10 | 2.22E+08 |
| 2 | 6.3 | 0 | 35 | 50 | 48 | 10 | 2.09E+08 |
| 3 | 6.3 | 0 | 35 | 50 | 48 | 10 | 1.77E+08 |
| 1 | 6.3 | 0 | 35 | 50 | 48 | 25 | 2.50E+08 |
| 2 | 6.3 | 0 | 35 | 50 | 48 | 25 | 2.40E+08 |
| 3 | 6.3 | 0 | 35 | 50 | 48 | 25 | 2.47E+08 |
| 1 | 6.3 | 0 | 35 | 50 | 48 | 40 | 2.36E+05 |
| 2 | 6.3 | 0 | 35 | 50 | 48 | 40 | 2.77E+05 |
| 3 | 6.3 | 0 | 35 | 50 | 48 | 40 | 3.00E+05 |
| 1 | 6.3 | 0 | 35 | 50 | 48 | 55 | 3.14E+04 |
| 2 | 6.3 | 0 | 35 | 50 | 48 | 55 | 3.00E+04 |
| 3 | 6.3 | 0 | 35 | 50 | 48 | 55 | 3.64E+04 |
| 1 | 6.3 | 0 | 35 | 50 | 48 | 70 | 3.70E+04 |
| 2 | 6.3 | 0 | 35 | 50 | 48 | 70 | 3.61E+04 |
| 3 | 6.3 | 0 | 35 | 50 | 48 | 70 | 3.44E+04 |
| 1 | 6.3 | 0.3 | 35 | 50 | 48 | 10 | 2.43E+07 |
| 2 | 6.3 | 0.3 | 35 | 50 | 48 | 10 | 1.59E+07 |
| 3 | 6.3 | 0.3 | 35 | 50 | 48 | 10 | 2.20E+07 |
| 1 | 6.3 | 0.3 | 35 | 50 | 48 | 25 | 2.60E+08 |
| 2 | 6.3 | 0.3 | 35 | 50 | 48 | 25 | 2.47E+08 |
| 3 | 6.3 | 0.3 | 35 | 50 | 48 | 25 | 2.58E+08 |
| 1 | 6.3 | 0.3 | 35 | 50 | 48 | 40 | 2.30E+05 |
| 2 | 6.3 | 0.3 | 35 | 50 | 48 | 40 | 3.21E+05 |
| 3 | 6.3 | 0.3 | 35 | 50 | 48 | 40 | 3.26E+05 |
| 1 | 6.3 | 0.3 | 35 | 50 | 48 | 55 | 2.87E+04 |
| 2 | 6.3 | 0.3 | 35 | 50 | 48 | 55 | 3.31E+04 |
| 3 | 6.3 | 0.3 | 35 | 50 | 48 | 55 | 3.00E+04 |
| 1 | 6.3 | 0.3 | 35 | 50 | 48 | 70 | 3.73E+02 |
| 2 | 6.3 | 0.3 | 35 | 50 | 48 | 70 | 3.32E+02 |
| 3 | 6.3 | 0.3 | 35 | 50 | 48 | 70 | 3.00E+02 |
| 1 | 6.3 | 0.6 | 35 | 50 | 48 | 10 | 2.03E+06 |
| 2 | 6.3 | 0.6 | 35 | 50 | 48 | 10 | 2.25E+06 |
| 3 | 6.3 | 0.6 | 35 | 50 | 48 | 10 | 2.40E+06 |
| 1 | 6.3 | 0.6 | 35 | 50 | 48 | 25 | 2.39E+07 |
| 2 | 6.3 | 0.6 | 35 | 50 | 48 | 25 | 3.21E+07 |
| 3 | 6.3 | 0.6 | 35 | 50 | 48 | 25 | 3.01E+07 |
| 1 | 6.3 | 0.6 | 35 | 50 | 48 | 40 | 4.30E+04 |
| 2 | 6.3 | 0.6 | 35 | 50 | 48 | 40 | 3.00E+04 |
| 3 | 6.3 | 0.6 | 35 | 50 | 48 | 40 | 4.57E+04 |
| 1 | 6.3 | 0.6 | 35 | 50 | 48 | 55 | 5.82E+03 |
| 2 | 6.3 | 0.6 | 35 | 50 | 48 | 55 | 5.22E+03 |
| 3 | 6.3 | 0.6 | 35 | 50 | 48 | 55 | 5.53E+03 |
| 1 | 6.3 | 0.6 | 35 | 50 | 48 | 70 | 3.36E+02 |
| 2 | 6.3 | 0.6 | 35 | 50 | 48 | 70 | 3.61E+02 |
| 3 | 6.3 | 0.6 | 35 | 50 | 48 | 70 | 4.02E+02 |
| 1 | 6.3 | 0.9 | 35 | 50 | 48 | 10 | 2.06E+04 |
| 2 | 6.3 | 0.9 | 35 | 50 | 48 | 10 | 1.61E+04 |
| 3 | 6.3 | 0.9 | 35 | 50 | 48 | 10 | 2.14E+04 |
| 1 | 6.3 | 0.9 | 35 | 50 | 48 | 25 | 2.70E+05 |
| 2 | 6.3 | 0.9 | 35 | 50 | 48 | 25 | 3.50E+05 |
| 3 | 6.3 | 0.9 | 35 | 50 | 48 | 25 | 3.17E+05 |
| 1 | 6.3 | 0.9 | 35 | 50 | 48 | 40 | 4.66E+02 |
| 2 | 6.3 | 0.9 | 35 | 50 | 48 | 40 | 5.98E+02 |
| 3 | 6.3 | 0.9 | 35 | 50 | 48 | 40 | 5.52E+02 |
| 1 | 6.3 | 0.9 | 35 | 50 | 48 | 55 | 0.00E+00 |
| 2 | 6.3 | 0.9 | 35 | 50 | 48 | 55 | 0.00E+00 |
| 3 | 6.3 | 0.9 | 35 | 50 | 48 | 55 | 0.00E+00 |
| 1 | 6.3 | 0.9 | 35 | 50 | 48 | 70 | 0.00E+00 |
| 2 | 6.3 | 0.9 | 35 | 50 | 48 | 70 | 0.00E+00 |
| 3 | 6.3 | 0.9 | 35 | 50 | 48 | 70 | 0.00E+00 |
| 1 | 6.3 | 1.2 | 35 | 50 | 48 | 10 | 4.66E+03 |
| 2 | 6.3 | 1.2 | 35 | 50 | 48 | 10 | 4.50E+03 |
| 3 | 6.3 | 1.2 | 35 | 50 | 48 | 10 | 4.95E+03 |
| 1 | 6.3 | 1.2 | 35 | 50 | 48 | 25 | 3.61E+02 |
| 2 | 6.3 | 1.2 | 35 | 50 | 48 | 25 | 3.40E+02 |
| 3 | 6.3 | 1.2 | 35 | 50 | 48 | 25 | 3.48E+02 |
| 1 | 6.3 | 1.2 | 35 | 50 | 48 | 40 | 0.00E+00 |
| 2 | 6.3 | 1.2 | 35 | 50 | 48 | 40 | 0.00E+00 |
| 3 | 6.3 | 1.2 | 35 | 50 | 48 | 40 | 0.00E+00 |
| 1 | 6.3 | 1.2 | 35 | 50 | 48 | 55 | 0.00E+00 |
| 2 | 6.3 | 1.2 | 35 | 50 | 48 | 55 | 0.00E+00 |
| 3 | 6.3 | 1.2 | 35 | 50 | 48 | 55 | 0.00E+00 |
| 1 | 6.3 | 1.2 | 35 | 50 | 48 | 70 | 0.00E+00 |
| 2 | 6.3 | 1.2 | 35 | 50 | 48 | 70 | 0.00E+00 |
| 3 | 6.3 | 1.2 | 35 | 50 | 48 | 70 | 0.00E+00 |
| 1 | 6.3 | 0 | 25 | 75 | 48 | 10 | 6.61E+07 |
| 2 | 6.3 | 0 | 25 | 75 | 48 | 10 | 7.01E+07 |
| 3 | 6.3 | 0 | 25 | 75 | 48 | 10 | 6.29E+07 |
| 1 | 6.3 | 0 | 25 | 75 | 48 | 25 | 7.60E+07 |
| 2 | 6.3 | 0 | 25 | 75 | 48 | 25 | 7.44E+07 |
| 3 | 6.3 | 0 | 25 | 75 | 48 | 25 | 8.10E+07 |
| 1 | 6.3 | 0 | 25 | 75 | 48 | 40 | 5.30E+07 |
| 2 | 6.3 | 0 | 25 | 75 | 48 | 40 | 3.33E+07 |
| 3 | 6.3 | 0 | 25 | 75 | 48 | 40 | 5.12E+07 |
| 1 | 6.3 | 0 | 25 | 75 | 48 | 55 | 4.66E+05 |
| 2 | 6.3 | 0 | 25 | 75 | 48 | 55 | 5.90E+05 |
| 3 | 6.3 | 0 | 25 | 75 | 48 | 55 | 5.22E+05 |
| 1 | 6.3 | 0 | 25 | 75 | 48 | 70 | 3.48E+05 |
| 2 | 6.3 | 0 | 25 | 75 | 48 | 70 | 3.77E+05 |
| 3 | 6.3 | 0 | 25 | 75 | 48 | 70 | 3.25E+05 |
| 1 | 6.3 | 0.3 | 25 | 75 | 48 | 10 | 4.67E+07 |
| 2 | 6.3 | 0.3 | 25 | 75 | 48 | 10 | 3.88E+07 |
| 3 | 6.3 | 0.3 | 25 | 75 | 48 | 10 | 4.40E+07 |
| 1 | 6.3 | 0.3 | 25 | 75 | 48 | 25 | 5.66E+07 |
| 2 | 6.3 | 0.3 | 25 | 75 | 48 | 25 | 5.43E+07 |
| 3 | 6.3 | 0.3 | 25 | 75 | 48 | 25 | 4.70E+07 |
| 1 | 6.3 | 0.3 | 25 | 75 | 48 | 40 | 2.56E+05 |
| 2 | 6.3 | 0.3 | 25 | 75 | 48 | 40 | 3.54E+05 |
| 3 | 6.3 | 0.3 | 25 | 75 | 48 | 40 | 3.63E+05 |
| 1 | 6.3 | 0.3 | 25 | 75 | 48 | 55 | 4.44E+04 |
| 2 | 6.3 | 0.3 | 25 | 75 | 48 | 55 | 5.00E+04 |
| 3 | 6.3 | 0.3 | 25 | 75 | 48 | 55 | 4.50E+04 |
| 1 | 6.3 | 0.3 | 25 | 75 | 48 | 70 | 1.73E+04 |
| 2 | 6.3 | 0.3 | 25 | 75 | 48 | 70 | 2.24E+04 |
| 3 | 6.3 | 0.3 | 25 | 75 | 48 | 70 | 1.95E+04 |
| 1 | 6.3 | 0.6 | 25 | 75 | 48 | 10 | 5.90E+06 |
| 2 | 6.3 | 0.6 | 25 | 75 | 48 | 10 | 4.89E+06 |
| 3 | 6.3 | 0.6 | 25 | 75 | 48 | 10 | 5.33E+06 |
| 1 | 6.3 | 0.6 | 25 | 75 | 48 | 25 | 3.20E+07 |
| 2 | 6.3 | 0.6 | 25 | 75 | 48 | 25 | 2.55E+07 |
| 3 | 6.3 | 0.6 | 25 | 75 | 48 | 25 | 2.43E+07 |
| 1 | 6.3 | 0.6 | 25 | 75 | 48 | 40 | 2.07E+04 |
| 2 | 6.3 | 0.6 | 25 | 75 | 48 | 40 | 3.71E+04 |
| 3 | 6.3 | 0.6 | 25 | 75 | 48 | 40 | 2.75E+04 |
| 1 | 6.3 | 0.6 | 25 | 75 | 48 | 55 | 1.05E+03 |
| 2 | 6.3 | 0.6 | 25 | 75 | 48 | 55 | 8.00E+02 |
| 3 | 6.3 | 0.6 | 25 | 75 | 48 | 55 | 7.54E+02 |
| 1 | 6.3 | 0.6 | 25 | 75 | 48 | 70 | 3.75E+02 |
| 2 | 6.3 | 0.6 | 25 | 75 | 48 | 70 | 4.50E+02 |
| 3 | 6.3 | 0.6 | 25 | 75 | 48 | 70 | 3.43E+02 |
| 1 | 6.3 | 0.9 | 25 | 75 | 48 | 10 | 4.45E+05 |
| 2 | 6.3 | 0.9 | 25 | 75 | 48 | 10 | 3.89E+05 |
| 3 | 6.3 | 0.9 | 25 | 75 | 48 | 10 | 3.17E+05 |
| 1 | 6.3 | 0.9 | 25 | 75 | 48 | 25 | 3.02E+05 |
| 2 | 6.3 | 0.9 | 25 | 75 | 48 | 25 | 4.21E+05 |
| 3 | 6.3 | 0.9 | 25 | 75 | 48 | 25 | 4.50E+05 |
| 1 | 6.3 | 0.9 | 25 | 75 | 48 | 40 | 0.00E+00 |
| 2 | 6.3 | 0.9 | 25 | 75 | 48 | 40 | 0.00E+00 |
| 3 | 6.3 | 0.9 | 25 | 75 | 48 | 40 | 0.00E+00 |
| 1 | 6.3 | 0.9 | 25 | 75 | 48 | 55 | 0.00E+00 |
| 2 | 6.3 | 0.9 | 25 | 75 | 48 | 55 | 0.00E+00 |
| 3 | 6.3 | 0.9 | 25 | 75 | 48 | 55 | 0.00E+00 |
| 1 | 6.3 | 0.9 | 25 | 75 | 48 | 70 | 0.00E+00 |
| 2 | 6.3 | 0.9 | 25 | 75 | 48 | 70 | 0.00E+00 |
| 3 | 6.3 | 0.9 | 25 | 75 | 48 | 70 | 0.00E+00 |
| 1 | 6.3 | 1.2 | 25 | 75 | 48 | 10 | 5.11E+04 |
| 2 | 6.3 | 1.2 | 25 | 75 | 48 | 10 | 5.44E+04 |
| 3 | 6.3 | 1.2 | 25 | 75 | 48 | 10 | 6.50E+04 |
| 1 | 6.3 | 1.2 | 25 | 75 | 48 | 25 | 4.00E+04 |
| 2 | 6.3 | 1.2 | 25 | 75 | 48 | 25 | 5.70E+04 |
| 3 | 6.3 | 1.2 | 25 | 75 | 48 | 25 | 4.89E+04 |
| 1 | 6.3 | 1.2 | 25 | 75 | 48 | 40 | 0.00E+00 |
| 2 | 6.3 | 1.2 | 25 | 75 | 48 | 40 | 0.00E+00 |
| 3 | 6.3 | 1.2 | 25 | 75 | 48 | 40 | 0.00E+00 |
| 1 | 6.3 | 1.2 | 25 | 75 | 48 | 55 | 0.00E+00 |
| 2 | 6.3 | 1.2 | 25 | 75 | 48 | 55 | 0.00E+00 |
| 3 | 6.3 | 1.2 | 25 | 75 | 48 | 55 | 0.00E+00 |
| 1 | 6.3 | 1.2 | 25 | 75 | 48 | 70 | 0.00E+00 |
| 2 | 6.3 | 1.2 | 25 | 75 | 48 | 70 | 0.00E+00 |
| 3 | 6.3 | 1.2 | 25 | 75 | 48 | 70 | 0.00E+00 |
| 1 | 6.3 | 0 | 30 | 75 | 48 | 10 | 2.60E+09 |
| 2 | 6.3 | 0 | 30 | 75 | 48 | 10 | 2.20E+09 |
| 3 | 6.3 | 0 | 30 | 75 | 48 | 10 | 2.40E+09 |
| 1 | 6.3 | 0 | 30 | 75 | 48 | 25 | 1.36E+09 |
| 2 | 6.3 | 0 | 30 | 75 | 48 | 25 | 1.42E+09 |
| 3 | 6.3 | 0 | 30 | 75 | 48 | 25 | 1.35E+09 |
| 1 | 6.3 | 0 | 30 | 75 | 48 | 40 | 5.68E+07 |
| 2 | 6.3 | 0 | 30 | 75 | 48 | 40 | 5.20E+07 |
| 3 | 6.3 | 0 | 30 | 75 | 48 | 40 | 5.60E+07 |
| 1 | 6.3 | 0 | 30 | 75 | 48 | 55 | 2.73E+06 |
| 2 | 6.3 | 0 | 30 | 75 | 48 | 55 | 4.77E+06 |
| 3 | 6.3 | 0 | 30 | 75 | 48 | 55 | 3.82E+06 |
| 1 | 6.3 | 0 | 30 | 75 | 48 | 70 | 4.62E+05 |
| 2 | 6.3 | 0 | 30 | 75 | 48 | 70 | 4.22E+05 |
| 3 | 6.3 | 0 | 30 | 75 | 48 | 70 | 4.60E+05 |
| 1 | 6.3 | 0.3 | 30 | 75 | 48 | 10 | 2.48E+08 |
| 2 | 6.3 | 0.3 | 30 | 75 | 48 | 10 | 2.55E+08 |
| 3 | 6.3 | 0.3 | 30 | 75 | 48 | 10 | 2.20E+08 |
| 1 | 6.3 | 0.3 | 30 | 75 | 48 | 25 | 4.40E+09 |
| 2 | 6.3 | 0.3 | 30 | 75 | 48 | 25 | 4.54E+09 |
| 3 | 6.3 | 0.3 | 30 | 75 | 48 | 25 | 4.28E+09 |
| 1 | 6.3 | 0.3 | 30 | 75 | 48 | 40 | 2.91E+06 |
| 2 | 6.3 | 0.3 | 30 | 75 | 48 | 40 | 2.56E+06 |
| 3 | 6.3 | 0.3 | 30 | 75 | 48 | 40 | 2.67E+06 |
| 1 | 6.3 | 0.3 | 30 | 75 | 48 | 55 | 2.17E+05 |
| 2 | 6.3 | 0.3 | 30 | 75 | 48 | 55 | 1.93E+05 |
| 3 | 6.3 | 0.3 | 30 | 75 | 48 | 55 | 2.25E+05 |
| 1 | 6.3 | 0.3 | 30 | 75 | 48 | 70 | 6.50E+03 |
| 2 | 6.3 | 0.3 | 30 | 75 | 48 | 70 | 6.22E+03 |
| 3 | 6.3 | 0.3 | 30 | 75 | 48 | 70 | 6.37E+03 |
| 1 | 6.3 | 0.6 | 30 | 75 | 48 | 10 | 8.11E+07 |
| 2 | 6.3 | 0.6 | 30 | 75 | 48 | 10 | 8.63E+07 |
| 3 | 6.3 | 0.6 | 30 | 75 | 48 | 10 | 7.79E+07 |
| 1 | 6.3 | 0.6 | 30 | 75 | 48 | 25 | 2.51E+08 |
| 2 | 6.3 | 0.6 | 30 | 75 | 48 | 25 | 2.50E+08 |
| 3 | 6.3 | 0.6 | 30 | 75 | 48 | 25 | 2.62E+08 |
| 1 | 6.3 | 0.6 | 30 | 75 | 48 | 40 | 4.49E+06 |
| 2 | 6.3 | 0.6 | 30 | 75 | 48 | 40 | 5.37E+06 |
| 3 | 6.3 | 0.6 | 30 | 75 | 48 | 40 | 5.28E+06 |
| 1 | 6.3 | 0.6 | 30 | 75 | 48 | 55 | 5.80E+04 |
| 2 | 6.3 | 0.6 | 30 | 75 | 48 | 55 | 4.67E+04 |
| 3 | 6.3 | 0.6 | 30 | 75 | 48 | 55 | 4.28E+04 |
| 1 | 6.3 | 0.6 | 30 | 75 | 48 | 70 | 1.13E+03 |
| 2 | 6.3 | 0.6 | 30 | 75 | 48 | 70 | 1.59E+03 |
| 3 | 6.3 | 0.6 | 30 | 75 | 48 | 70 | 1.10E+03 |
| 1 | 6.3 | 0.9 | 30 | 75 | 48 | 10 | 2.05E+05 |
| 2 | 6.3 | 0.9 | 30 | 75 | 48 | 10 | 1.43E+05 |
| 3 | 6.3 | 0.9 | 30 | 75 | 48 | 10 | 1.66E+05 |
| 1 | 6.3 | 0.9 | 30 | 75 | 48 | 25 | 3.20E+05 |
| 2 | 6.3 | 0.9 | 30 | 75 | 48 | 25 | 2.67E+05 |
| 3 | 6.3 | 0.9 | 30 | 75 | 48 | 25 | 2.75E+05 |
| 1 | 6.3 | 0.9 | 30 | 75 | 48 | 40 | 1.22E+04 |
| 2 | 6.3 | 0.9 | 30 | 75 | 48 | 40 | 1.62E+04 |
| 3 | 6.3 | 0.9 | 30 | 75 | 48 | 40 | 1.54E+04 |
| 1 | 6.3 | 0.9 | 30 | 75 | 48 | 55 | 2.25E+03 |
| 2 | 6.3 | 0.9 | 30 | 75 | 48 | 55 | 2.98E+03 |
| 3 | 6.3 | 0.9 | 30 | 75 | 48 | 55 | 2.77E+03 |
| 1 | 6.3 | 0.9 | 30 | 75 | 48 | 70 | 0.00E+00 |
| 2 | 6.3 | 0.9 | 30 | 75 | 48 | 70 | 0.00E+00 |
| 3 | 6.3 | 0.9 | 30 | 75 | 48 | 70 | 0.00E+00 |
| 1 | 6.3 | 1.2 | 30 | 75 | 48 | 10 | 2.99E+04 |
| 2 | 6.3 | 1.2 | 30 | 75 | 48 | 10 | 3.14E+04 |
| 3 | 6.3 | 1.2 | 30 | 75 | 48 | 10 | 2.27E+04 |
| 1 | 6.3 | 1.2 | 30 | 75 | 48 | 25 | 4.43E+04 |
| 2 | 6.3 | 1.2 | 30 | 75 | 48 | 25 | 4.72E+04 |
| 3 | 6.3 | 1.2 | 30 | 75 | 48 | 25 | 5.06E+04 |
| 1 | 6.3 | 1.2 | 30 | 75 | 48 | 40 | 0.00E+00 |
| 2 | 6.3 | 1.2 | 30 | 75 | 48 | 40 | 0.00E+00 |
| 3 | 6.3 | 1.2 | 30 | 75 | 48 | 40 | 0.00E+00 |
| 1 | 6.3 | 1.2 | 30 | 75 | 48 | 55 | 0.00E+00 |
| 2 | 6.3 | 1.2 | 30 | 75 | 48 | 55 | 0.00E+00 |
| 3 | 6.3 | 1.2 | 30 | 75 | 48 | 55 | 0.00E+00 |
| 1 | 6.3 | 1.2 | 30 | 75 | 48 | 70 | 0.00E+00 |
| 2 | 6.3 | 1.2 | 30 | 75 | 48 | 70 | 0.00E+00 |
| 3 | 6.3 | 1.2 | 30 | 75 | 48 | 70 | 0.00E+00 |
| 1 | 6.3 | 0 | 35 | 75 | 48 | 10 | 1.22E+09 |
| 2 | 6.3 | 0 | 35 | 75 | 48 | 10 | 1.09E+09 |
| 3 | 6.3 | 0 | 35 | 75 | 48 | 10 | 1.17E+09 |
| 1 | 6.3 | 0 | 35 | 75 | 48 | 25 | 4.50E+09 |
| 2 | 6.3 | 0 | 35 | 75 | 48 | 25 | 4.00E+09 |
| 3 | 6.3 | 0 | 35 | 75 | 48 | 25 | 4.70E+09 |
| 1 | 6.3 | 0 | 35 | 75 | 48 | 40 | 6.13E+06 |
| 2 | 6.3 | 0 | 35 | 75 | 48 | 40 | 6.70E+06 |
| 3 | 6.3 | 0 | 35 | 75 | 48 | 40 | 5.00E+06 |
| 1 | 6.3 | 0 | 35 | 75 | 48 | 55 | 4.14E+05 |
| 2 | 6.3 | 0 | 35 | 75 | 48 | 55 | 4.00E+05 |
| 3 | 6.3 | 0 | 35 | 75 | 48 | 55 | 4.60E+05 |
| 1 | 6.3 | 0 | 35 | 75 | 48 | 70 | 3.75E+05 |
| 2 | 6.3 | 0 | 35 | 75 | 48 | 70 | 2.67E+05 |
| 3 | 6.3 | 0 | 35 | 75 | 48 | 70 | 3.66E+05 |
| 1 | 6.3 | 0.3 | 35 | 75 | 48 | 10 | 1.43E+08 |
| 2 | 6.3 | 0.3 | 35 | 75 | 48 | 10 | 1.50E+08 |
| 3 | 6.3 | 0.3 | 35 | 75 | 48 | 10 | 1.55E+08 |
| 1 | 6.3 | 0.3 | 35 | 75 | 48 | 25 | 4.60E+08 |
| 2 | 6.3 | 0.3 | 35 | 75 | 48 | 25 | 4.70E+08 |
| 3 | 6.3 | 0.3 | 35 | 75 | 48 | 25 | 4.59E+08 |
| 1 | 6.3 | 0.3 | 35 | 75 | 48 | 40 | 3.20E+06 |
| 2 | 6.3 | 0.3 | 35 | 75 | 48 | 40 | 3.12E+06 |
| 3 | 6.3 | 0.3 | 35 | 75 | 48 | 40 | 3.74E+06 |
| 1 | 6.3 | 0.3 | 35 | 75 | 48 | 55 | 3.87E+05 |
| 2 | 6.3 | 0.3 | 35 | 75 | 48 | 55 | 3.13E+05 |
| 3 | 6.3 | 0.3 | 35 | 75 | 48 | 55 | 4.00E+05 |
| 1 | 6.3 | 0.3 | 35 | 75 | 48 | 70 | 3.37E+03 |
| 2 | 6.3 | 0.3 | 35 | 75 | 48 | 70 | 2.33E+03 |
| 3 | 6.3 | 0.3 | 35 | 75 | 48 | 70 | 2.00E+03 |
| 1 | 6.3 | 0.6 | 35 | 75 | 48 | 10 | 3.02E+07 |
| 2 | 6.3 | 0.6 | 35 | 75 | 48 | 10 | 2.52E+07 |
| 3 | 6.3 | 0.6 | 35 | 75 | 48 | 10 | 2.48E+07 |
| 1 | 6.3 | 0.6 | 35 | 75 | 48 | 25 | 9.30E+07 |
| 2 | 6.3 | 0.6 | 35 | 75 | 48 | 25 | 1.23E+08 |
| 3 | 6.3 | 0.6 | 35 | 75 | 48 | 25 | 1.03E+08 |
| 1 | 6.3 | 0.6 | 35 | 75 | 48 | 40 | 3.40E+05 |
| 2 | 6.3 | 0.6 | 35 | 75 | 48 | 40 | 3.00E+05 |
| 3 | 6.3 | 0.6 | 35 | 75 | 48 | 40 | 4.75E+05 |
| 1 | 6.3 | 0.6 | 35 | 75 | 48 | 55 | 5.28E+03 |
| 2 | 6.3 | 0.6 | 35 | 75 | 48 | 55 | 5.22E+03 |
| 3 | 6.3 | 0.6 | 35 | 75 | 48 | 55 | 5.53E+03 |
| 1 | 6.3 | 0.6 | 35 | 75 | 48 | 70 | 4.80E+02 |
| 2 | 6.3 | 0.6 | 35 | 75 | 48 | 70 | 4.40E+02 |
| 3 | 6.3 | 0.6 | 35 | 75 | 48 | 70 | 4.39E+02 |
| 1 | 6.3 | 0.9 | 35 | 75 | 48 | 10 | 1.06E+05 |
| 2 | 6.3 | 0.9 | 35 | 75 | 48 | 10 | 1.61E+05 |
| 3 | 6.3 | 0.9 | 35 | 75 | 48 | 10 | 1.47E+05 |
| 1 | 6.3 | 0.9 | 35 | 75 | 48 | 25 | 3.70E+06 |
| 2 | 6.3 | 0.9 | 35 | 75 | 48 | 25 | 3.58E+06 |
| 3 | 6.3 | 0.9 | 35 | 75 | 48 | 25 | 3.17E+06 |
| 1 | 6.3 | 0.9 | 35 | 75 | 48 | 40 | 5.66E+03 |
| 2 | 6.3 | 0.9 | 35 | 75 | 48 | 40 | 5.89E+03 |
| 3 | 6.3 | 0.9 | 35 | 75 | 48 | 40 | 4.25E+03 |
| 1 | 6.3 | 0.9 | 35 | 75 | 48 | 55 | 3.20E+02 |
| 2 | 6.3 | 0.9 | 35 | 75 | 48 | 55 | 4.17E+02 |
| 3 | 6.3 | 0.9 | 35 | 75 | 48 | 55 | 3.55E+02 |
| 1 | 6.3 | 0.9 | 35 | 75 | 48 | 70 | 0.00E+00 |
| 2 | 6.3 | 0.9 | 35 | 75 | 48 | 70 | 0.00E+00 |
| 3 | 6.3 | 0.9 | 35 | 75 | 48 | 70 | 0.00E+00 |
| 1 | 6.3 | 1.2 | 35 | 75 | 48 | 10 | 6.63E+03 |
| 2 | 6.3 | 1.2 | 35 | 75 | 48 | 10 | 7.50E+03 |
| 3 | 6.3 | 1.2 | 35 | 75 | 48 | 10 | 6.95E+02 |
| 1 | 6.3 | 1.2 | 35 | 75 | 48 | 25 | 3.16E+03 |
| 2 | 6.3 | 1.2 | 35 | 75 | 48 | 25 | 3.00E+03 |
| 3 | 6.3 | 1.2 | 35 | 75 | 48 | 25 | 3.60E+03 |
| 1 | 6.3 | 1.2 | 35 | 75 | 48 | 40 | 0.00E+00 |
| 2 | 6.3 | 1.2 | 35 | 75 | 48 | 40 | 0.00E+00 |
| 3 | 6.3 | 1.2 | 35 | 75 | 48 | 40 | 0.00E+00 |
| 1 | 6.3 | 1.2 | 35 | 75 | 48 | 55 | 0.00E+00 |
| 2 | 6.3 | 1.2 | 35 | 75 | 48 | 55 | 0.00E+00 |
| 3 | 6.3 | 1.2 | 35 | 75 | 48 | 55 | 0.00E+00 |
| 1 | 6.3 | 1.2 | 35 | 75 | 48 | 70 | 0.00E+00 |
| 2 | 6.3 | 1.2 | 35 | 75 | 48 | 70 | 0.00E+00 |
| 3 | 6.3 | 1.2 | 35 | 75 | 48 | 70 | 0.00E+00 |
