# Supplementary material for: Interaction effects of physicochemical factors on the growth of Burkholderia pseudomallei in soil microcosms
Source: PLoS Negl Trop Dis. 2026 May 18;20(5):e0014339. doi: 10.1371/journal.pntd.0014339 (PMC13197065; doi:10.1371/journal.pntd.0014339)
Supplement: S4 Table — (DOCX) [file pntd.0014339.s010.docx]

**S4 Table.** Number of *B. pseudomallei* colonies on day 7 (N, CFU/mL) in three replicates under varying iron content and salinity conditions at different soil temperatures and moisture contents, with pH and iron content kept constant (Salt: salinity of soil, Temp: temperature of soil, MC: moisture content of soil, C/N ratio: carbon to nitrogen ratio of soil, FE: iron content of soil).

| **Replicate** | **pH** | **Salt (%)** | **Temp (°C)** | **MC (%)** | **FE (mg/Kg)** | **C/N ratio** | **N (CFU/ml)** |
| --- | --- | --- | --- | --- | --- | --- | --- |
| 1 | 6.3 | 0 | 25 | 25 | 50 | 50.8982 | 8.50E+07 |
| 2 | 6.3 | 0 | 25 | 25 | 50 | 50.8982 | 7.66E+07 |
| 3 | 6.3 | 0 | 25 | 25 | 50 | 50.8982 | 8.86E+07 |
| 1 | 6.3 | 0 | 25 | 25 | 100 | 50.8982 | 5.57E+07 |
| 2 | 6.3 | 0 | 25 | 25 | 100 | 50.8982 | 5.71E+07 |
| 3 | 6.3 | 0 | 25 | 25 | 100 | 50.8982 | 5.39E+07 |
| 1 | 6.3 | 0 | 25 | 25 | 200 | 50.8982 | 3.77E+07 |
| 2 | 6.3 | 0 | 25 | 25 | 200 | 50.8982 | 3.25E+07 |
| 3 | 6.3 | 0 | 25 | 25 | 200 | 50.8982 | 3.41E+07 |
| 1 | 6.3 | 0 | 25 | 25 | 300 | 50.8982 | 2.71E+07 |
| 2 | 6.3 | 0 | 25 | 25 | 300 | 50.8982 | 3.17E+07 |
| 3 | 6.3 | 0 | 25 | 25 | 300 | 50.8982 | 2.92E+07 |
| 1 | 6.3 | 0 | 25 | 25 | 400 | 50.8982 | 6.00E+05 |
| 2 | 6.3 | 0 | 25 | 25 | 400 | 50.8982 | 5.61E+05 |
| 3 | 6.3 | 0 | 25 | 25 | 400 | 50.8982 | 5.85E+05 |
| 1 | 6.3 | 0 | 25 | 25 | 500 | 50.8982 | 0.00E+00 |
| 2 | 6.3 | 0 | 25 | 25 | 500 | 50.8982 | 0.00E+00 |
| 3 | 6.3 | 0 | 25 | 25 | 500 | 50.8982 | 0.00E+00 |
| 1 | 6.3 | 0.3 | 25 | 25 | 50 | 50.8982 | 3.62E+05 |
| 2 | 6.3 | 0.3 | 25 | 25 | 50 | 50.8982 | 2.76E+05 |
| 3 | 6.3 | 0.3 | 25 | 25 | 50 | 50.8982 | 3.48E+05 |
| 1 | 6.3 | 0.3 | 25 | 25 | 100 | 50.8982 | 5.83E+05 |
| 2 | 6.3 | 0.3 | 25 | 25 | 100 | 50.8982 | 5.41E+05 |
| 3 | 6.3 | 0.3 | 25 | 25 | 100 | 50.8982 | 4.28E+05 |
| 1 | 6.3 | 0.3 | 25 | 25 | 200 | 50.8982 | 5.35E+05 |
| 2 | 6.3 | 0.3 | 25 | 25 | 200 | 50.8982 | 6.93E+05 |
| 3 | 6.3 | 0.3 | 25 | 25 | 200 | 50.8982 | 5.63E+05 |
| 1 | 6.3 | 0.3 | 25 | 25 | 300 | 50.8982 | 7.20E+04 |
| 2 | 6.3 | 0.3 | 25 | 25 | 300 | 50.8982 | 8.00E+04 |
| 3 | 6.3 | 0.3 | 25 | 25 | 300 | 50.8982 | 7.81E+04 |
| 1 | 6.3 | 0.3 | 25 | 25 | 400 | 50.8982 | 0.00E+00 |
| 2 | 6.3 | 0.3 | 25 | 25 | 400 | 50.8982 | 0.00E+00 |
| 3 | 6.3 | 0.3 | 25 | 25 | 400 | 50.8982 | 0.00E+00 |
| 1 | 6.3 | 0.3 | 25 | 25 | 500 | 50.8982 | 0.00E+00 |
| 2 | 6.3 | 0.3 | 25 | 25 | 500 | 50.8982 | 0.00E+00 |
| 3 | 6.3 | 0.3 | 25 | 25 | 500 | 50.8982 | 0.00E+00 |
| 1 | 6.3 | 0.6 | 25 | 25 | 50 | 50.8982 | 5.48E+03 |
| 2 | 6.3 | 0.6 | 25 | 25 | 50 | 50.8982 | 4.41E+03 |
| 3 | 6.3 | 0.6 | 25 | 25 | 50 | 50.8982 | 5.15E+03 |
| 1 | 6.3 | 0.6 | 25 | 25 | 100 | 50.8982 | 1.21E+03 |
| 2 | 6.3 | 0.6 | 25 | 25 | 100 | 50.8982 | 1.47E+03 |
| 3 | 6.3 | 0.6 | 25 | 25 | 100 | 50.8982 | 1.36E+03 |
| 1 | 6.3 | 0.6 | 25 | 25 | 200 | 50.8982 | 3.36E+03 |
| 2 | 6.3 | 0.6 | 25 | 25 | 200 | 50.8982 | 3.91E+03 |
| 3 | 6.3 | 0.6 | 25 | 25 | 200 | 50.8982 | 3.40E+03 |
| 1 | 6.3 | 0.6 | 25 | 25 | 300 | 50.8982 | 0.00E+00 |
| 2 | 6.3 | 0.6 | 25 | 25 | 300 | 50.8982 | 0.00E+00 |
| 3 | 6.3 | 0.6 | 25 | 25 | 300 | 50.8982 | 0.00E+00 |
| 1 | 6.3 | 0.6 | 25 | 25 | 400 | 50.8982 | 0.00E+00 |
| 2 | 6.3 | 0.6 | 25 | 25 | 400 | 50.8982 | 0.00E+00 |
| 3 | 6.3 | 0.6 | 25 | 25 | 400 | 50.8982 | 0.00E+00 |
| 1 | 6.3 | 0.6 | 25 | 25 | 500 | 50.8982 | 0.00E+00 |
| 2 | 6.3 | 0.6 | 25 | 25 | 500 | 50.8982 | 0.00E+00 |
| 3 | 6.3 | 0.6 | 25 | 25 | 500 | 50.8982 | 0.00E+00 |
| 1 | 6.3 | 0.9 | 25 | 25 | 50 | 50.8982 | 2.47E+03 |
| 2 | 6.3 | 0.9 | 25 | 25 | 50 | 50.8982 | 2.26E+03 |
| 3 | 6.3 | 0.9 | 25 | 25 | 50 | 50.8982 | 2.31E+03 |
| 1 | 6.3 | 0.9 | 25 | 25 | 100 | 50.8982 | 2.27E+03 |
| 2 | 6.3 | 0.9 | 25 | 25 | 100 | 50.8982 | 2.17E+03 |
| 3 | 6.3 | 0.9 | 25 | 25 | 100 | 50.8982 | 2.33E+03 |
| 1 | 6.3 | 0.9 | 25 | 25 | 200 | 50.8982 | 0.00E+00 |
| 2 | 6.3 | 0.9 | 25 | 25 | 200 | 50.8982 | 0.00E+00 |
| 3 | 6.3 | 0.9 | 25 | 25 | 200 | 50.8982 | 0.00E+00 |
| 1 | 6.3 | 0.9 | 25 | 25 | 300 | 50.8982 | 0.00E+00 |
| 2 | 6.3 | 0.9 | 25 | 25 | 300 | 50.8982 | 0.00E+00 |
| 3 | 6.3 | 0.9 | 25 | 25 | 300 | 50.8982 | 0.00E+00 |
| 1 | 6.3 | 0.9 | 25 | 25 | 400 | 50.8982 | 0.00E+00 |
| 2 | 6.3 | 0.9 | 25 | 25 | 400 | 50.8982 | 0.00E+00 |
| 3 | 6.3 | 0.9 | 25 | 25 | 400 | 50.8982 | 0.00E+00 |
| 1 | 6.3 | 0.9 | 25 | 25 | 500 | 50.8982 | 0.00E+00 |
| 2 | 6.3 | 0.9 | 25 | 25 | 500 | 50.8982 | 0.00E+00 |
| 3 | 6.3 | 0.9 | 25 | 25 | 500 | 50.8982 | 0.00E+00 |
| 1 | 6.3 | 1.2 | 25 | 25 | 50 | 50.8982 | 3.29E+02 |
| 2 | 6.3 | 1.2 | 25 | 25 | 50 | 50.8982 | 3.05E+02 |
| 3 | 6.3 | 1.2 | 25 | 25 | 50 | 50.8982 | 3.27E+02 |
| 1 | 6.3 | 1.2 | 25 | 25 | 100 | 50.8982 | 0.00E+00 |
| 2 | 6.3 | 1.2 | 25 | 25 | 100 | 50.8982 | 0.00E+00 |
| 3 | 6.3 | 1.2 | 25 | 25 | 100 | 50.8982 | 0.00E+00 |
| 1 | 6.3 | 1.2 | 25 | 25 | 200 | 50.8982 | 0.00E+00 |
| 2 | 6.3 | 1.2 | 25 | 25 | 200 | 50.8982 | 0.00E+00 |
| 3 | 6.3 | 1.2 | 25 | 25 | 200 | 50.8982 | 0.00E+00 |
| 1 | 6.3 | 1.2 | 25 | 25 | 300 | 50.8982 | 0.00E+00 |
| 2 | 6.3 | 1.2 | 25 | 25 | 300 | 50.8982 | 0.00E+00 |
| 3 | 6.3 | 1.2 | 25 | 25 | 300 | 50.8982 | 0.00E+00 |
| 1 | 6.3 | 1.2 | 25 | 25 | 400 | 50.8982 | 0.00E+00 |
| 2 | 6.3 | 1.2 | 25 | 25 | 400 | 50.8982 | 0.00E+00 |
| 3 | 6.3 | 1.2 | 25 | 25 | 400 | 50.8982 | 0.00E+00 |
| 1 | 6.3 | 1.2 | 25 | 25 | 500 | 50.8982 | 0.00E+00 |
| 2 | 6.3 | 1.2 | 25 | 25 | 500 | 50.8982 | 0.00E+00 |
| 3 | 6.3 | 1.2 | 25 | 25 | 500 | 50.8982 | 0.00E+00 |
| 1 | 6.3 | 0 | 30 | 25 | 50 | 50.8982 | 2.84E+07 |
| 2 | 6.3 | 0 | 30 | 25 | 50 | 50.8982 | 3.22E+07 |
| 3 | 6.3 | 0 | 30 | 25 | 50 | 50.8982 | 2.91E+07 |
| 1 | 6.3 | 0 | 30 | 25 | 100 | 50.8982 | 1.88E+08 |
| 2 | 6.3 | 0 | 30 | 25 | 100 | 50.8982 | 2.63E+08 |
| 3 | 6.3 | 0 | 30 | 25 | 100 | 50.8982 | 1.97E+08 |
| 1 | 6.3 | 0 | 30 | 25 | 200 | 50.8982 | 1.67E+07 |
| 2 | 6.3 | 0 | 30 | 25 | 200 | 50.8982 | 2.11E+07 |
| 3 | 6.3 | 0 | 30 | 25 | 200 | 50.8982 | 1.55E+07 |
| 1 | 6.3 | 0 | 30 | 25 | 300 | 50.8982 | 3.67E+07 |
| 2 | 6.3 | 0 | 30 | 25 | 300 | 50.8982 | 3.38E+07 |
| 3 | 6.3 | 0 | 30 | 25 | 300 | 50.8982 | 3.40E+07 |
| 1 | 6.3 | 0 | 30 | 25 | 400 | 50.8982 | 3.00E+07 |
| 2 | 6.3 | 0 | 30 | 25 | 400 | 50.8982 | 4.70E+07 |
| 3 | 6.3 | 0 | 30 | 25 | 400 | 50.8982 | 3.54E+07 |
| 1 | 6.3 | 0 | 30 | 25 | 500 | 50.8982 | 0.00E+00 |
| 2 | 6.3 | 0 | 30 | 25 | 500 | 50.8982 | 0.00E+00 |
| 3 | 6.3 | 0 | 30 | 25 | 500 | 50.8982 | 0.00E+00 |
| 1 | 6.3 | 0.3 | 30 | 25 | 50 | 50.8982 | 2.72E+05 |
| 2 | 6.3 | 0.3 | 30 | 25 | 50 | 50.8982 | 3.04E+05 |
| 3 | 6.3 | 0.3 | 30 | 25 | 50 | 50.8982 | 2.44E+05 |
| 1 | 6.3 | 0.3 | 30 | 25 | 100 | 50.8982 | 1.91E+06 |
| 2 | 6.3 | 0.3 | 30 | 25 | 100 | 50.8982 | 3.70E+05 |
| 3 | 6.3 | 0.3 | 30 | 25 | 100 | 50.8982 | 2.12E+05 |
| 1 | 6.3 | 0.3 | 30 | 25 | 200 | 50.8982 | 8.50E+04 |
| 2 | 6.3 | 0.3 | 30 | 25 | 200 | 50.8982 | 7.30E+04 |
| 3 | 6.3 | 0.3 | 30 | 25 | 200 | 50.8982 | 7.70E+04 |
| 1 | 6.3 | 0.3 | 30 | 25 | 300 | 50.8982 | 5.90E+04 |
| 2 | 6.3 | 0.3 | 30 | 25 | 300 | 50.8982 | 6.63E+04 |
| 3 | 6.3 | 0.3 | 30 | 25 | 300 | 50.8982 | 5.30E+04 |
| 1 | 6.3 | 0.3 | 30 | 25 | 400 | 50.8982 | 0.00E+00 |
| 2 | 6.3 | 0.3 | 30 | 25 | 400 | 50.8982 | 0.00E+00 |
| 3 | 6.3 | 0.3 | 30 | 25 | 400 | 50.8982 | 0.00E+00 |
| 1 | 6.3 | 0.3 | 30 | 25 | 500 | 50.8982 | 0.00E+00 |
| 2 | 6.3 | 0.3 | 30 | 25 | 500 | 50.8982 | 0.00E+00 |
| 3 | 6.3 | 0.3 | 30 | 25 | 500 | 50.8982 | 0.00E+00 |
| 1 | 6.3 | 0.6 | 30 | 25 | 50 | 50.8982 | 3.30E+02 |
| 2 | 6.3 | 0.6 | 30 | 25 | 50 | 50.8982 | 2.90E+02 |
| 3 | 6.3 | 0.6 | 30 | 25 | 50 | 50.8982 | 2.86E+02 |
| 1 | 6.3 | 0.6 | 30 | 25 | 100 | 50.8982 | 1.62E+03 |
| 2 | 6.3 | 0.6 | 30 | 25 | 100 | 50.8982 | 1.29E+03 |
| 3 | 6.3 | 0.6 | 30 | 25 | 100 | 50.8982 | 1.14E+03 |
| 1 | 6.3 | 0.6 | 30 | 25 | 200 | 50.8982 | 0.00E+00 |
| 2 | 6.3 | 0.6 | 30 | 25 | 200 | 50.8982 | 0.00E+00 |
| 3 | 6.3 | 0.6 | 30 | 25 | 200 | 50.8982 | 0.00E+00 |
| 1 | 6.3 | 0.6 | 30 | 25 | 300 | 50.8982 | 0.00E+00 |
| 2 | 6.3 | 0.6 | 30 | 25 | 300 | 50.8982 | 0.00E+00 |
| 3 | 6.3 | 0.6 | 30 | 25 | 300 | 50.8982 | 0.00E+00 |
| 1 | 6.3 | 0.6 | 30 | 25 | 400 | 50.8982 | 0.00E+00 |
| 2 | 6.3 | 0.6 | 30 | 25 | 400 | 50.8982 | 0.00E+00 |
| 3 | 6.3 | 0.6 | 30 | 25 | 400 | 50.8982 | 0.00E+00 |
| 1 | 6.3 | 0.6 | 30 | 25 | 500 | 50.8982 | 0.00E+00 |
| 2 | 6.3 | 0.6 | 30 | 25 | 500 | 50.8982 | 0.00E+00 |
| 3 | 6.3 | 0.6 | 30 | 25 | 500 | 50.8982 | 0.00E+00 |
| 1 | 6.3 | 0.9 | 30 | 25 | 50 | 50.8982 | 2.36E+02 |
| 2 | 6.3 | 0.9 | 30 | 25 | 50 | 50.8982 | 3.17E+02 |
| 3 | 6.3 | 0.9 | 30 | 25 | 50 | 50.8982 | 3.29E+02 |
| 1 | 6.3 | 0.9 | 30 | 25 | 100 | 50.8982 | 0.00E+00 |
| 2 | 6.3 | 0.9 | 30 | 25 | 100 | 50.8982 | 0.00E+00 |
| 3 | 6.3 | 0.9 | 30 | 25 | 100 | 50.8982 | 0.00E+00 |
| 1 | 6.3 | 0.9 | 30 | 25 | 200 | 50.8982 | 0.00E+00 |
| 2 | 6.3 | 0.9 | 30 | 25 | 200 | 50.8982 | 0.00E+00 |
| 3 | 6.3 | 0.9 | 30 | 25 | 200 | 50.8982 | 0.00E+00 |
| 1 | 6.3 | 0.9 | 30 | 25 | 300 | 50.8982 | 0.00E+00 |
| 2 | 6.3 | 0.9 | 30 | 25 | 300 | 50.8982 | 0.00E+00 |
| 3 | 6.3 | 0.9 | 30 | 25 | 300 | 50.8982 | 0.00E+00 |
| 1 | 6.3 | 0.9 | 30 | 25 | 400 | 50.8982 | 0.00E+00 |
| 2 | 6.3 | 0.9 | 30 | 25 | 400 | 50.8982 | 0.00E+00 |
| 3 | 6.3 | 0.9 | 30 | 25 | 400 | 50.8982 | 0.00E+00 |
| 1 | 6.3 | 0.9 | 30 | 25 | 500 | 50.8982 | 0.00E+00 |
| 2 | 6.3 | 0.9 | 30 | 25 | 500 | 50.8982 | 0.00E+00 |
| 3 | 6.3 | 0.9 | 30 | 25 | 500 | 50.8982 | 0.00E+00 |
| 1 | 6.3 | 1.2 | 30 | 25 | 50 | 50.8982 | 2.11E+02 |
| 2 | 6.3 | 1.2 | 30 | 25 | 50 | 50.8982 | 3.75E+02 |
| 3 | 6.3 | 1.2 | 30 | 25 | 50 | 50.8982 | 2.94E+02 |
| 1 | 6.3 | 1.2 | 30 | 25 | 100 | 50.8982 | 0.00E+00 |
| 2 | 6.3 | 1.2 | 30 | 25 | 100 | 50.8982 | 0.00E+00 |
| 3 | 6.3 | 1.2 | 30 | 25 | 100 | 50.8982 | 0.00E+00 |
| 1 | 6.3 | 1.2 | 30 | 25 | 200 | 50.8982 | 0.00E+00 |
| 2 | 6.3 | 1.2 | 30 | 25 | 200 | 50.8982 | 0.00E+00 |
| 3 | 6.3 | 1.2 | 30 | 25 | 200 | 50.8982 | 0.00E+00 |
| 1 | 6.3 | 1.2 | 30 | 25 | 300 | 50.8982 | 0.00E+00 |
| 2 | 6.3 | 1.2 | 30 | 25 | 300 | 50.8982 | 0.00E+00 |
| 3 | 6.3 | 1.2 | 30 | 25 | 300 | 50.8982 | 0.00E+00 |
| 1 | 6.3 | 1.2 | 30 | 25 | 400 | 50.8982 | 0.00E+00 |
| 2 | 6.3 | 1.2 | 30 | 25 | 400 | 50.8982 | 0.00E+00 |
| 3 | 6.3 | 1.2 | 30 | 25 | 400 | 50.8982 | 0.00E+00 |
| 1 | 6.3 | 1.2 | 30 | 25 | 500 | 50.8982 | 0.00E+00 |
| 2 | 6.3 | 1.2 | 30 | 25 | 500 | 50.8982 | 0.00E+00 |
| 3 | 6.3 | 1.2 | 30 | 25 | 500 | 50.8982 | 0.00E+00 |
| 1 | 6.3 | 0 | 35 | 25 | 50 | 50.8982 | 1.56E+08 |
| 2 | 6.3 | 0 | 35 | 25 | 50 | 50.8982 | 1.39E+08 |
| 3 | 6.3 | 0 | 35 | 25 | 50 | 50.8982 | 1.30E+08 |
| 1 | 6.3 | 0 | 35 | 25 | 100 | 50.8982 | 7.20E+07 |
| 2 | 6.3 | 0 | 35 | 25 | 100 | 50.8982 | 6.90E+07 |
| 3 | 6.3 | 0 | 35 | 25 | 100 | 50.8982 | 7.44E+07 |
| 1 | 6.3 | 0 | 35 | 25 | 200 | 50.8982 | 5.28E+07 |
| 2 | 6.3 | 0 | 35 | 25 | 200 | 50.8982 | 5.12E+07 |
| 3 | 6.3 | 0 | 35 | 25 | 200 | 50.8982 | 5.61E+07 |
| 1 | 6.3 | 0 | 35 | 25 | 300 | 50.8982 | 5.60E+07 |
| 2 | 6.3 | 0 | 35 | 25 | 300 | 50.8982 | 6.00E+07 |
| 3 | 6.3 | 0 | 35 | 25 | 300 | 50.8982 | 4.64E+07 |
| 1 | 6.3 | 0 | 35 | 25 | 400 | 50.8982 | 2.01E+07 |
| 2 | 6.3 | 0 | 35 | 25 | 400 | 50.8982 | 1.85E+07 |
| 3 | 6.3 | 0 | 35 | 25 | 400 | 50.8982 | 2.01E+07 |
| 1 | 6.3 | 0 | 35 | 25 | 500 | 50.8982 | 1.37E+02 |
| 2 | 6.3 | 0 | 35 | 25 | 500 | 50.8982 | 2.00E+02 |
| 3 | 6.3 | 0 | 35 | 25 | 500 | 50.8982 | 1.80E+02 |
| 1 | 6.3 | 0.3 | 35 | 25 | 50 | 50.8982 | 5.22E+05 |
| 2 | 6.3 | 0.3 | 35 | 25 | 50 | 50.8982 | 6.70E+05 |
| 3 | 6.3 | 0.3 | 35 | 25 | 50 | 50.8982 | 5.83E+05 |
| 1 | 6.3 | 0.3 | 35 | 25 | 100 | 50.8982 | 1.38E+06 |
| 2 | 6.3 | 0.3 | 35 | 25 | 100 | 50.8982 | 1.33E+06 |
| 3 | 6.3 | 0.3 | 35 | 25 | 100 | 50.8982 | 2.50E+06 |
| 1 | 6.3 | 0.3 | 35 | 25 | 200 | 50.8982 | 8.90E+05 |
| 2 | 6.3 | 0.3 | 35 | 25 | 200 | 50.8982 | 9.30E+05 |
| 3 | 6.3 | 0.3 | 35 | 25 | 200 | 50.8982 | 8.10E+05 |
| 1 | 6.3 | 0.3 | 35 | 25 | 300 | 50.8982 | 9.30E+04 |
| 2 | 6.3 | 0.3 | 35 | 25 | 300 | 50.8982 | 1.01E+05 |
| 3 | 6.3 | 0.3 | 35 | 25 | 300 | 50.8982 | 9.54E+04 |
| 1 | 6.3 | 0.3 | 35 | 25 | 400 | 50.8982 | 0.00E+00 |
| 2 | 6.3 | 0.3 | 35 | 25 | 400 | 50.8982 | 0.00E+00 |
| 3 | 6.3 | 0.3 | 35 | 25 | 400 | 50.8982 | 0.00E+00 |
| 1 | 6.3 | 0.3 | 35 | 25 | 500 | 50.8982 | 0.00E+00 |
| 2 | 6.3 | 0.3 | 35 | 25 | 500 | 50.8982 | 0.00E+00 |
| 3 | 6.3 | 0.3 | 35 | 25 | 500 | 50.8982 | 0.00E+00 |
| 1 | 6.3 | 0.6 | 35 | 25 | 50 | 50.8982 | 3.90E+02 |
| 2 | 6.3 | 0.6 | 35 | 25 | 50 | 50.8982 | 4.50E+02 |
| 3 | 6.3 | 0.6 | 35 | 25 | 50 | 50.8982 | 4.22E+02 |
| 1 | 6.3 | 0.6 | 35 | 25 | 100 | 50.8982 | 2.00E+02 |
| 2 | 6.3 | 0.6 | 35 | 25 | 100 | 50.8982 | 1.71E+02 |
| 3 | 6.3 | 0.6 | 35 | 25 | 100 | 50.8982 | 2.25E+02 |
| 1 | 6.3 | 0.6 | 35 | 25 | 200 | 50.8982 | 0.00E+00 |
| 2 | 6.3 | 0.6 | 35 | 25 | 200 | 50.8982 | 0.00E+00 |
| 3 | 6.3 | 0.6 | 35 | 25 | 200 | 50.8982 | 0.00E+00 |
| 1 | 6.3 | 0.6 | 35 | 25 | 300 | 50.8982 | 0.00E+00 |
| 2 | 6.3 | 0.6 | 35 | 25 | 300 | 50.8982 | 0.00E+00 |
| 3 | 6.3 | 0.6 | 35 | 25 | 300 | 50.8982 | 0.00E+00 |
| 1 | 6.3 | 0.6 | 35 | 25 | 400 | 50.8982 | 0.00E+00 |
| 2 | 6.3 | 0.6 | 35 | 25 | 400 | 50.8982 | 0.00E+00 |
| 3 | 6.3 | 0.6 | 35 | 25 | 400 | 50.8982 | 0.00E+00 |
| 1 | 6.3 | 0.6 | 35 | 25 | 500 | 50.8982 | 0.00E+00 |
| 2 | 6.3 | 0.6 | 35 | 25 | 500 | 50.8982 | 0.00E+00 |
| 3 | 6.3 | 0.6 | 35 | 25 | 500 | 50.8982 | 0.00E+00 |
| 1 | 6.3 | 0.9 | 35 | 25 | 50 | 50.8982 | 1.60E+02 |
| 2 | 6.3 | 0.9 | 35 | 25 | 50 | 50.8982 | 1.00E+02 |
| 3 | 6.3 | 0.9 | 35 | 25 | 50 | 50.8982 | 1.48E+02 |
| 1 | 6.3 | 0.9 | 35 | 25 | 100 | 50.8982 | 0.00E+00 |
| 2 | 6.3 | 0.9 | 35 | 25 | 100 | 50.8982 | 0.00E+00 |
| 3 | 6.3 | 0.9 | 35 | 25 | 100 | 50.8982 | 0.00E+00 |
| 1 | 6.3 | 0.9 | 35 | 25 | 200 | 50.8982 | 0.00E+00 |
| 2 | 6.3 | 0.9 | 35 | 25 | 200 | 50.8982 | 0.00E+00 |
| 3 | 6.3 | 0.9 | 35 | 25 | 200 | 50.8982 | 0.00E+00 |
| 1 | 6.3 | 0.9 | 35 | 25 | 300 | 50.8982 | 0.00E+00 |
| 2 | 6.3 | 0.9 | 35 | 25 | 300 | 50.8982 | 0.00E+00 |
| 3 | 6.3 | 0.9 | 35 | 25 | 300 | 50.8982 | 0.00E+00 |
| 1 | 6.3 | 0.9 | 35 | 25 | 400 | 50.8982 | 0.00E+00 |
| 2 | 6.3 | 0.9 | 35 | 25 | 400 | 50.8982 | 0.00E+00 |
| 3 | 6.3 | 0.9 | 35 | 25 | 400 | 50.8982 | 0.00E+00 |
| 1 | 6.3 | 0.9 | 35 | 25 | 500 | 50.8982 | 0.00E+00 |
| 2 | 6.3 | 0.9 | 35 | 25 | 500 | 50.8982 | 0.00E+00 |
| 3 | 6.3 | 0.9 | 35 | 25 | 500 | 50.8982 | 0.00E+00 |
| 1 | 6.3 | 1.2 | 35 | 25 | 50 | 50.8982 | 3.29E+02 |
| 2 | 6.3 | 1.2 | 35 | 25 | 50 | 50.8982 | 3.17E+02 |
| 3 | 6.3 | 1.2 | 35 | 25 | 50 | 50.8982 | 2.95E+02 |
| 1 | 6.3 | 1.2 | 35 | 25 | 100 | 50.8982 | 0.00E+00 |
| 2 | 6.3 | 1.2 | 35 | 25 | 100 | 50.8982 | 0.00E+00 |
| 3 | 6.3 | 1.2 | 35 | 25 | 100 | 50.8982 | 0.00E+00 |
| 1 | 6.3 | 1.2 | 35 | 25 | 200 | 50.8982 | 0.00E+00 |
| 2 | 6.3 | 1.2 | 35 | 25 | 200 | 50.8982 | 0.00E+00 |
| 3 | 6.3 | 1.2 | 35 | 25 | 200 | 50.8982 | 0.00E+00 |
| 1 | 6.3 | 1.2 | 35 | 25 | 300 | 50.8982 | 0.00E+00 |
| 2 | 6.3 | 1.2 | 35 | 25 | 300 | 50.8982 | 0.00E+00 |
| 3 | 6.3 | 1.2 | 35 | 25 | 300 | 50.8982 | 0.00E+00 |
| 1 | 6.3 | 1.2 | 35 | 25 | 400 | 50.8982 | 0.00E+00 |
| 2 | 6.3 | 1.2 | 35 | 25 | 400 | 50.8982 | 0.00E+00 |
| 3 | 6.3 | 1.2 | 35 | 25 | 400 | 50.8982 | 0.00E+00 |
| 1 | 6.3 | 1.2 | 35 | 25 | 500 | 50.8982 | 0.00E+00 |
| 2 | 6.3 | 1.2 | 35 | 25 | 500 | 50.8982 | 0.00E+00 |
| 3 | 6.3 | 1.2 | 35 | 25 | 500 | 50.8982 | 0.00E+00 |
| 1 | 6.3 | 0 | 25 | 50 | 50 | 50.8982 | 2.85E+07 |
| 2 | 6.3 | 0 | 25 | 50 | 50 | 50.8982 | 2.72E+07 |
| 3 | 6.3 | 0 | 25 | 50 | 50 | 50.8982 | 3.73E+07 |
| 1 | 6.3 | 0 | 25 | 50 | 100 | 50.8982 | 4.80E+07 |
| 2 | 6.3 | 0 | 25 | 50 | 100 | 50.8982 | 4.17E+07 |
| 3 | 6.3 | 0 | 25 | 50 | 100 | 50.8982 | 4.25E+07 |
| 1 | 6.3 | 0 | 25 | 50 | 200 | 50.8982 | 4.70E+07 |
| 2 | 6.3 | 0 | 25 | 50 | 200 | 50.8982 | 4.43E+07 |
| 3 | 6.3 | 0 | 25 | 50 | 200 | 50.8982 | 5.00E+07 |
| 1 | 6.3 | 0 | 25 | 50 | 300 | 50.8982 | 2.86E+07 |
| 2 | 6.3 | 0 | 25 | 50 | 300 | 50.8982 | 2.95E+07 |
| 3 | 6.3 | 0 | 25 | 50 | 300 | 50.8982 | 3.91E+07 |
| 1 | 6.3 | 0 | 25 | 50 | 400 | 50.8982 | 4.33E+06 |
| 2 | 6.3 | 0 | 25 | 50 | 400 | 50.8982 | 4.50E+06 |
| 3 | 6.3 | 0 | 25 | 50 | 400 | 50.8982 | 4.71E+06 |
| 1 | 6.3 | 0 | 25 | 50 | 500 | 50.8982 | 4.50E+04 |
| 2 | 6.3 | 0 | 25 | 50 | 500 | 50.8982 | 5.07E+04 |
| 3 | 6.3 | 0 | 25 | 50 | 500 | 50.8982 | 5.30E+04 |
| 1 | 6.3 | 0.3 | 25 | 50 | 50 | 50.8982 | 4.88E+07 |
| 2 | 6.3 | 0.3 | 25 | 50 | 50 | 50.8982 | 4.30E+07 |
| 3 | 6.3 | 0.3 | 25 | 50 | 50 | 50.8982 | 4.78E+07 |
| 1 | 6.3 | 0.3 | 25 | 50 | 100 | 50.8982 | 6.27E+06 |
| 2 | 6.3 | 0.3 | 25 | 50 | 100 | 50.8982 | 5.95E+06 |
| 3 | 6.3 | 0.3 | 25 | 50 | 100 | 50.8982 | 5.70E+06 |
| 1 | 6.3 | 0.3 | 25 | 50 | 200 | 50.8982 | 2.45E+06 |
| 2 | 6.3 | 0.3 | 25 | 50 | 200 | 50.8982 | 2.52E+06 |
| 3 | 6.3 | 0.3 | 25 | 50 | 200 | 50.8982 | 2.61E+06 |
| 1 | 6.3 | 0.3 | 25 | 50 | 300 | 50.8982 | 2.40E+05 |
| 2 | 6.3 | 0.3 | 25 | 50 | 300 | 50.8982 | 2.44E+05 |
| 3 | 6.3 | 0.3 | 25 | 50 | 300 | 50.8982 | 2.27E+05 |
| 1 | 6.3 | 0.3 | 25 | 50 | 400 | 50.8982 | 2.68E+03 |
| 2 | 6.3 | 0.3 | 25 | 50 | 400 | 50.8982 | 2.93E+03 |
| 3 | 6.3 | 0.3 | 25 | 50 | 400 | 50.8982 | 4.30E+03 |
| 1 | 6.3 | 0.3 | 25 | 50 | 500 | 50.8982 | 0.00E+00 |
| 2 | 6.3 | 0.3 | 25 | 50 | 500 | 50.8982 | 0.00E+00 |
| 3 | 6.3 | 0.3 | 25 | 50 | 500 | 50.8982 | 0.00E+00 |
| 1 | 6.3 | 0.6 | 25 | 50 | 50 | 50.8982 | 2.00E+05 |
| 2 | 6.3 | 0.6 | 25 | 50 | 50 | 50.8982 | 2.23E+05 |
| 3 | 6.3 | 0.6 | 25 | 50 | 50 | 50.8982 | 2.15E+05 |
| 1 | 6.3 | 0.6 | 25 | 50 | 100 | 50.8982 | 5.50E+06 |
| 2 | 6.3 | 0.6 | 25 | 50 | 100 | 50.8982 | 6.43E+06 |
| 3 | 6.3 | 0.6 | 25 | 50 | 100 | 50.8982 | 5.67E+06 |
| 1 | 6.3 | 0.6 | 25 | 50 | 200 | 50.8982 | 3.30E+05 |
| 2 | 6.3 | 0.6 | 25 | 50 | 200 | 50.8982 | 2.66E+05 |
| 3 | 6.3 | 0.6 | 25 | 50 | 200 | 50.8982 | 2.45E+05 |
| 1 | 6.3 | 0.6 | 25 | 50 | 300 | 50.8982 | 0.00E+00 |
| 2 | 6.3 | 0.6 | 25 | 50 | 300 | 50.8982 | 0.00E+00 |
| 3 | 6.3 | 0.6 | 25 | 50 | 300 | 50.8982 | 0.00E+00 |
| 1 | 6.3 | 0.6 | 25 | 50 | 400 | 50.8982 | 0.00E+00 |
| 2 | 6.3 | 0.6 | 25 | 50 | 400 | 50.8982 | 0.00E+00 |
| 3 | 6.3 | 0.6 | 25 | 50 | 400 | 50.8982 | 0.00E+00 |
| 1 | 6.3 | 0.6 | 25 | 50 | 500 | 50.8982 | 0.00E+00 |
| 2 | 6.3 | 0.6 | 25 | 50 | 500 | 50.8982 | 0.00E+00 |
| 3 | 6.3 | 0.6 | 25 | 50 | 500 | 50.8982 | 0.00E+00 |
| 1 | 6.3 | 0.9 | 25 | 50 | 50 | 50.8982 | 3.40E+05 |
| 2 | 6.3 | 0.9 | 25 | 50 | 50 | 50.8982 | 3.71E+05 |
| 3 | 6.3 | 0.9 | 25 | 50 | 50 | 50.8982 | 3.86E+05 |
| 1 | 6.3 | 0.9 | 25 | 50 | 100 | 50.8982 | 2.71E+04 |
| 2 | 6.3 | 0.9 | 25 | 50 | 100 | 50.8982 | 2.07E+04 |
| 3 | 6.3 | 0.9 | 25 | 50 | 100 | 50.8982 | 1.99E+04 |
| 1 | 6.3 | 0.9 | 25 | 50 | 200 | 50.8982 | 6.00E+02 |
| 2 | 6.3 | 0.9 | 25 | 50 | 200 | 50.8982 | 7.20E+02 |
| 3 | 6.3 | 0.9 | 25 | 50 | 200 | 50.8982 | 6.20E+02 |
| 1 | 6.3 | 0.9 | 25 | 50 | 300 | 50.8982 | 0.00E+00 |
| 2 | 6.3 | 0.9 | 25 | 50 | 300 | 50.8982 | 0.00E+00 |
| 3 | 6.3 | 0.9 | 25 | 50 | 300 | 50.8982 | 0.00E+00 |
| 1 | 6.3 | 0.9 | 25 | 50 | 400 | 50.8982 | 0.00E+00 |
| 2 | 6.3 | 0.9 | 25 | 50 | 400 | 50.8982 | 0.00E+00 |
| 3 | 6.3 | 0.9 | 25 | 50 | 400 | 50.8982 | 0.00E+00 |
| 1 | 6.3 | 0.9 | 25 | 50 | 500 | 50.8982 | 0.00E+00 |
| 2 | 6.3 | 0.9 | 25 | 50 | 500 | 50.8982 | 0.00E+00 |
| 3 | 6.3 | 0.9 | 25 | 50 | 500 | 50.8982 | 0.00E+00 |
| 1 | 6.3 | 1.2 | 25 | 50 | 50 | 50.8982 | 1.80E+03 |
| 2 | 6.3 | 1.2 | 25 | 50 | 50 | 50.8982 | 2.06E+03 |
| 3 | 6.3 | 1.2 | 25 | 50 | 50 | 50.8982 | 1.47E+03 |
| 1 | 6.3 | 1.2 | 25 | 50 | 100 | 50.8982 | 0.00E+00 |
| 2 | 6.3 | 1.2 | 25 | 50 | 100 | 50.8982 | 0.00E+00 |
| 3 | 6.3 | 1.2 | 25 | 50 | 100 | 50.8982 | 0.00E+00 |
| 1 | 6.3 | 1.2 | 25 | 50 | 200 | 50.8982 | 0.00E+00 |
| 2 | 6.3 | 1.2 | 25 | 50 | 200 | 50.8982 | 0.00E+00 |
| 3 | 6.3 | 1.2 | 25 | 50 | 200 | 50.8982 | 0.00E+00 |
| 1 | 6.3 | 1.2 | 25 | 50 | 300 | 50.8982 | 0.00E+00 |
| 2 | 6.3 | 1.2 | 25 | 50 | 300 | 50.8982 | 0.00E+00 |
| 3 | 6.3 | 1.2 | 25 | 50 | 300 | 50.8982 | 0.00E+00 |
| 1 | 6.3 | 1.2 | 25 | 50 | 400 | 50.8982 | 0.00E+00 |
| 2 | 6.3 | 1.2 | 25 | 50 | 400 | 50.8982 | 0.00E+00 |
| 3 | 6.3 | 1.2 | 25 | 50 | 400 | 50.8982 | 0.00E+00 |
| 1 | 6.3 | 1.2 | 25 | 50 | 500 | 50.8982 | 0.00E+00 |
| 2 | 6.3 | 1.2 | 25 | 50 | 500 | 50.8982 | 0.00E+00 |
| 3 | 6.3 | 1.2 | 25 | 50 | 500 | 50.8982 | 0.00E+00 |
| 1 | 6.3 | 0 | 30 | 50 | 50 | 50.8982 | 1.68E+08 |
| 2 | 6.3 | 0 | 30 | 50 | 50 | 50.8982 | 9.29E+07 |
| 3 | 6.3 | 0 | 30 | 50 | 50 | 50.8982 | 1.16E+08 |
| 1 | 6.3 | 0 | 30 | 50 | 100 | 50.8982 | 1.63E+08 |
| 2 | 6.3 | 0 | 30 | 50 | 100 | 50.8982 | 1.59E+08 |
| 3 | 6.3 | 0 | 30 | 50 | 100 | 50.8982 | 1.53E+08 |
| 1 | 6.3 | 0 | 30 | 50 | 200 | 50.8982 | 9.55E+07 |
| 2 | 6.3 | 0 | 30 | 50 | 200 | 50.8982 | 8.47E+07 |
| 3 | 6.3 | 0 | 30 | 50 | 200 | 50.8982 | 1.16E+08 |
| 1 | 6.3 | 0 | 30 | 50 | 300 | 50.8982 | 7.76E+07 |
| 2 | 6.3 | 0 | 30 | 50 | 300 | 50.8982 | 8.85E+07 |
| 3 | 6.3 | 0 | 30 | 50 | 300 | 50.8982 | 9.50E+07 |
| 1 | 6.3 | 0 | 30 | 50 | 400 | 50.8982 | 4.22E+07 |
| 2 | 6.3 | 0 | 30 | 50 | 400 | 50.8982 | 5.30E+07 |
| 3 | 6.3 | 0 | 30 | 50 | 400 | 50.8982 | 4.77E+07 |
| 1 | 6.3 | 0 | 30 | 50 | 500 | 50.8982 | 4.30E+06 |
| 2 | 6.3 | 0 | 30 | 50 | 500 | 50.8982 | 5.11E+06 |
| 3 | 6.3 | 0 | 30 | 50 | 500 | 50.8982 | 4.99E+06 |
| 1 | 6.3 | 0.3 | 30 | 50 | 50 | 50.8982 | 4.33E+07 |
| 2 | 6.3 | 0.3 | 30 | 50 | 50 | 50.8982 | 4.80E+07 |
| 3 | 6.3 | 0.3 | 30 | 50 | 50 | 50.8982 | 4.50E+07 |
| 1 | 6.3 | 0.3 | 30 | 50 | 100 | 50.8982 | 5.94E+07 |
| 2 | 6.3 | 0.3 | 30 | 50 | 100 | 50.8982 | 4.88E+07 |
| 3 | 6.3 | 0.3 | 30 | 50 | 100 | 50.8982 | 5.37E+07 |
| 1 | 6.3 | 0.3 | 30 | 50 | 200 | 50.8982 | 5.33E+06 |
| 2 | 6.3 | 0.3 | 30 | 50 | 200 | 50.8982 | 5.89E+06 |
| 3 | 6.3 | 0.3 | 30 | 50 | 200 | 50.8982 | 6.60E+06 |
| 1 | 6.3 | 0.3 | 30 | 50 | 300 | 50.8982 | 3.30E+06 |
| 2 | 6.3 | 0.3 | 30 | 50 | 300 | 50.8982 | 3.37E+06 |
| 3 | 6.3 | 0.3 | 30 | 50 | 300 | 50.8982 | 3.51E+06 |
| 1 | 6.3 | 0.3 | 30 | 50 | 400 | 50.8982 | 3.08E+04 |
| 2 | 6.3 | 0.3 | 30 | 50 | 400 | 50.8982 | 3.45E+04 |
| 3 | 6.3 | 0.3 | 30 | 50 | 400 | 50.8982 | 3.17E+04 |
| 1 | 6.3 | 0.3 | 30 | 50 | 500 | 50.8982 | 0.00E+00 |
| 2 | 6.3 | 0.3 | 30 | 50 | 500 | 50.8982 | 0.00E+00 |
| 3 | 6.3 | 0.3 | 30 | 50 | 500 | 50.8982 | 0.00E+00 |
| 1 | 6.3 | 0.6 | 30 | 50 | 50 | 50.8982 | 3.32E+06 |
| 2 | 6.3 | 0.6 | 30 | 50 | 50 | 50.8982 | 3.29E+06 |
| 3 | 6.3 | 0.6 | 30 | 50 | 50 | 50.8982 | 3.17E+06 |
| 1 | 6.3 | 0.6 | 30 | 50 | 100 | 50.8982 | 3.47E+06 |
| 2 | 6.3 | 0.6 | 30 | 50 | 100 | 50.8982 | 2.48E+06 |
| 3 | 6.3 | 0.6 | 30 | 50 | 100 | 50.8982 | 2.62E+06 |
| 1 | 6.3 | 0.6 | 30 | 50 | 200 | 50.8982 | 2.44E+05 |
| 2 | 6.3 | 0.6 | 30 | 50 | 200 | 50.8982 | 2.30E+05 |
| 3 | 6.3 | 0.6 | 30 | 50 | 200 | 50.8982 | 2.35E+05 |
| 1 | 6.3 | 0.6 | 30 | 50 | 300 | 50.8982 | 3.95E+04 |
| 2 | 6.3 | 0.6 | 30 | 50 | 300 | 50.8982 | 2.35E+04 |
| 3 | 6.3 | 0.6 | 30 | 50 | 300 | 50.8982 | 2.41E+04 |
| 1 | 6.3 | 0.6 | 30 | 50 | 400 | 50.8982 | 3.37E+03 |
| 2 | 6.3 | 0.6 | 30 | 50 | 400 | 50.8982 | 4.38E+03 |
| 3 | 6.3 | 0.6 | 30 | 50 | 400 | 50.8982 | 4.73E+03 |
| 1 | 6.3 | 0.6 | 30 | 50 | 500 | 50.8982 | 0.00E+00 |
| 2 | 6.3 | 0.6 | 30 | 50 | 500 | 50.8982 | 0.00E+00 |
| 3 | 6.3 | 0.6 | 30 | 50 | 500 | 50.8982 | 0.00E+00 |
| 1 | 6.3 | 0.9 | 30 | 50 | 50 | 50.8982 | 3.11E+04 |
| 2 | 6.3 | 0.9 | 30 | 50 | 50 | 50.8982 | 2.99E+04 |
| 3 | 6.3 | 0.9 | 30 | 50 | 50 | 50.8982 | 3.20E+04 |
| 1 | 6.3 | 0.9 | 30 | 50 | 100 | 50.8982 | 5.50E+04 |
| 2 | 6.3 | 0.9 | 30 | 50 | 100 | 50.8982 | 4.60E+04 |
| 3 | 6.3 | 0.9 | 30 | 50 | 100 | 50.8982 | 5.11E+04 |
| 1 | 6.3 | 0.9 | 30 | 50 | 200 | 50.8982 | 3.94E+04 |
| 2 | 6.3 | 0.9 | 30 | 50 | 200 | 50.8982 | 3.85E+04 |
| 3 | 6.3 | 0.9 | 30 | 50 | 200 | 50.8982 | 4.16E+04 |
| 1 | 6.3 | 0.9 | 30 | 50 | 300 | 50.8982 | 0.00E+00 |
| 2 | 6.3 | 0.9 | 30 | 50 | 300 | 50.8982 | 0.00E+00 |
| 3 | 6.3 | 0.9 | 30 | 50 | 300 | 50.8982 | 0.00E+00 |
| 1 | 6.3 | 0.9 | 30 | 50 | 400 | 50.8982 | 0.00E+00 |
| 2 | 6.3 | 0.9 | 30 | 50 | 400 | 50.8982 | 0.00E+00 |
| 3 | 6.3 | 0.9 | 30 | 50 | 400 | 50.8982 | 0.00E+00 |
| 1 | 6.3 | 0.9 | 30 | 50 | 500 | 50.8982 | 0.00E+00 |
| 2 | 6.3 | 0.9 | 30 | 50 | 500 | 50.8982 | 0.00E+00 |
| 3 | 6.3 | 0.9 | 30 | 50 | 500 | 50.8982 | 0.00E+00 |
| 1 | 6.3 | 1.2 | 30 | 50 | 50 | 50.8982 | 3.43E+04 |
| 2 | 6.3 | 1.2 | 30 | 50 | 50 | 50.8982 | 3.30E+04 |
| 3 | 6.3 | 1.2 | 30 | 50 | 50 | 50.8982 | 3.22E+04 |
| 1 | 6.3 | 1.2 | 30 | 50 | 100 | 50.8982 | 2.44E+04 |
| 2 | 6.3 | 1.2 | 30 | 50 | 100 | 50.8982 | 3.50E+04 |
| 3 | 6.3 | 1.2 | 30 | 50 | 100 | 50.8982 | 3.37E+04 |
| 1 | 6.3 | 1.2 | 30 | 50 | 200 | 50.8982 | 4.43E+03 |
| 2 | 6.3 | 1.2 | 30 | 50 | 200 | 50.8982 | 3.75E+03 |
| 3 | 6.3 | 1.2 | 30 | 50 | 200 | 50.8982 | 4.29E+03 |
| 1 | 6.3 | 1.2 | 30 | 50 | 300 | 50.8982 | 0.00E+00 |
| 2 | 6.3 | 1.2 | 30 | 50 | 300 | 50.8982 | 0.00E+00 |
| 3 | 6.3 | 1.2 | 30 | 50 | 300 | 50.8982 | 0.00E+00 |
| 1 | 6.3 | 1.2 | 30 | 50 | 400 | 50.8982 | 0.00E+00 |
| 2 | 6.3 | 1.2 | 30 | 50 | 400 | 50.8982 | 0.00E+00 |
| 3 | 6.3 | 1.2 | 30 | 50 | 400 | 50.8982 | 0.00E+00 |
| 1 | 6.3 | 1.2 | 30 | 50 | 500 | 50.8982 | 0.00E+00 |
| 2 | 6.3 | 1.2 | 30 | 50 | 500 | 50.8982 | 0.00E+00 |
| 3 | 6.3 | 1.2 | 30 | 50 | 500 | 50.8982 | 0.00E+00 |
| 1 | 6.3 | 0 | 35 | 50 | 50 | 50.8982 | 7.49E+07 |
| 2 | 6.3 | 0 | 35 | 50 | 50 | 50.8982 | 8.90E+07 |
| 3 | 6.3 | 0 | 35 | 50 | 50 | 50.8982 | 8.53E+07 |
| 1 | 6.3 | 0 | 35 | 50 | 100 | 50.8982 | 1.67E+08 |
| 2 | 6.3 | 0 | 35 | 50 | 100 | 50.8982 | 1.37E+08 |
| 3 | 6.3 | 0 | 35 | 50 | 100 | 50.8982 | 1.52E+08 |
| 1 | 6.3 | 0 | 35 | 50 | 200 | 50.8982 | 1.83E+08 |
| 2 | 6.3 | 0 | 35 | 50 | 200 | 50.8982 | 1.33E+08 |
| 3 | 6.3 | 0 | 35 | 50 | 200 | 50.8982 | 1.48E+08 |
| 1 | 6.3 | 0 | 35 | 50 | 300 | 50.8982 | 1.03E+08 |
| 2 | 6.3 | 0 | 35 | 50 | 300 | 50.8982 | 9.73E+07 |
| 3 | 6.3 | 0 | 35 | 50 | 300 | 50.8982 | 1.18E+08 |
| 1 | 6.3 | 0 | 35 | 50 | 400 | 50.8982 | 5.92E+07 |
| 2 | 6.3 | 0 | 35 | 50 | 400 | 50.8982 | 4.29E+07 |
| 3 | 6.3 | 0 | 35 | 50 | 400 | 50.8982 | 5.80E+07 |
| 1 | 6.3 | 0 | 35 | 50 | 500 | 50.8982 | 2.44E+07 |
| 2 | 6.3 | 0 | 35 | 50 | 500 | 50.8982 | 2.87E+07 |
| 3 | 6.3 | 0 | 35 | 50 | 500 | 50.8982 | 2.50E+07 |
| 1 | 6.3 | 0.3 | 35 | 50 | 50 | 50.8982 | 2.60E+07 |
| 2 | 6.3 | 0.3 | 35 | 50 | 50 | 50.8982 | 2.93E+07 |
| 3 | 6.3 | 0.3 | 35 | 50 | 50 | 50.8982 | 2.87E+07 |
| 1 | 6.3 | 0.3 | 35 | 50 | 100 | 50.8982 | 4.72E+07 |
| 2 | 6.3 | 0.3 | 35 | 50 | 100 | 50.8982 | 5.50E+07 |
| 3 | 6.3 | 0.3 | 35 | 50 | 100 | 50.8982 | 4.38E+07 |
| 1 | 6.3 | 0.3 | 35 | 50 | 200 | 50.8982 | 4.73E+06 |
| 2 | 6.3 | 0.3 | 35 | 50 | 200 | 50.8982 | 3.64E+06 |
| 3 | 6.3 | 0.3 | 35 | 50 | 200 | 50.8982 | 3.39E+06 |
| 1 | 6.3 | 0.3 | 35 | 50 | 300 | 50.8982 | 4.37E+06 |
| 2 | 6.3 | 0.3 | 35 | 50 | 300 | 50.8982 | 3.32E+06 |
| 3 | 6.3 | 0.3 | 35 | 50 | 300 | 50.8982 | 4.17E+06 |
| 1 | 6.3 | 0.3 | 35 | 50 | 400 | 50.8982 | 3.33E+03 |
| 2 | 6.3 | 0.3 | 35 | 50 | 400 | 50.8982 | 1.58E+03 |
| 3 | 6.3 | 0.3 | 35 | 50 | 400 | 50.8982 | 2.90E+03 |
| 1 | 6.3 | 0.3 | 35 | 50 | 500 | 50.8982 | 0.00E+00 |
| 2 | 6.3 | 0.3 | 35 | 50 | 500 | 50.8982 | 0.00E+00 |
| 3 | 6.3 | 0.3 | 35 | 50 | 500 | 50.8982 | 0.00E+00 |
| 1 | 6.3 | 0.6 | 35 | 50 | 50 | 50.8982 | 2.97E+05 |
| 2 | 6.3 | 0.6 | 35 | 50 | 50 | 50.8982 | 1.55E+05 |
| 3 | 6.3 | 0.6 | 35 | 50 | 50 | 50.8982 | 2.49E+05 |
| 1 | 6.3 | 0.6 | 35 | 50 | 100 | 50.8982 | 4.50E+05 |
| 2 | 6.3 | 0.6 | 35 | 50 | 100 | 50.8982 | 5.07E+05 |
| 3 | 6.3 | 0.6 | 35 | 50 | 100 | 50.8982 | 6.15E+05 |
| 1 | 6.3 | 0.6 | 35 | 50 | 200 | 50.8982 | 6.32E+04 |
| 2 | 6.3 | 0.6 | 35 | 50 | 200 | 50.8982 | 5.70E+04 |
| 3 | 6.3 | 0.6 | 35 | 50 | 200 | 50.8982 | 5.53E+04 |
| 1 | 6.3 | 0.6 | 35 | 50 | 300 | 50.8982 | 3.18E+03 |
| 2 | 6.3 | 0.6 | 35 | 50 | 300 | 50.8982 | 3.85E+03 |
| 3 | 6.3 | 0.6 | 35 | 50 | 300 | 50.8982 | 4.11E+03 |
| 1 | 6.3 | 0.6 | 35 | 50 | 400 | 50.8982 | 1.14E+02 |
| 2 | 6.3 | 0.6 | 35 | 50 | 400 | 50.8982 | 1.34E+02 |
| 3 | 6.3 | 0.6 | 35 | 50 | 400 | 50.8982 | 1.20E+02 |
| 1 | 6.3 | 0.6 | 35 | 50 | 500 | 50.8982 | 0.00E+00 |
| 2 | 6.3 | 0.6 | 35 | 50 | 500 | 50.8982 | 0.00E+00 |
| 3 | 6.3 | 0.6 | 35 | 50 | 500 | 50.8982 | 0.00E+00 |
| 1 | 6.3 | 0.9 | 35 | 50 | 50 | 50.8982 | 3.67E+04 |
| 2 | 6.3 | 0.9 | 35 | 50 | 50 | 50.8982 | 3.38E+04 |
| 3 | 6.3 | 0.9 | 35 | 50 | 50 | 50.8982 | 3.33E+04 |
| 1 | 6.3 | 0.9 | 35 | 50 | 100 | 50.8982 | 2.56E+04 |
| 2 | 6.3 | 0.9 | 35 | 50 | 100 | 50.8982 | 3.00E+04 |
| 3 | 6.3 | 0.9 | 35 | 50 | 100 | 50.8982 | 2.41E+04 |
| 1 | 6.3 | 0.9 | 35 | 50 | 200 | 50.8982 | 5.25E+02 |
| 2 | 6.3 | 0.9 | 35 | 50 | 200 | 50.8982 | 5.50E+02 |
| 3 | 6.3 | 0.9 | 35 | 50 | 200 | 50.8982 | 4.00E+02 |
| 1 | 6.3 | 0.9 | 35 | 50 | 300 | 50.8982 | 0.00E+00 |
| 2 | 6.3 | 0.9 | 35 | 50 | 300 | 50.8982 | 0.00E+00 |
| 3 | 6.3 | 0.9 | 35 | 50 | 300 | 50.8982 | 0.00E+00 |
| 1 | 6.3 | 0.9 | 35 | 50 | 400 | 50.8982 | 0.00E+00 |
| 2 | 6.3 | 0.9 | 35 | 50 | 400 | 50.8982 | 0.00E+00 |
| 3 | 6.3 | 0.9 | 35 | 50 | 400 | 50.8982 | 0.00E+00 |
| 1 | 6.3 | 0.9 | 35 | 50 | 500 | 50.8982 | 0.00E+00 |
| 2 | 6.3 | 0.9 | 35 | 50 | 500 | 50.8982 | 0.00E+00 |
| 3 | 6.3 | 0.9 | 35 | 50 | 500 | 50.8982 | 0.00E+00 |
| 1 | 6.3 | 1.2 | 35 | 50 | 50 | 50.8982 | 3.63E+03 |
| 2 | 6.3 | 1.2 | 35 | 50 | 50 | 50.8982 | 3.03E+03 |
| 3 | 6.3 | 1.2 | 35 | 50 | 50 | 50.8982 | 3.12E+03 |
| 1 | 6.3 | 1.2 | 35 | 50 | 100 | 50.8982 | 4.44E+03 |
| 2 | 6.3 | 1.2 | 35 | 50 | 100 | 50.8982 | 3.71E+03 |
| 3 | 6.3 | 1.2 | 35 | 50 | 100 | 50.8982 | 3.80E+03 |
| 1 | 6.3 | 1.2 | 35 | 50 | 200 | 50.8982 | 2.77E+02 |
| 2 | 6.3 | 1.2 | 35 | 50 | 200 | 50.8982 | 1.98E+02 |
| 3 | 6.3 | 1.2 | 35 | 50 | 200 | 50.8982 | 1.60E+02 |
| 1 | 6.3 | 1.2 | 35 | 50 | 300 | 50.8982 | 0.00E+00 |
| 2 | 6.3 | 1.2 | 35 | 50 | 300 | 50.8982 | 0.00E+00 |
| 3 | 6.3 | 1.2 | 35 | 50 | 300 | 50.8982 | 0.00E+00 |
| 1 | 6.3 | 1.2 | 35 | 50 | 400 | 50.8982 | 0.00E+00 |
| 2 | 6.3 | 1.2 | 35 | 50 | 400 | 50.8982 | 0.00E+00 |
| 3 | 6.3 | 1.2 | 35 | 50 | 400 | 50.8982 | 0.00E+00 |
| 1 | 6.3 | 1.2 | 35 | 50 | 500 | 50.8982 | 0.00E+00 |
| 2 | 6.3 | 1.2 | 35 | 50 | 500 | 50.8982 | 0.00E+00 |
| 3 | 6.3 | 1.2 | 35 | 50 | 500 | 50.8982 | 0.00E+00 |
| 1 | 6.3 | 0 | 25 | 75 | 50 | 50.8982 | 1.17E+07 |
| 2 | 6.3 | 0 | 25 | 75 | 50 | 50.8982 | 9.83E+06 |
| 3 | 6.3 | 0 | 25 | 75 | 50 | 50.8982 | 8.98E+06 |
| 1 | 6.3 | 0 | 25 | 75 | 100 | 50.8982 | 1.21E+07 |
| 2 | 6.3 | 0 | 25 | 75 | 100 | 50.8982 | 8.87E+06 |
| 3 | 6.3 | 0 | 25 | 75 | 100 | 50.8982 | 1.10E+07 |
| 1 | 6.3 | 0 | 25 | 75 | 200 | 50.8982 | 7.45E+06 |
| 2 | 6.3 | 0 | 25 | 75 | 200 | 50.8982 | 9.30E+06 |
| 3 | 6.3 | 0 | 25 | 75 | 200 | 50.8982 | 8.32E+06 |
| 1 | 6.3 | 0 | 25 | 75 | 300 | 50.8982 | 7.60E+06 |
| 2 | 6.3 | 0 | 25 | 75 | 300 | 50.8982 | 7.89E+06 |
| 3 | 6.3 | 0 | 25 | 75 | 300 | 50.8982 | 8.30E+06 |
| 1 | 6.3 | 0 | 25 | 75 | 400 | 50.8982 | 3.24E+06 |
| 2 | 6.3 | 0 | 25 | 75 | 400 | 50.8982 | 2.09E+06 |
| 3 | 6.3 | 0 | 25 | 75 | 400 | 50.8982 | 3.18E+06 |
| 1 | 6.3 | 0 | 25 | 75 | 500 | 50.8982 | 1.91E+06 |
| 2 | 6.3 | 0 | 25 | 75 | 500 | 50.8982 | 8.97E+05 |
| 3 | 6.3 | 0 | 25 | 75 | 500 | 50.8982 | 9.33E+05 |
| 1 | 6.3 | 0.3 | 25 | 75 | 50 | 50.8982 | 2.90E+07 |
| 2 | 6.3 | 0.3 | 25 | 75 | 50 | 50.8982 | 2.18E+07 |
| 3 | 6.3 | 0.3 | 25 | 75 | 50 | 50.8982 | 2.10E+07 |
| 1 | 6.3 | 0.3 | 25 | 75 | 100 | 50.8982 | 7.11E+06 |
| 2 | 6.3 | 0.3 | 25 | 75 | 100 | 50.8982 | 7.50E+06 |
| 3 | 6.3 | 0.3 | 25 | 75 | 100 | 50.8982 | 8.50E+06 |
| 1 | 6.3 | 0.3 | 25 | 75 | 200 | 50.8982 | 5.30E+06 |
| 2 | 6.3 | 0.3 | 25 | 75 | 200 | 50.8982 | 7.60E+06 |
| 3 | 6.3 | 0.3 | 25 | 75 | 200 | 50.8982 | 6.22E+06 |
| 1 | 6.3 | 0.3 | 25 | 75 | 300 | 50.8982 | 4.42E+06 |
| 2 | 6.3 | 0.3 | 25 | 75 | 300 | 50.8982 | 3.88E+06 |
| 3 | 6.3 | 0.3 | 25 | 75 | 300 | 50.8982 | 4.55E+06 |
| 1 | 6.3 | 0.3 | 25 | 75 | 400 | 50.8982 | 3.71E+06 |
| 2 | 6.3 | 0.3 | 25 | 75 | 400 | 50.8982 | 1.62E+06 |
| 3 | 6.3 | 0.3 | 25 | 75 | 400 | 50.8982 | 2.55E+06 |
| 1 | 6.3 | 0.3 | 25 | 75 | 500 | 50.8982 | 6.40E+04 |
| 2 | 6.3 | 0.3 | 25 | 75 | 500 | 50.8982 | 8.83E+04 |
| 3 | 6.3 | 0.3 | 25 | 75 | 500 | 50.8982 | 7.24E+04 |
| 1 | 6.3 | 0.6 | 25 | 75 | 50 | 50.8982 | 1.21E+07 |
| 2 | 6.3 | 0.6 | 25 | 75 | 50 | 50.8982 | 9.93E+06 |
| 3 | 6.3 | 0.6 | 25 | 75 | 50 | 50.8982 | 1.01E+07 |
| 1 | 6.3 | 0.6 | 25 | 75 | 100 | 50.8982 | 8.47E+06 |
| 2 | 6.3 | 0.6 | 25 | 75 | 100 | 50.8982 | 9.33E+06 |
| 3 | 6.3 | 0.6 | 25 | 75 | 100 | 50.8982 | 8.74E+06 |
| 1 | 6.3 | 0.6 | 25 | 75 | 200 | 50.8982 | 6.44E+06 |
| 2 | 6.3 | 0.6 | 25 | 75 | 200 | 50.8982 | 5.88E+06 |
| 3 | 6.3 | 0.6 | 25 | 75 | 200 | 50.8982 | 5.43E+06 |
| 1 | 6.3 | 0.6 | 25 | 75 | 300 | 50.8982 | 9.60E+05 |
| 2 | 6.3 | 0.6 | 25 | 75 | 300 | 50.8982 | 9.22E+05 |
| 3 | 6.3 | 0.6 | 25 | 75 | 300 | 50.8982 | 8.30E+05 |
| 1 | 6.3 | 0.6 | 25 | 75 | 400 | 50.8982 | 5.44E+04 |
| 2 | 6.3 | 0.6 | 25 | 75 | 400 | 50.8982 | 6.54E+04 |
| 3 | 6.3 | 0.6 | 25 | 75 | 400 | 50.8982 | 6.22E+04 |
| 1 | 6.3 | 0.6 | 25 | 75 | 500 | 50.8982 | 1.35E+04 |
| 2 | 6.3 | 0.6 | 25 | 75 | 500 | 50.8982 | 2.26E+04 |
| 3 | 6.3 | 0.6 | 25 | 75 | 500 | 50.8982 | 1.88E+04 |
| 1 | 6.3 | 0.9 | 25 | 75 | 50 | 50.8982 | 7.10E+04 |
| 2 | 6.3 | 0.9 | 25 | 75 | 50 | 50.8982 | 5.80E+04 |
| 3 | 6.3 | 0.9 | 25 | 75 | 50 | 50.8982 | 7.89E+04 |
| 1 | 6.3 | 0.9 | 25 | 75 | 100 | 50.8982 | 5.90E+04 |
| 2 | 6.3 | 0.9 | 25 | 75 | 100 | 50.8982 | 6.63E+04 |
| 3 | 6.3 | 0.9 | 25 | 75 | 100 | 50.8982 | 6.60E+04 |
| 1 | 6.3 | 0.9 | 25 | 75 | 200 | 50.8982 | 2.07E+04 |
| 2 | 6.3 | 0.9 | 25 | 75 | 200 | 50.8982 | 2.04E+04 |
| 3 | 6.3 | 0.9 | 25 | 75 | 200 | 50.8982 | 1.99E+04 |
| 1 | 6.3 | 0.9 | 25 | 75 | 300 | 50.8982 | 5.30E+03 |
| 2 | 6.3 | 0.9 | 25 | 75 | 300 | 50.8982 | 6.54E+03 |
| 3 | 6.3 | 0.9 | 25 | 75 | 300 | 50.8982 | 6.12E+03 |
| 1 | 6.3 | 0.9 | 25 | 75 | 400 | 50.8982 | 0.00E+00 |
| 2 | 6.3 | 0.9 | 25 | 75 | 400 | 50.8982 | 0.00E+00 |
| 3 | 6.3 | 0.9 | 25 | 75 | 400 | 50.8982 | 0.00E+00 |
| 1 | 6.3 | 0.9 | 25 | 75 | 500 | 50.8982 | 0.00E+00 |
| 2 | 6.3 | 0.9 | 25 | 75 | 500 | 50.8982 | 0.00E+00 |
| 3 | 6.3 | 0.9 | 25 | 75 | 500 | 50.8982 | 0.00E+00 |
| 1 | 6.3 | 1.2 | 25 | 75 | 50 | 50.8982 | 1.39E+04 |
| 2 | 6.3 | 1.2 | 25 | 75 | 50 | 50.8982 | 1.70E+04 |
| 3 | 6.3 | 1.2 | 25 | 75 | 50 | 50.8982 | 1.56E+04 |
| 1 | 6.3 | 1.2 | 25 | 75 | 100 | 50.8982 | 2.04E+04 |
| 2 | 6.3 | 1.2 | 25 | 75 | 100 | 50.8982 | 2.25E+04 |
| 3 | 6.3 | 1.2 | 25 | 75 | 100 | 50.8982 | 2.11E+04 |
| 1 | 6.3 | 1.2 | 25 | 75 | 200 | 50.8982 | 7.90E+03 |
| 2 | 6.3 | 1.2 | 25 | 75 | 200 | 50.8982 | 7.54E+03 |
| 3 | 6.3 | 1.2 | 25 | 75 | 200 | 50.8982 | 7.70E+03 |
| 1 | 6.3 | 1.2 | 25 | 75 | 300 | 50.8982 | 1.05E+03 |
| 2 | 6.3 | 1.2 | 25 | 75 | 300 | 50.8982 | 8.00E+02 |
| 3 | 6.3 | 1.2 | 25 | 75 | 300 | 50.8982 | 9.57E+02 |
| 1 | 6.3 | 1.2 | 25 | 75 | 400 | 50.8982 | 0.00E+00 |
| 2 | 6.3 | 1.2 | 25 | 75 | 400 | 50.8982 | 0.00E+00 |
| 3 | 6.3 | 1.2 | 25 | 75 | 400 | 50.8982 | 0.00E+00 |
| 1 | 6.3 | 1.2 | 25 | 75 | 500 | 50.8982 | 0.00E+00 |
| 2 | 6.3 | 1.2 | 25 | 75 | 500 | 50.8982 | 0.00E+00 |
| 3 | 6.3 | 1.2 | 25 | 75 | 500 | 50.8982 | 0.00E+00 |
| 1 | 6.3 | 0 | 30 | 75 | 50 | 50.8982 | 4.37E+08 |
| 2 | 6.3 | 0 | 30 | 75 | 50 | 50.8982 | 3.36E+08 |
| 3 | 6.3 | 0 | 30 | 75 | 50 | 50.8982 | 3.80E+08 |
| 1 | 6.3 | 0 | 30 | 75 | 100 | 50.8982 | 7.67E+08 |
| 2 | 6.3 | 0 | 30 | 75 | 100 | 50.8982 | 6.53E+08 |
| 3 | 6.3 | 0 | 30 | 75 | 100 | 50.8982 | 7.12E+08 |
| 1 | 6.3 | 0 | 30 | 75 | 200 | 50.8982 | 6.24E+08 |
| 2 | 6.3 | 0 | 30 | 75 | 200 | 50.8982 | 7.61E+08 |
| 3 | 6.3 | 0 | 30 | 75 | 200 | 50.8982 | 7.25E+08 |
| 1 | 6.3 | 0 | 30 | 75 | 300 | 50.8982 | 2.54E+08 |
| 2 | 6.3 | 0 | 30 | 75 | 300 | 50.8982 | 2.09E+08 |
| 3 | 6.3 | 0 | 30 | 75 | 300 | 50.8982 | 2.73E+08 |
| 1 | 6.3 | 0 | 30 | 75 | 400 | 50.8982 | 1.91E+08 |
| 2 | 6.3 | 0 | 30 | 75 | 400 | 50.8982 | 2.40E+08 |
| 3 | 6.3 | 0 | 30 | 75 | 400 | 50.8982 | 2.35E+08 |
| 1 | 6.3 | 0 | 30 | 75 | 500 | 50.8982 | 5.81E+07 |
| 2 | 6.3 | 0 | 30 | 75 | 500 | 50.8982 | 5.89E+07 |
| 3 | 6.3 | 0 | 30 | 75 | 500 | 50.8982 | 5.57E+07 |
| 1 | 6.3 | 0.3 | 30 | 75 | 50 | 50.8982 | 5.84E+07 |
| 2 | 6.3 | 0.3 | 30 | 75 | 50 | 50.8982 | 5.14E+07 |
| 3 | 6.3 | 0.3 | 30 | 75 | 50 | 50.8982 | 5.22E+07 |
| 1 | 6.3 | 0.3 | 30 | 75 | 100 | 50.8982 | 5.21E+07 |
| 2 | 6.3 | 0.3 | 30 | 75 | 100 | 50.8982 | 4.11E+07 |
| 3 | 6.3 | 0.3 | 30 | 75 | 100 | 50.8982 | 4.83E+07 |
| 1 | 6.3 | 0.3 | 30 | 75 | 200 | 50.8982 | 6.62E+07 |
| 2 | 6.3 | 0.3 | 30 | 75 | 200 | 50.8982 | 5.30E+07 |
| 3 | 6.3 | 0.3 | 30 | 75 | 200 | 50.8982 | 5.70E+07 |
| 1 | 6.3 | 0.3 | 30 | 75 | 300 | 50.8982 | 4.13E+07 |
| 2 | 6.3 | 0.3 | 30 | 75 | 300 | 50.8982 | 3.61E+07 |
| 3 | 6.3 | 0.3 | 30 | 75 | 300 | 50.8982 | 3.82E+07 |
| 1 | 6.3 | 0.3 | 30 | 75 | 400 | 50.8982 | 5.00E+06 |
| 2 | 6.3 | 0.3 | 30 | 75 | 400 | 50.8982 | 4.50E+06 |
| 3 | 6.3 | 0.3 | 30 | 75 | 400 | 50.8982 | 4.78E+06 |
| 1 | 6.3 | 0.3 | 30 | 75 | 500 | 50.8982 | 6.67E+05 |
| 2 | 6.3 | 0.3 | 30 | 75 | 500 | 50.8982 | 5.54E+05 |
| 3 | 6.3 | 0.3 | 30 | 75 | 500 | 50.8982 | 6.30E+05 |
| 1 | 6.3 | 0.6 | 30 | 75 | 50 | 50.8982 | 2.06E+07 |
| 2 | 6.3 | 0.6 | 30 | 75 | 50 | 50.8982 | 2.18E+07 |
| 3 | 6.3 | 0.6 | 30 | 75 | 50 | 50.8982 | 2.24E+07 |
| 1 | 6.3 | 0.6 | 30 | 75 | 100 | 50.8982 | 1.09E+07 |
| 2 | 6.3 | 0.6 | 30 | 75 | 100 | 50.8982 | 1.25E+07 |
| 3 | 6.3 | 0.6 | 30 | 75 | 100 | 50.8982 | 1.14E+07 |
| 1 | 6.3 | 0.6 | 30 | 75 | 200 | 50.8982 | 4.55E+06 |
| 2 | 6.3 | 0.6 | 30 | 75 | 200 | 50.8982 | 3.91E+06 |
| 3 | 6.3 | 0.6 | 30 | 75 | 200 | 50.8982 | 4.12E+06 |
| 1 | 6.3 | 0.6 | 30 | 75 | 300 | 50.8982 | 7.63E+05 |
| 2 | 6.3 | 0.6 | 30 | 75 | 300 | 50.8982 | 6.32E+05 |
| 3 | 6.3 | 0.6 | 30 | 75 | 300 | 50.8982 | 7.70E+05 |
| 1 | 6.3 | 0.6 | 30 | 75 | 400 | 50.8982 | 2.83E+05 |
| 2 | 6.3 | 0.6 | 30 | 75 | 400 | 50.8982 | 3.37E+05 |
| 3 | 6.3 | 0.6 | 30 | 75 | 400 | 50.8982 | 3.80E+05 |
| 1 | 6.3 | 0.6 | 30 | 75 | 500 | 50.8982 | 2.52E+04 |
| 2 | 6.3 | 0.6 | 30 | 75 | 500 | 50.8982 | 1.74E+04 |
| 3 | 6.3 | 0.6 | 30 | 75 | 500 | 50.8982 | 2.16E+04 |
| 1 | 6.3 | 0.9 | 30 | 75 | 50 | 50.8982 | 3.38E+04 |
| 2 | 6.3 | 0.9 | 30 | 75 | 50 | 50.8982 | 4.19E+04 |
| 3 | 6.3 | 0.9 | 30 | 75 | 50 | 50.8982 | 4.42E+04 |
| 1 | 6.3 | 0.9 | 30 | 75 | 100 | 50.8982 | 6.31E+04 |
| 2 | 6.3 | 0.9 | 30 | 75 | 100 | 50.8982 | 7.50E+04 |
| 3 | 6.3 | 0.9 | 30 | 75 | 100 | 50.8982 | 6.86E+04 |
| 1 | 6.3 | 0.9 | 30 | 75 | 200 | 50.8982 | 2.07E+04 |
| 2 | 6.3 | 0.9 | 30 | 75 | 200 | 50.8982 | 3.30E+04 |
| 3 | 6.3 | 0.9 | 30 | 75 | 200 | 50.8982 | 2.83E+04 |
| 1 | 6.3 | 0.9 | 30 | 75 | 300 | 50.8982 | 1.19E+04 |
| 2 | 6.3 | 0.9 | 30 | 75 | 300 | 50.8982 | 1.36E+04 |
| 3 | 6.3 | 0.9 | 30 | 75 | 300 | 50.8982 | 1.62E+04 |
| 1 | 6.3 | 0.9 | 30 | 75 | 400 | 50.8982 | 0.00E+00 |
| 2 | 6.3 | 0.9 | 30 | 75 | 400 | 50.8982 | 0.00E+00 |
| 3 | 6.3 | 0.9 | 30 | 75 | 400 | 50.8982 | 0.00E+00 |
| 1 | 6.3 | 0.9 | 30 | 75 | 500 | 50.8982 | 0.00E+00 |
| 2 | 6.3 | 0.9 | 30 | 75 | 500 | 50.8982 | 0.00E+00 |
| 3 | 6.3 | 0.9 | 30 | 75 | 500 | 50.8982 | 0.00E+00 |
| 1 | 6.3 | 1.2 | 30 | 75 | 50 | 50.8982 | 3.09E+04 |
| 2 | 6.3 | 1.2 | 30 | 75 | 50 | 50.8982 | 5.20E+04 |
| 3 | 6.3 | 1.2 | 30 | 75 | 50 | 50.8982 | 4.22E+04 |
| 1 | 6.3 | 1.2 | 30 | 75 | 100 | 50.8982 | 6.52E+04 |
| 2 | 6.3 | 1.2 | 30 | 75 | 100 | 50.8982 | 7.41E+04 |
| 3 | 6.3 | 1.2 | 30 | 75 | 100 | 50.8982 | 6.12E+04 |
| 1 | 6.3 | 1.2 | 30 | 75 | 200 | 50.8982 | 4.71E+04 |
| 2 | 6.3 | 1.2 | 30 | 75 | 200 | 50.8982 | 3.78E+04 |
| 3 | 6.3 | 1.2 | 30 | 75 | 200 | 50.8982 | 4.20E+04 |
| 1 | 6.3 | 1.2 | 30 | 75 | 300 | 50.8982 | 1.02E+04 |
| 2 | 6.3 | 1.2 | 30 | 75 | 300 | 50.8982 | 1.17E+04 |
| 3 | 6.3 | 1.2 | 30 | 75 | 300 | 50.8982 | 1.65E+04 |
| 1 | 6.3 | 1.2 | 30 | 75 | 400 | 50.8982 | 0.00E+00 |
| 2 | 6.3 | 1.2 | 30 | 75 | 400 | 50.8982 | 0.00E+00 |
| 3 | 6.3 | 1.2 | 30 | 75 | 400 | 50.8982 | 0.00E+00 |
| 1 | 6.3 | 1.2 | 30 | 75 | 500 | 50.8982 | 0.00E+00 |
| 2 | 6.3 | 1.2 | 30 | 75 | 500 | 50.8982 | 0.00E+00 |
| 3 | 6.3 | 1.2 | 30 | 75 | 500 | 50.8982 | 0.00E+00 |
| 1 | 6.3 | 0 | 35 | 75 | 50 | 50.8982 | 1.38E+08 |
| 2 | 6.3 | 0 | 35 | 75 | 50 | 50.8982 | 1.57E+08 |
| 3 | 6.3 | 0 | 35 | 75 | 50 | 50.8982 | 1.60E+08 |
| 1 | 6.3 | 0 | 35 | 75 | 100 | 50.8982 | 1.16E+08 |
| 2 | 6.3 | 0 | 35 | 75 | 100 | 50.8982 | 1.11E+08 |
| 3 | 6.3 | 0 | 35 | 75 | 100 | 50.8982 | 1.10E+08 |
| 1 | 6.3 | 0 | 35 | 75 | 200 | 50.8982 | 1.66E+08 |
| 2 | 6.3 | 0 | 35 | 75 | 200 | 50.8982 | 1.40E+08 |
| 3 | 6.3 | 0 | 35 | 75 | 200 | 50.8982 | 1.45E+08 |
| 1 | 6.3 | 0 | 35 | 75 | 300 | 50.8982 | 1.25E+08 |
| 2 | 6.3 | 0 | 35 | 75 | 300 | 50.8982 | 1.70E+08 |
| 3 | 6.3 | 0 | 35 | 75 | 300 | 50.8982 | 1.46E+08 |
| 1 | 6.3 | 0 | 35 | 75 | 400 | 50.8982 | 1.19E+08 |
| 2 | 6.3 | 0 | 35 | 75 | 400 | 50.8982 | 8.55E+07 |
| 3 | 6.3 | 0 | 35 | 75 | 400 | 50.8982 | 8.37E+07 |
| 1 | 6.3 | 0 | 35 | 75 | 500 | 50.8982 | 5.89E+07 |
| 2 | 6.3 | 0 | 35 | 75 | 500 | 50.8982 | 5.66E+07 |
| 3 | 6.3 | 0 | 35 | 75 | 500 | 50.8982 | 6.12E+07 |
| 1 | 6.3 | 0.3 | 35 | 75 | 50 | 50.8982 | 5.15E+07 |
| 2 | 6.3 | 0.3 | 35 | 75 | 50 | 50.8982 | 5.07E+07 |
| 3 | 6.3 | 0.3 | 35 | 75 | 50 | 50.8982 | 5.20E+07 |
| 1 | 6.3 | 0.3 | 35 | 75 | 100 | 50.8982 | 5.56E+07 |
| 2 | 6.3 | 0.3 | 35 | 75 | 100 | 50.8982 | 6.13E+07 |
| 3 | 6.3 | 0.3 | 35 | 75 | 100 | 50.8982 | 5.71E+07 |
| 1 | 6.3 | 0.3 | 35 | 75 | 200 | 50.8982 | 6.11E+07 |
| 2 | 6.3 | 0.3 | 35 | 75 | 200 | 50.8982 | 5.33E+07 |
| 3 | 6.3 | 0.3 | 35 | 75 | 200 | 50.8982 | 5.80E+07 |
| 1 | 6.3 | 0.3 | 35 | 75 | 300 | 50.8982 | 2.11E+07 |
| 2 | 6.3 | 0.3 | 35 | 75 | 300 | 50.8982 | 2.07E+07 |
| 3 | 6.3 | 0.3 | 35 | 75 | 300 | 50.8982 | 2.25E+07 |
| 1 | 6.3 | 0.3 | 35 | 75 | 400 | 50.8982 | 8.53E+06 |
| 2 | 6.3 | 0.3 | 35 | 75 | 400 | 50.8982 | 7.20E+06 |
| 3 | 6.3 | 0.3 | 35 | 75 | 400 | 50.8982 | 8.22E+06 |
| 1 | 6.3 | 0.3 | 35 | 75 | 500 | 50.8982 | 6.38E+05 |
| 2 | 6.3 | 0.3 | 35 | 75 | 500 | 50.8982 | 6.90E+05 |
| 3 | 6.3 | 0.3 | 35 | 75 | 500 | 50.8982 | 6.40E+05 |
| 1 | 6.3 | 0.6 | 35 | 75 | 50 | 50.8982 | 1.65E+07 |
| 2 | 6.3 | 0.6 | 35 | 75 | 50 | 50.8982 | 1.22E+07 |
| 3 | 6.3 | 0.6 | 35 | 75 | 50 | 50.8982 | 1.57E+07 |
| 1 | 6.3 | 0.6 | 35 | 75 | 100 | 50.8982 | 8.44E+06 |
| 2 | 6.3 | 0.6 | 35 | 75 | 100 | 50.8982 | 7.71E+06 |
| 3 | 6.3 | 0.6 | 35 | 75 | 100 | 50.8982 | 8.17E+06 |
| 1 | 6.3 | 0.6 | 35 | 75 | 200 | 50.8982 | 7.07E+06 |
| 2 | 6.3 | 0.6 | 35 | 75 | 200 | 50.8982 | 7.21E+06 |
| 3 | 6.3 | 0.6 | 35 | 75 | 200 | 50.8982 | 6.32E+06 |
| 1 | 6.3 | 0.6 | 35 | 75 | 300 | 50.8982 | 7.44E+05 |
| 2 | 6.3 | 0.6 | 35 | 75 | 300 | 50.8982 | 8.99E+05 |
| 3 | 6.3 | 0.6 | 35 | 75 | 300 | 50.8982 | 8.30E+05 |
| 1 | 6.3 | 0.6 | 35 | 75 | 400 | 50.8982 | 2.15E+05 |
| 2 | 6.3 | 0.6 | 35 | 75 | 400 | 50.8982 | 1.88E+05 |
| 3 | 6.3 | 0.6 | 35 | 75 | 400 | 50.8982 | 1.72E+05 |
| 1 | 6.3 | 0.6 | 35 | 75 | 500 | 50.8982 | 9.88E+03 |
| 2 | 6.3 | 0.6 | 35 | 75 | 500 | 50.8982 | 8.71E+03 |
| 3 | 6.3 | 0.6 | 35 | 75 | 500 | 50.8982 | 9.21E+03 |
| 1 | 6.3 | 0.9 | 35 | 75 | 50 | 50.8982 | 7.73E+04 |
| 2 | 6.3 | 0.9 | 35 | 75 | 50 | 50.8982 | 8.25E+04 |
| 3 | 6.3 | 0.9 | 35 | 75 | 50 | 50.8982 | 7.49E+04 |
| 1 | 6.3 | 0.9 | 35 | 75 | 100 | 50.8982 | 5.64E+04 |
| 2 | 6.3 | 0.9 | 35 | 75 | 100 | 50.8982 | 6.87E+04 |
| 3 | 6.3 | 0.9 | 35 | 75 | 100 | 50.8982 | 6.12E+04 |
| 1 | 6.3 | 0.9 | 35 | 75 | 200 | 50.8982 | 8.42E+04 |
| 2 | 6.3 | 0.9 | 35 | 75 | 200 | 50.8982 | 7.62E+04 |
| 3 | 6.3 | 0.9 | 35 | 75 | 200 | 50.8982 | 8.10E+04 |
| 1 | 6.3 | 0.9 | 35 | 75 | 300 | 50.8982 | 3.10E+04 |
| 2 | 6.3 | 0.9 | 35 | 75 | 300 | 50.8982 | 2.21E+04 |
| 3 | 6.3 | 0.9 | 35 | 75 | 300 | 50.8982 | 3.39E+04 |
| 1 | 6.3 | 0.9 | 35 | 75 | 400 | 50.8982 | 5.11E+04 |
| 2 | 6.3 | 0.9 | 35 | 75 | 400 | 50.8982 | 4.31E+04 |
| 3 | 6.3 | 0.9 | 35 | 75 | 400 | 50.8982 | 4.63E+04 |
| 1 | 6.3 | 0.9 | 35 | 75 | 500 | 50.8982 | 0.00E+00 |
| 2 | 6.3 | 0.9 | 35 | 75 | 500 | 50.8982 | 0.00E+00 |
| 3 | 6.3 | 0.9 | 35 | 75 | 500 | 50.8982 | 0.00E+00 |
| 1 | 6.3 | 1.2 | 35 | 75 | 50 | 50.8982 | 5.42E+03 |
| 2 | 6.3 | 1.2 | 35 | 75 | 50 | 50.8982 | 5.69E+03 |
| 3 | 6.3 | 1.2 | 35 | 75 | 50 | 50.8982 | 5.17E+03 |
| 1 | 6.3 | 1.2 | 35 | 75 | 100 | 50.8982 | 5.45E+03 |
| 2 | 6.3 | 1.2 | 35 | 75 | 100 | 50.8982 | 6.33E+03 |
| 3 | 6.3 | 1.2 | 35 | 75 | 100 | 50.8982 | 5.20E+03 |
| 1 | 6.3 | 1.2 | 35 | 75 | 200 | 50.8982 | 8.61E+03 |
| 2 | 6.3 | 1.2 | 35 | 75 | 200 | 50.8982 | 8.25E+03 |
| 3 | 6.3 | 1.2 | 35 | 75 | 200 | 50.8982 | 8.93E+03 |
| 1 | 6.3 | 1.2 | 35 | 75 | 300 | 50.8982 | 5.11E+04 |
| 2 | 6.3 | 1.2 | 35 | 75 | 300 | 50.8982 | 6.33E+04 |
| 3 | 6.3 | 1.2 | 35 | 75 | 300 | 50.8982 | 5.70E+04 |
| 1 | 6.3 | 1.2 | 35 | 75 | 400 | 50.8982 | 0.00E+00 |
| 2 | 6.3 | 1.2 | 35 | 75 | 400 | 50.8982 | 0.00E+00 |
| 3 | 6.3 | 1.2 | 35 | 75 | 400 | 50.8982 | 0.00E+00 |
| 1 | 6.3 | 1.2 | 35 | 75 | 500 | 50.8982 | 0.00E+00 |
| 2 | 6.3 | 1.2 | 35 | 75 | 500 | 50.8982 | 0.00E+00 |
| 3 | 6.3 | 1.2 | 35 | 75 | 500 | 50.8982 | 0.00E+00 |
